# Pharmacogenomics of antibacterial and antiviral therapies: Clinical actionability, evidence gaps, and future directions

**DOI:** 10.1016/j.pharmr.2026.100136

**Published:** 2026-04-10

**Authors:** Maria K. Smatti, Zainab Jan, Hadi M. Yassine

**Affiliations:** 1 Biomedical Research Center, QU Health, Qatar University, Doha, Qatar; 2 College of Health and Life Sciences, Hamad Bin Khalifa University, Doha, Qatar; 3 Biomedical Sciences Department, College of Health Sciences, Qatar University, Doha, Qatar

## Abstract

Anti-infective drugs have profoundly transformed the history of medicine. Yet, with the presence of approximately 4.1–5 million interindividual genomic variants in human genome, patients are expected not to respond equally to the same anti-infective drug. This genetic variability, together with nongenetic factors, influences therapeutic outcomes and contributes to drug-induced adverse events in predisposed individuals. Historically, the identification of *HLA-B∗*57:01 as a predictor of abacavir hypersensitivity in patients with HIV represented the first successful clinical application of pharmacogenomics (PGx) in infectious diseases. Since then, the field has continued to evolve, as evidenced by the discovery of multiple clinically relevant gene–drug pairs, primarily related to immune responses, drug metabolism, and drug transport pathways. The evidence accumulated to date has established a number of mandatory (*HLA-B*∗57:01–abacavir) and actionable (*MT-RNR*1–aminoglycosides, *CYP2B6*–efavirenz, *G6PD*–nitrofurantoin, *G6PD*–nalidixic acid, and *G6PD*–dapsone) gene–drug pairs, whereas most other associations remain informative or exploratory without current guideline-based prescribing recommendations. Despite this progress, robust PGx evidence remains predominantly focused on antiretrovirals, anti–hepatitis C virus, and selected antimicrobial drug classes, such as *β*-lactams, aminoglycosides, sulfonamides, and antituberculosis drugs. For many other anti-infective agents, current evidence suggests that host genetic variation may have a limited impact on drug efficacy or safety, or that existing studies remain insufficiently powered or replicated to support clinical translation. The narrow ancestral diversity in PGx studies and clinical trials has also restricted the breadth of the knowledge gained and, consequently, the development of inclusive guidelines. This review summarizes the current PGx landscape of antibacterial and antiviral drugs and highlights key challenges and opportunities to improve clinical actionability. Greater inclusion of previously underrepresented populations, coupled with integrative multiomics approaches powered by artificial intelligence and machine learning, could accelerate PGx biomarker identification, validation, and integration into personalized patient care.

**Significance Statement:**

Rapid and effective deployment of anti-infective drugs requires incorporating knowledge of host genetic determinants of drug effectiveness or adverse events, the latter of which is a leading cause of death. The evolution of data science, driven by available genomic data, represents an unprecedented opportunity to accelerate pharmacogenomics discovery in infectious diseases. If acquired at a population level and integrated into medical records, pharmacogenomics data can guide the prescription and/or dosing of anti-infective drugs, shifting infectious disease management toward personalized care.

## I Introduction

In 2021, the Center for Drug Evaluation and Research of the US Food and Drug Administration (FDA) approved 50 drugs, 66% of which had underlying genetic evidence.^1^ Interindividual variability in clinical responses to administered drugs has often been challenging for dose adjustment, as most drugs are effective in only 25%–60% of patients.^2^ The estimated rates of drug unresponsiveness are 38%, 40%, 43%, 50%, and 75% in depression, asthma, diabetes, arthritis, and cancer medications, respectively.^2^ Moreover, most drug and vaccine candidates fail to reach approval, with success rates varying significantly across therapeutic areas. A study analyzing 406,038 clinical trials and over 21,000 compounds reported an overall success probability of 13.8% for all drugs and vaccines. Although drugs targeting infectious diseases (IDs) demonstrated the highest success rates, approximately 75% still fail to achieve approval.^3^

Pharmacogenomics (PGx) is a key pillar of precision medicine, aiming to develop targeted therapies based on patients’ genetic makeup, which influences drug metabolism, efficacy, and safety. Although closely related to pharmacogenetics—the study of specific genetic variations’ effects on drug metabolism and response—PGx encompasses a broader scope by probing the entire human genome to identify regions that collectively influence response to medication, also called the “pharmacogenome.”^4^ This holistic approach offers valuable insights into the mechanisms underlying variations in drug efficacy and safety between individuals and populations.

PGx can influence both drug pharmacokinetics (PK) and pharmacodynamics (PD). PK refers to the study of the absorption, distribution, metabolism, and excretion of drugs, whereas PD focuses on drug effects and their relationship to plasma concentration.^5^ Drug–gene interactions occur when specific genetic variations alter the PK and/or PD of a drug, influencing drug efficacy or increasing the risk of adverse drug reactions (ADRs).^6^ These interactions form the foundation of PGx by linking genetic variants to clinical outcomes.

The concept that drug response is influenced by genetic factors originated in the 1950s, following observations of ethnic differences in drug responses. It was observed that after administration of the antimalarial drug primaquine, approximately 10% of African Americans experienced hemolytic anemia, an ADR that was rarely observed in individuals of European ancestry.^7^ The role of genetics in drug response has been recognized by classical human genetics approaches, including twin and family studies, candidate gene studies, and randomized controlled trials.^4^^,^^8^ Over the past decades, PGx has evolved from an emerging scientific discipline to a well established interdisciplinary field. Driven by methodological development, reduced genotyping costs, completion of population genomics projects, and advances in biostatistics and bioinformatics tools, the PGx field continued to expand. Notably, PGx has been transformative through improving patient outcomes and guiding personalized therapy across various fields, including oncology (eg, thiopurine methyltransferase and nudix hydrolase 15 genotyping for thiopurine therapy and dihydropyrimidine dehydrogenase for fluoropyrimidine therapy), psychiatry (eg, *HLA-B*∗15:02 screening for carbamazepine use), and cardiology (eg, clopidogrel and *CYP2C19*).^9^, ^10^, ^11^

The practical application of PGx in clinical settings has been further facilitated by the availability of well structured, curated databases that provide easy access to data on gene–drug interactions and evidence-based guidelines. For instance, the Pharmacogenomics Knowledgebase (PharmGKB) compiles extensive data on how genetic variations affect drug responses. This information is categorized into prescribing information, drug label annotations, curated pathways, clinical annotations, and variant annotations. The variant annotation section describes the relationship between a genetic variant and a medication based on individual publications, whereas the clinical annotations section consolidates all such published evidence. By assigning scores and levels of evidence to each genetic variant-drug combination, PharmGKB helps prioritize genes for testing (with current data accessible through the ClinPGx platform).^12^ Additionally, the drug label annotations and prescribing information sections provide practical guidance on genetic testing requirements and interpretation of test results. As of August 28, 2024, the PharmGKB database included 27,865 variant annotations, 5163 clinical annotations, and 1090 drug label annotations, of which 1398, 219, and 56 are related to antiviral drugs, whereas 336, 54, and 6 are related to antibiotics, respectively. However, despite their comprehensiveness, a considerable proportion of these annotations are based on limited or nonactionable evidence, which may obscure clinically relevant, guideline-supported gene–drug pairs and complicate their implementation in practice. Therefore, careful prioritization of variants based on clinical validity, strength of evidence, and guideline support remains essential to ensure effective implementation of PGx in clinical care.

This review summarizes pharmacogenetic evidence related to antibacterial and antiviral drug response, with an emphasis on drug–gene pairs supported by strong scientific evidence and clinical relevance. It also discusses the challenges and opportunities for bridging existing gaps in ID PGx by leveraging advanced machine learning (ML) approaches and promoting inclusivity in research and clinical implementation.

## II Current burden and challenges in infectious diseases management

### A Burden of infectious diseases

Approximately one person dies from an infectious disease every 10 seconds.^13^ Over the last 5 decades, IDs have remained among the top 5 leading causes of mortality. In 2021, the newly emerged severe acute respiratory syndrome coronavirus 2 (SARS-CoV-2) alone was directly responsible for 8.8 million deaths, ranking as the second leading cause of death globally. Moreover, lower respiratory infections remained the deadliest communicable diseases apart from COVID-19, as they ranked the fifth leading cause of death in the same year, with approximately 2.5 million deaths. However, this was 370,000 lower than in 2020.^14^ Notably, despite the reduction in the overall number of people dying from IDs each year, the world continues to face recurrent outbreaks and pandemics caused by emerging or re-emerging pathogens. This includes respiratory viruses such as SARS-CoV, SARS-CoV-2, and Middle East respiratory syndrome coronavirus, vector-borne viruses such as Zika and Ebola, and other infections of global significance such as HIV and Mpox. In fact, the world has witnessed triple the number of outbreaks per year since 1980.^15^ Moreover, it is estimated that approximately 50% of the global population remains susceptible to emerging and re-emerging IDs.^16^ Communicable diseases disproportionately affect individuals in low-income countries, accounting for 8 of the top 10 causes of death in 2021.^14^ The leading cause of disease burden in these areas is the so-called “big three,” which are HIV/AIDS, tuberculosis (TB), and malaria.^13^ In addition, the global spread of antimicrobial resistance remains alarmingly high. In low- and middle-income countries, antimicrobial resistance is particularly catastrophic at the individual, household, and community levels, given the poor sanitation and weak healthcare systems.^17^ Some research groups have warned that by 2050, IDs could potentially surpass cardiovascular diseases as the world’s number one killer and lead to healthcare costs of at least $100 trillion.^18^

Tackling the significant burden of IDs has largely relied on the mass distribution of vaccines and new or repurposed anti-infective agents, including antivirals, antibiotics, and antituberculosis drugs. However, key challenges remain regarding the efficacy and safety of these drugs, which are both pathogen- and host-dependent. Resistance development driven by misuse, prolonged use, repeated courses, or failure to account for interindividual variability, especially in immunocompromised patients, further complicates treatment. Of note, there is still limited knowledge on the contribution of host factors (including genetics) to the safety profiles of these drugs, particularly among the underrepresented populations, which have different socioeconomic, epidemiological, and genetic architectures.

**Fig. 1:**
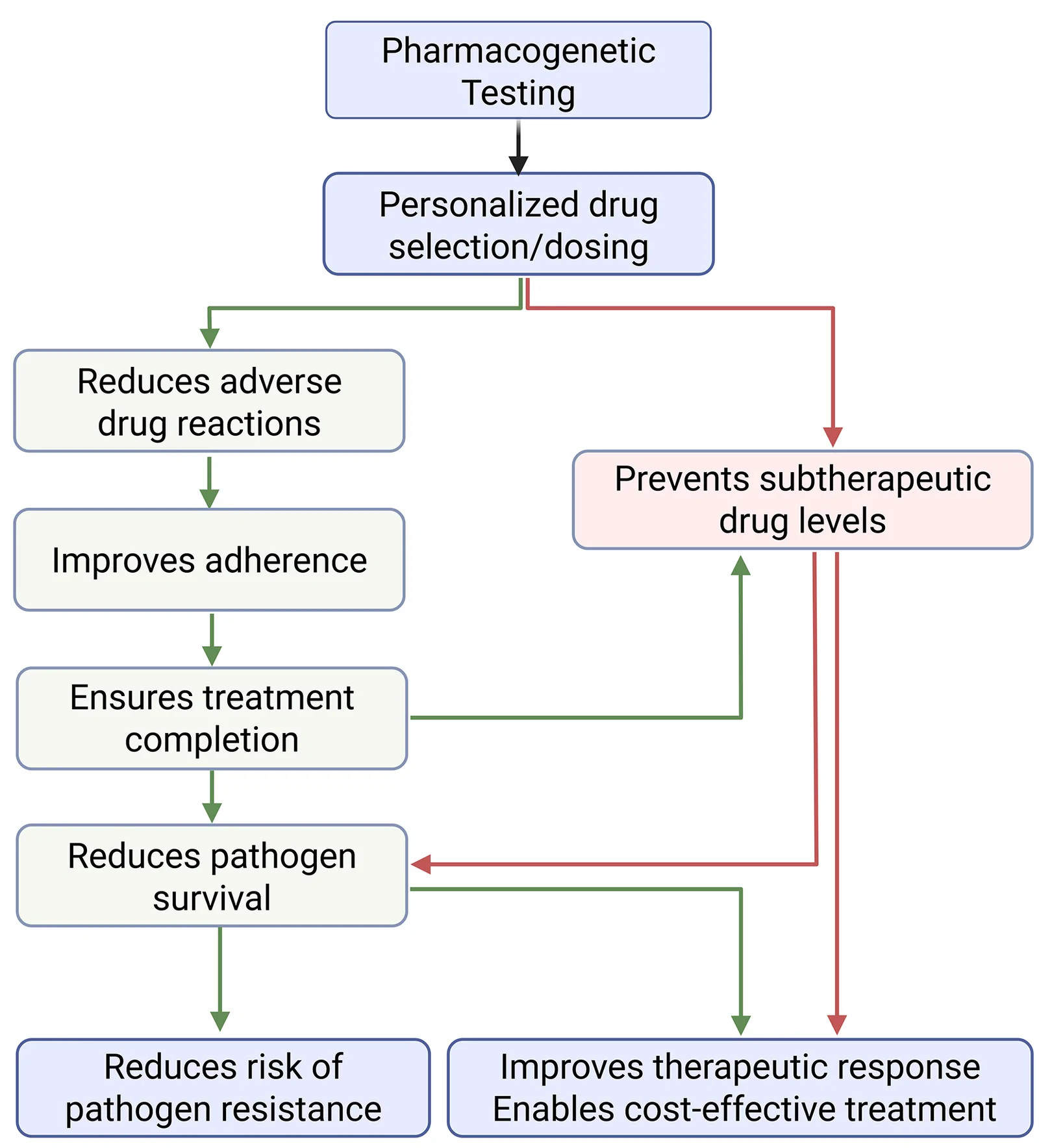
Impact of pharmacogenomic-guided therapy on ID management. This flowchart illustrates how pharmacogenetic testing can optimize anti-infective therapy outcomes and limit pathogen resistance directly (green arrows) or indirectly (red arrows). Created in BioRender. Smatti, M. (2026) https://BioRender.com/wqjqkv7.

The need to optimize anti-infective therapy has never been more urgent, especially in the era of “Bad Bugs, No Drugs” where the threat of multidrug-resistant infections continues to escalate. Although the development of new antimicrobial agents remains challenging, identifying alternative approaches to reduce the emergence of resistant strains requires substantial efforts at multiple levels. Incorporating PGx testing into clinical practice holds significant potential to reduce the misuse and overuse of antimicrobials by enabling personalized dosing, minimizing drug-related toxicity, and preventing subtherapeutic drug exposures, all of which are key components of effective antimicrobial stewardship. PGx-informed clinical interventions can help reduce pathogen resistance both directly, by avoiding suboptimal drug levels, and indirectly, by decreasing ADRs, thereby improving patient adherence and ensuring treatment completion. These factors collectively reduce pathogen survival and lower the risk of mutation and drug resistance, ultimately contributing to more successful and cost-effective treatment outcomes (Fig. 1).

### B Adverse events associated with anti-infective drugs

One of the major concerns that associates with the use of medications, including anti-infective agents, is the possibility of developing ADRs. They are estimated to be the fourth most common cause of death in the world.^19^ These reactions can affect patients’ safety and complicate the treatment course by necessitating additional medical interventions, dose adjustment, or drug discontinuation in some cases. Approximately 5%–10% of all hospitalizations are attributed to ADRs, approximately 80% of which could be predictable and preventable.^20^ ADRs also negatively impact health systems by contributing to high morbidity and mortality, prolonged hospital stays, and increased medical costs.^21^ It is estimated that treating ADRs costs $13,994 in a non–intensive care unit and $19,685 in an intensive care unit setting.^19^ In the United States only, it was reported that ADRs were associated with 1.3% (1.2 million) of hospital stays, whereas in the United Kingdom, 6.5% of all admitted patients experienced ADRs.^22^ These estimates are even higher in children receiving anti-infective agents, possibly because of the high administration of these drugs in the early years of life. Moreover, the level of maturity of physiological systems involved in the absorption, metabolism, distribution, and elimination of drugs could also be a contributing factor.^23^ A recent multicenter study in Brazil involving 1020 children receiving anti-infective drugs found that 152 (15%) experienced ADRs, which were associated with the length of hospital stay and the number of prescribed anti-infective drugs per patient.^24^

ADRs have been traditionally classified into 2 main categories (Table 1). Type A (augmented), which is the most common, results from the drug’s predictable PD and PK properties and is typically dose-dependent. This category includes drug side effects, overdose, and drug–drug interactions. Type B (idiosyncratic) ADRs, on the other hand, are not dose-dependent and are unrelated to the drugs' pharmacological action; therefore, they are unpredictable, uncommon, and often immune-mediated or idiosyncratic.^25^ However, the classification into types A and B was too simplistic to encompass the complexity of these reactions; therefore, 4 additional types (C–F) have been proposed. Type C (chronic) is related to cumulative dose over prolonged treatment; type D (delayed) occurs after a long latency period; type E (end-of-treatment) is associated with treatment discontinuation; and type F (failure of therapy) occurs if the treatment fails to produce the expected therapeutic outcome.^26^ Multiple factors influence the development of ADRs after anti-infective drug use. This could be patient-related (eg, age, sex, comorbidities, polypharmacy, and genetics), drug-related (route of administration, dosage, and formulation), lifestyle-related (diet and smoking), or related to treatment adherence.^22^^,^^27^^,^^28^ Genetic predisposition has been found to contribute to the susceptibility to ADRs to anti-infective drugs. However, most of the current literature specifically describes PGx of type A and B ADRs.^29^ Extensive research has investigated the influence of genetic variations in genes encoding drug-metabolizing enzymes (eg, cytochrome P450 [P450]) or drug transporters (eg, P-glycoprotein [P-gp], encoded by *ABCB1*) and their correlation with type A ADR to anti-infective drugs. The presence of these variations poses a challenge for drug dosing as they correlate strongly with toxicity in patients with no function/reduced function alleles. Variations in immune-related genes, particularly the human leukocyte antigens (HLAs), have received considerable interest given their validated contribution to type B ADRs, such as drug-induced liver injury (DILI) and severe cutaneous adverse reactions (SCARs) associated with antiretroviral therapy (ART).^29^ Notably, our knowledge of the genetic contribution to ADRs of anti-infective agents is increasingly growing, and it is expected to grow even further with the advancement in genome sequencing technologies and the availability of patients’ phenotypic and genotypic data.

**Table 1:** Types of ADRs and relation with anti-infective therapy

Type of ADR	Key Features	Mechanisms	Pharmacogenomic Factors	Examples in Anti-infective Therapy	Clinical Impact	Risk Mitigation Strategies
Type A (augmented)	Dose-dependent Predictable Generally preventable related to drug PK/PD Accounts for ∼80% of ADRs	Exaggeration of pharmacologic effects; drug–drug interactions; overdose	Variation in genes encoding drug-metabolizing enzymes (eg, *CYP2B6* and *CYP3A4*) or drug transport (eg, *ABCB1*)	EFV CNS toxicity in slow *CYP2B6* metabolizers Nephrotoxicity with AGs	Dosing challenges, toxicity management, extended hospital stays, increased costs	Dose adjustments based on genetic screening; close monitoring for toxicity
Type B (idiosyncratic)	Not dose-dependent Unpredictable Immune-mediated or idiosyncratic Less common but severe	HSRs; idiosyncratic immune responses	*HLA* variants (eg, *HLA-B∗*57:01*, HLA-B*∗58:01*,* and *HLA-DRB1*) linked to HSRs	Abacavir hypersensitivity in *HLA-B∗* 57:01 carriers	Severe outcomes (eg, DILI, SCARs), treatment discontinuation	Pretreatment *HLA* screening and discontinuation if reaction occurs; supportive care
Type C (chronic)	Dose and time dependent Related to cumulative dose or long-term use of drugs	Accumulation of drug or metabolites; chronic toxicity	Slow acetylator phenotype (eg, *NAT2* variants) increasing toxicity risk Mitochondrial toxicity and *POLG* (polymerase gamma) and *TK2* (thymidine kinase 2) genes	Isoniazid-induced hepatotoxicity in *NAT2* slow acetylators; zidovudine use and mitochondrial toxicity	Long-term toxicity, treatment discontinuation, higher morbidity, and prolonged needs	Monitoring for long-term effects; withholding and withdrawal of the drug; alternative therapy for prolonged treatments
Type D (delayed)	Manifest after prolonged use or drug discontinuation Often severe and Unpredictable May be immune-mediated	Long latency effects; DNA damage or immune modulation	Variations in *SLCO1B1, NAT2*, *CYP2E1,* and hepatotoxicity	Rifampicin-associated hepatotoxicity manifesting after prolonged use	Late-onset complications, increased healthcare utilization	Prolonged monitoring post-treatment, patient education on symptoms; supportive care
Type E (end-of-treatment)	Occurs after abrupt discontinuation Rebound or withdrawal effects	Loss of drug suppression effects; immune reconstitution	It can— yet not typically—be linked to PGx	Rebound hepatitis after stopping antiviral therapy	Relapse risks, additional treatment costs	Gradual tapering of therapy; reintroduction and gradual withdrawal; patient education on medication adherence; relapse-prevention
Type F (failure of therapy)	Treatment does not achieve the desired effect	Subtherapeutic exposure, Resistance, suboptimal dosing, or interactions	Variations in drug-metabolizing enzymes/transporters lead to resistance	Virologic failure in HIV; HCV reduced drug effectiveness in carriers of *IFNL3* and *IFNL4* gene variants	Disease progression, emergence of resistant strains, increased LOS, higher treatment expenses	Genetic resistance testing; therapy adjustments; prolonged viral load monitoring

LOS, length of stay.

The utilization of PGx markers to identify patients at risk (carriers of specific mutations) and prevent clinically significant ADRs and potential drug interactions can reduce emergency department visits and hospitalizations associated with serious ADRs, thereby lowering overall healthcare costs. For instance, previous reports have shown that *HLA-B*∗57:01 screening is highly cost-effective in preventing abacavir hypersensitivity.^30^ The cost of PGx testing is also anticipated to decline, making it more accessible as more evidence emerges and testing techniques improve, ultimately minimizing the risk of ADRs and improving treatment outcomes. Indeed, vigilant management is required to minimize the burden of ADRs that interfere with effective control of IDs.

## III How genetics modulates responses to anti-infective agents

### A Interference with drug metabolism and transport

**Fig. 2:**
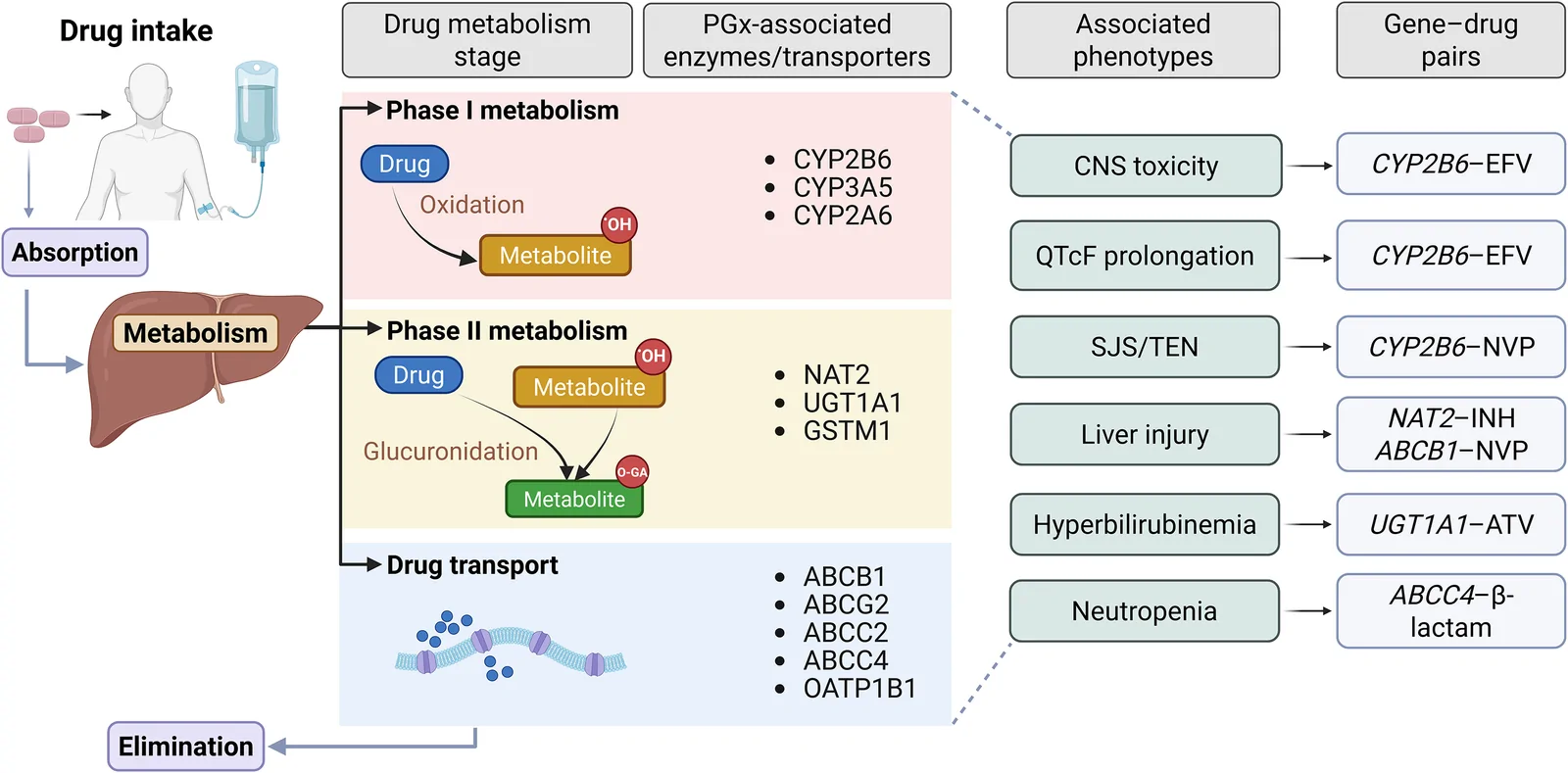
Genetic variations in genes related to drug metabolism and transport. Genetic polymorphisms may influence the response to a drug by altering its PK. Genes encoding phase I or phase II metabolizing enzymes, or transporters could affect drug metabolism, distribution, and associate with adverse events. AZT, 3′-azido-3′-deoxythymidine, zidovudine; INH, isoniazid; QTcF, Fridericia corrected QT. Created in BioRender. Smatti, M. (2026) https://BioRender.com/6iz82ms.

Genetic polymorphisms may influence the response to a drug by altering its PK. In particular, genetic defects in genes encoding enzymes and transporters could modulate the absorption, distribution, metabolism, and clearance of drugs (Fig. 2). This results in interindividual variability in drug exposure, efficacy, and toxicity. It has been previously reported that each individual carries, on average, ∼18,000 variants in 231 pharmacogenes.^31^^,^^32^ Of these, only a small fraction (∼6.5%) resides within exonic or proximal regulatory regions, whereas a subset is novel and predicted to impair protein function based on in silico analyses.^33^ In a subsequent large exome-wide analysis of drug-metabolizing enzyme and transporter genes in the DiscovEHR cohort, the majority of pharmacogenomic variants were rare or ultrarare (minor allele frequency <1%), and approximately 30%–40% of functional variability in drug-metabolizing enzyme and transporter genes was attributable to rare variants, with a higher variant burden observed in individuals of African ancestry.^34^^,^^35^ Recent deep mutational scanning studies have experimentally demonstrated that approximately 20%–40% of coding variants result in reduced or loss-of-function effects, highlighting the functional relevance of rare pharmacogenetic variation even in the absence of clinical annotation.^36^

Polymorphisms in pharmacogenes have been associated with a wide range of drugs, including anti-infective agents. Classical twin studies provided early evidence that a substantial component of interindividual variability in drug metabolism is heritable, thereby motivating later gene discovery efforts in PGx.^37^ For instance, earlier twin investigations demonstrated the key impact of genetic factors on metabolic variability in isoniazid (INH), an anti-TB drug. Monozygotic twins showed lower metabolic variability in INH metabolism compared with dizygotic twins.^38^ Later genomic and mechanistic studies have further deciphered the specific genetic variants contributing to this variability.

The major enzymes involved in phase I hepatic metabolism of all drugs are the P450 family, which metabolize approximately 75% of all medications.^39^ There are at least 57 human P450s, of which 5 are involved in ∼95% of the drug metabolism.^39^ CYP2D6, CYP2C9, and CYP2C19 are responsible for the hepatic metabolism of 25%, 15%, and 10% of all drugs, respectively.^40^ The impact of genetic variation on P450 enzyme activity may result in 5 Clinical Pharmacogenetics Implementation Consortium (CPIC)–recommended phenotype categories: poor, intermediate, and normal (formerly extensive metabolizer), rapid, and ultrarapid metabolizers. Poor metabolizers might experience higher drug exposure and concentration-dependent toxicity when a drug is primarily inactivated by metabolism. For instance, reduced CYP2B6 function (eg, *CYP2B6*∗6/∗6) is associated with higher efavirenz (EFV) plasma concentrations and an increased risk of adverse events.^41^^,^^42^ However, the clinical consequences of metabolizer status are context-dependent, particularly in the case of prodrugs. In such cases, reduced enzyme function can impair conversion to the active form, resulting in subtherapeutic concentrations and potential treatment failure as observed with clopidogrel (platelet inhibitor) in carriers of reduced-function *CYP2C19* variants.^11^ On the other hand, ultrarapid metabolizers may have reduced drug efficacy because of their rapid clearance. In certain cases, a booster is coadministered with the drug to reduce its P450-mediated rapid metabolism in patients carrying extensive metabolizing alleles, such as combining atazanavir (ATV) (anti-HIV drug) with ritonavir.^43^ Beyond rapid clearance, extensive metabolizers can be at risk of drug toxicity as well. For instance, several studies linked EFV (anti-HIV drug) plasma concentration with central nervous system (CNS) toxicity. It has been suggested that extensive metabolizers could be at a higher risk, given that the EFV metabolite (8-hydroxy) produced by CYP2B6 enzyme is neurotoxic.^44^

Genetic variations in genes encoding phase II metabolizing enzymes, which are mainly transferases (glutathione-S-transferases [GSTs], N-acetyl transferases [NAT1/2], and uridine-diphosphate-glucuronosyltransferases [UGTs]), have also been associated with exposure and toxicity of various anti-infective drugs. These enzymes are typically involved in the biotransformation of endogenous compounds, facilitating their excretion. They are also important in the metabolic inactivation of pharmacologically active compounds, reducing drug toxicity and aiding in their elimination.^45^ Carrying *UGT1A1* poor metabolizing, *NAT2* poor acetylating, or glutathione-S-transferase *μ*1 (*GSTM1*) no-function alleles has been associated with several anti-infective agents, including ATV, sulfamethoxazole, and INH.^46^, ^47^, ^48^

Genetic variation can also influence the activity of drug transporters, playing a central role in drug disposition and response. The role of membrane transporters in interindividual variability in drug response has been comprehensively reported and reviewed across different therapeutic areas.^49^, ^50^, ^51^ Drug transporters mediate the translocation of drugs, endogenous molecules, and toxins across membranes through ATP hydrolysis, or ion/concentration gradients.^52^ There are 3 main superfamilies of drug transporters: the ATP-binding cassette (ABC) family, the solute-linked carrier (SLC), and the Solute Carrier Organic anion (SLCO), which was formerly part of the SLC21 subfamily.^53^ The ABC transporter superfamily includes 7 subfamilies (ABCA through ABCG), based on sequence and structural homology. ABC transporters actively transport a variety of ions, organic molecules, peptides, lipids, and drugs, playing a crucial role in drug absorption and excretion. Genetic mutations that alter the expression, function, or efficiency of these transporters have been repeatedly linked to the metabolism and adverse events of anti-infective drugs. For instance, variations in ABC key transporters such as P-gp (*ABCB1*), breast cancer resistance protein (*ABCG2*), and *ABCC2* have been associated with antiretrovirals (ARVs; ATV, EFV, and nevirapine [NVP]) and antibiotics (macrolides and *β*-lactams) as discussed in detail in the following sections.

The SLC superfamily comprises 65 distinct transporter families that mediate the uptake of various molecules. Examples of important SLC transporters in anti-infective drug metabolism include organic anion transporter 3 (OAT3), which is involved in the renal uptake and elimination of drugs such as *β*-lactams (cephalosporins).^54^

The third superfamily, SLCO, also known as the organic anion transporting polypeptide (OATP) superfamily, is divided into 6 families with several functions. Key transporters in this group include OATP1B1, which mediates hepatic uptake of drugs for subsequent conjugation and biliary excretion, an important step in drug elimination. Polymorphic variants of these transporters can cause reduced drug clearance and adverse drug effects.^55^ For example, genetic variations in *SLCO1B1* have been associated with the PKs of macrolide antibiotics such as erythromycin.^56^

Although variation in PK-related genes is crucial, genetic variability in drug targets, including drug receptors or enzymes, can also influence the efficacy of anti-infective agents, as discussed in the following section.

### B Interference with drug targets and signaling pathways

The therapeutic outcomes of anti-infective agents can be shaped by genetic variation in genes related to drug receptors, signaling pathways, and immune system components.^57^ This includes genes encoding cytokines (eg, interferons [IFN] and interleukins [ILs]) and immune receptors such as Toll-like receptors. Unlike genetic variations in PK pathways, PD-related genetic variations affect interactions with molecular targets and their subsequent effects on physiological systems, directly influencing therapeutic outcomes. One of the most robust examples is the variation in IFN lambda 3/4 (*IFNL3/4*) and efficacy of pegylated IFN alfa + ribavirin (PEG-IFN*α*/RBV) in patients with hepatitis C virus (HCV). Carriers of unfavorable variants (eg, rs12979860-CT/TT) exhibit higher baseline expression of IFN-stimulated genes (ISGs). Notably, prolonged expression of ISGs is not always advantageous. Studies have shown that both spontaneous viral clearance and sustained virological response (SVR) to IFN-based therapy are negatively correlated with elevated baseline ISG levels.^58^

Another mechanism by which PGx variants alter PD of anti-infective drugs is through interference with drug-receptor binding. This often occurs because of the presence of pathogen mutations leading to disturbed drug-pathogen binding or overexpression of the target protein. For instance, Y143, Q148, and N155 within the active site of HIV integrase are associated with dolutegravir and raltegravir resistance.^59^ Similarly, Y93H mutation in HCV NS5A protein leads to conformational changes that diminish binding to ledipasvir and confers drug resistance.^60^ On the other hand, a mutation in the promoter region of the *inhA* gene (c-15t) in *Mycobacterium tuberculosis* was found to enhance inhA expression, resulting in titration and resistance of anti-TB drugs.^61^ In contrast to pathogen mutations, host receptor mutations are less commonly associated with drug PD and therapeutic response. For example, mutations in the chemokine receptor 5 contribute to resistance against chemokine receptor 5 antagonists anti-HIV drugs, such as maraviroc.^62^ Collectively, these insights highlight the complex interplay between host and pathogen genetic variation in shaping treatment outcomes.

### C Interference with immune response – human leukocyte antigen genes

On average, human genomes differ by approximately 4.1–5 million polymorphisms from the reference sequence.^63^ However, the HLA complex stands out as an exception, being the most polymorphic region of the human genome with more than 30,000 HLA allelic variants described.^64^ In humans, the HLA (major histocompatibility complex) region includes more than 200 genes, encoding 3 main HLA classes (I, II, and III). Class I HLA molecules are recognized by CD8^+^ T cells, and they consist of *HLA-A*, *HLA-B*, and *HLA-C*, whereas class II HLA, which is recognized by CD4^+^ T cells, includes 6 main genes, *HLA-DPA1*, *HLA-DPB1*, *HLA-DQA1*, *HLA-DQB1*, *HLA-DRA*, and *HLA-DRB1*.^65^ With its key role in antigen presentation and T-cell activation by displaying thousands of peptides to T-cell receptors (TCRs), HLA has been a hallmark genetic marker for predicting susceptibility to immune-mediated diseases, including ADRs. Specific HLA alleles have been associated with a wide range of drug hypersensitivity reactions (HSRs) observed with anti-infective agents. Typically, T-cell responses are under immune regulation that, if disrupted, can lead to different pathologies. Anti-infective drugs or their metabolites can disturb the HLA peptide TCR regulation, triggering an immune response that manifests clinically from mild to serious conditions such as SCARs (including Stevens–Johnson syndrome/toxic epidermal necrolysis [SJS/TEN] and drug reaction with eosinophilia and systemic symptoms), DILI, and hypersensitivity syndrome (HSS).^66^ These severe manifestations are associated with mortality rates of up to 9%, 10%, or 50%, as observed with DILI, drug reaction with eosinophilia and systemic symptoms, and SJS/TEN, respectively.^67^

**Fig. 3:**
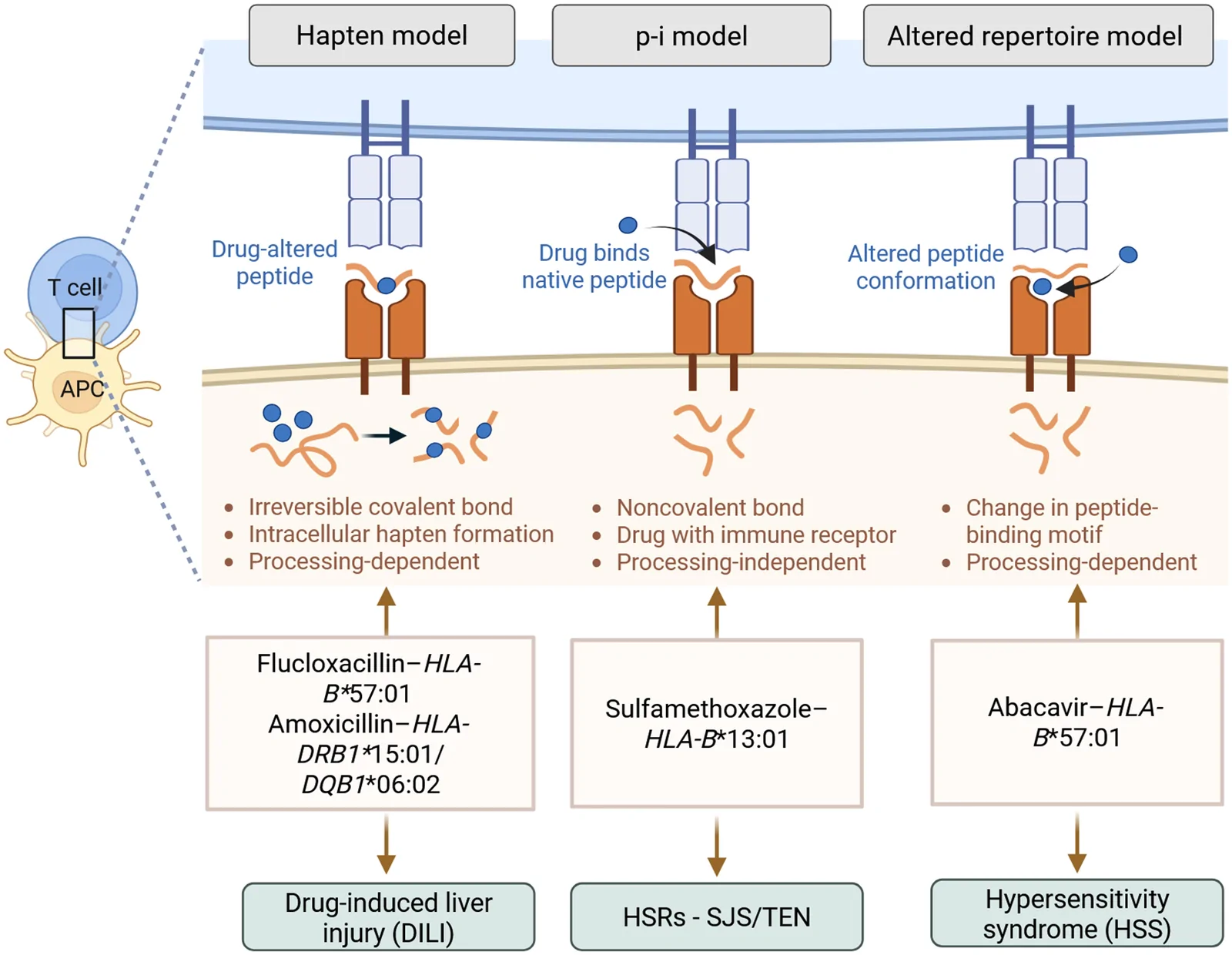
Illustration of mechanistic models in HLA-mediated drug hypersensitivity. Drugs are presented to the TCRs by the HLA molecules through three different mechanisms: The hapten model, the PI (pharmacological interaction with immune receptors) model, and the altered repertoire model. Each model has been proposed of different HLA-drug pairs. APC, antigen-presenting cell. Created in BioRender. Smatti, M. (2026) https://BioRender.com/muscklp.

Drugs or reactive metabolites are presented to the TCRs by the HLA molecules through 3 different mechanisms (Fig. 3). The first is the “hapten model” theory, in which drugs/metabolites bind covalently and irreversibly to an endogenous peptide, creating an antigenic hapten-carrier complex.^65^ In this model, drug-modified peptides and possibly altered peptide sequences are processed before binding the HLA molecule and presented on the surface of antigen-presenting cells, leading to the induction of drug-specific immune responses.^66^ This model is postulated for the interaction between flucloxacillin (*β*-lactam antibiotic) and *HLA-B*∗57:01, which has been associated with DILI.^68^ The second mechanism, the “p-i” model (pharmacological interaction with immune receptors), suggests that the drug binds directly, but reversibly and noncovalently, to the HLA peptide molecule and/or the TCR to induce immune activation. This direct HLA binding has been hypothesized for multiple drug-HLA interactions, including the interaction between sulfamethoxazole and the *HLA-B∗*13:01 molecule.^69^ The third model is the “altered peptide repertoire,” which proposes that the drug or its metabolites occupy a spot in the peptide-binding groove of endogenous HLA molecules, altering the peptide-binding motif and interfering with the peptide specificity of the HLA allele. This alteration can cause T-cells to recognize endogenous peptides as foreign and therefore elicit an immune reaction. The interaction between abacavir and *HLA-B*∗57:01 is the only known example of this model.^65^ Abacavir binds with high specificity to *HLA-B*∗57:01, altering the shape and chemistry of the antigen-binding cleft and, consequently, the repertoire of endogenous peptides that bind the HLA molecule. This activates abacavir-specific T-cells and induces polyclonal CD8 T-cell activation, leading to abacavir hypersensitivity.^70^

Despite the progress made in this field, the study of different pathways of drug-specific T-cell activation has been limited to a small number of drugs. This could be attributed to the limited availability of synthetic, stable, and reactive drug metabolites that are needed for such analyses.^66^ Genetic studies on the association between HLA loci and drug hypersensitivity are continuously increasing, and high-resolution research approaches will be needed to understand the underlying molecular mechanism of HLA-drug interactions that induce T-cell immune-pathogenic phenotypes. Moreover, the inclusion of diverse populations in these studies is essential to uncover population-specific associations and enhance the applicability of the research findings.

Understanding the role of HLA in drug response and further implementing HLA typing in clinical practice would allow for risk avoidance and risk management. For example, *HLA-B*∗57:01 screening is used to prevent abacavir hypersensitivity and also to guide liver function monitoring in patients on flucloxacillin, where the risk of liver injury is rare. Instead of avoiding flucloxacillin entirely, targeted monitoring helps manage this risk. HLA testing also aids diagnostic decisions, such as identifying the cause of DILI when multiple possibilities exist.^66^

### D Interference with redox homeostasis

Many drugs have been putatively linked to an increased susceptibility to acute hemolytic anemia in the presence of glucose-6-phosphate dehydrogenase (G6PD) deficiency.^71^ The mechanism behind this association is that genetic variations in the *G6PD* gene disturb erythrocyte redox homeostasis by limiting the regeneration of NADPH. This diminishes the capacity to maintain glutathione in its reduced, active form that is needed to detoxify cells from endogenous or drug-related oxidants. G6PD catalyzes the reduction of NADP to NADPH during the first oxidative step of the pentose phosphate pathway.^72^ Importantly, in erythrocytes, which lack mitochondria, the pentose phosphate pathway represents the sole source of NADPH, making these cells particularly vulnerable to oxidative stress.^73^ Although drugs are not substrates of G6PD, G6PD deficiency reduces the oxidant-handling capacity of red blood cells, so oxidant exposures can predispose individuals to hemolysis when some drugs are administered. In addition to that, oxidation of hemoglobin iron can result in methemoglobin formation, and methemoglobinemia can accompany hemolysis in affected individuals.^71^ Clinically relevant risk has been reported across several anti-infective classes, most consistently with dapsone (high-risk, actionable), and to a lesser extent with nitrofurantoin (moderate risk, actionable). In contrast, sulfonamides and quinolones (eg, nalidixic acid) are generally considered low-to-no risk.^71^

The *G6PD* gene, which is located on the X chromosome, has more than 150 reported alleles.^71^ *G6PD* alleles have historically been classified by the WHO into classes I–V based on erythrocyte enzyme activity and clinical phenotype. In 2022, a revised classification was proposed, grouping alleles into 4 categories: A (<20% activity, chronic hemolysis), B (<45% activity, acute hemolysis triggered by certain medicines, fava beans, or infection), C (>60% activity, no hemolysis), and U (uncertain clinical significance).^74^ The clinical presentation of acute hemolytic anemia varies widely, in both severity and duration. This is influenced by multiple factors, including drug dose, baseline hemoglobin levels, and interindividual biological variability, notably the underlying *G6PD* genotype. For instance, variants associated with lower residual enzyme activity, such as *G6PD* Mediterranean and *G6PD* Coimbra, are more frequently linked to severe or recurrent hemolysis, whereas variants with higher residual activity, including *G6PD* Seattle and *G6PD* Kalyan-Kerala, tend to be associated with milder clinical courses.^74^ Importantly, *G6PD* genotype alone does not fully predict clinical risk, as X chromosome inactivation in heterozygous females results in wide variability in erythrocyte mosaicism that can outweigh mean enzyme activity differences between variants.^71^ Given the extensive polymorphism of *G6PD* shaped by evolutionary pressure, the WHO recommends the need for continued functional characterization of known and new variants.^74^

## IV Pharmacogenomics of specific classes of antibacterial and antiviral drugs

### A Pharmacogenomics of antiviral drugs

There are approximately 40 antiviral drugs currently being used to treat various viral infections, nearly half of which are anti-HIV drugs.^75^ However, there are no effective treatments for the clinical manifestations of many acute infections, such as Crimean-Congo hemorrhagic fever and Nipah virus encephalitis. Furthermore, most current medications raise concerns regarding effectiveness and/or adverse events. Over the years, viruses have been spreading with more virulence and resistance. It is becoming clear that understanding host factors is key to mitigating the current burden of viral diseases. This underscores the need to leverage the potential of advanced technologies and move beyond traditional pathogen-centered approaches in diagnostics, therapeutics, and drug discovery.

#### 1 Antiretroviral drugs

Following the identification of HIV-1 as the etiological agent of AIDS in 1983,^76^ multiple ARV drugs have been approved for use. This includes azidothymidine (3′-azido-3′-deoxythymidine, zidovudine, or AZT), which was the first licensed ARV drug and represented the cornerstone of ART.^77^ Despite the relatively early development and approval of AZT, HIV-caused mortality remained high until the mid-1990s, when the combined therapy was introduced.^78^

The newer regimen, known as the highly active ART (HAART), the combined ART (cART), or simply ART, involves the coadministration of ≥3 ARV drugs that target different stages of the HIV life cycle so that the propagation of a virus with resistance to a single drug becomes suppressed by the action of the other administered agents. HAART agents include reverse transcriptase inhibitors (eg, zidovudine), protease inhibitors (PIs; eg, lopinavir), integrase strand transfer inhibitors (eg, dolutegravir), fusion inhibitors (eg, enfuvirtide), and chemokine receptor 5 antagonists (eg, maraviroc).^79^ This approach transformed the outcomes of HIV/AIDS drastically, leading to a 50% reduction in AIDS-related deaths in the United States and Europe in a period of only 3 years.^78^ Until now, approximately 50 ARV drugs have been approved by the FDA for HIV treatment.^80^

Although these drugs have made significant progress in HIV treatment and turned a deadly infection into a manageable chronic disease, adverse effects have been reported with all ARV drugs in the earlier era of cART, often leading to therapy switching or discontinuation.^81^ Additional early challenges of cART include the complex dosing, drug resistance, and toxicity, along with the challenges of maintaining long-term adherence. Fortunately, with the extraordinary scientific efforts over the past years to maximize the benefits and minimize the side effects of cART, the effectiveness has improved from 43% in the 1990s to 78% in 2010.^82^ Moreover, the currently used new ARV regimens are associated with fewer serious adverse events than regimens used in the past. Recent studies have shown that long-term use of ART, including nucleoside reverse transcriptase inhibitors (NRTIs), such as the first-line tenofovir disoproxil fumarate, emtricitabine, and EFV, remains central to sustained viral suppression. Moreover, longitudinal data highlight the durability and tolerability of these regimens up to 10 years from initial suppression.^83^

It has been reported that <10% of ART-naive patients enrolled in randomized trials experience treatment-limiting adverse events, although long-term complications may be underestimated because of the short follow-up period and restrictive inclusion and exclusion criteria.^84^ As ART is recommended for all patients with HIV, a major ongoing challenge is to individualize therapy by anticipating and preventing ARV-related toxicities to achieve long-term sustained viral suppression. Although the mechanisms by which ART leads to adverse outcomes are complex and multifactorial, it is now widely accepted that genetic factors influence this process (Table 2).^41^^,^^42^^,^^85^, ^86^, ^87^, ^88^, ^89^, ^90^, ^91^, ^92^, ^93^, ^94^, ^95^, ^96^, ^97^, ^98^, ^99^, ^100^, ^101^, ^102^, ^103^, ^104^, ^105^, ^106^, ^107^, ^108^, ^109^, ^110^, ^111^, ^112^, ^113^, ^114^ Therefore, utilizing drug-specific pharmacogenomic knowledge to understand and predict ARV drug metabolism and transport is crucial for successful personalized anti-HIV therapy.

**Table 2:** Pharmacogenomic variants associated with different phenotypes in response to ARV drugs

ARV	Class	Phenotype	Gene	Genotype	Sample Size	OR	Clinical Relevance	Reference
Abacavir	NRTIs	HSRs	*HLA-B*	*HLA-B∗*57:01	185–1956	19.3–6934	Guidelines supported	85, 86, 87, 88, 89, 90
			*HLA-B + Hsp70-Hom*	*HLA-B∗*57:01 + rs2227956	248	3893	No clinical relevance	87
EFV	NNRTI	↑ Concentration ↓ Clearance	*CYP2B6*	rs3745274 (TT/GT)	79–100	Up to 38.659	Guidelines supported	91,92
		Liver toxicity	*CYP2B6*	∗6/∗6	285	3.31	Guidelines supported	93
		QTcF interval prolongation	*CYP2B6*	∗6/∗6	57 healthy volunteers	NA	Guidelines supported	94
		↑ Concentration	*HNF4A*	rs1884613 (CC)	201	NA	No clinical relevance	95
		↑ Concentration	*CYP2A6*	rs28399433 (AC/CC)	529	NA	No clinical relevance	96
		EFV accumulation in PBMCs	*ABCB1*	rs3842 (CC/CT)	50	NA	No clinical relevance	97
		HSR	*IL-10*	rs1800896 (TT)	63	3.625	No clinical relevance	98
		HSR	*HLA-C*	*HLA-C*∗01:02	75 (38/37)	5.837	No clinical relevance	99
		CNS side effects (risk of abnormal dreams)	*ABCG2*	rs2231142 (GT/TT)	119	5.3	No clinical relevance	100
NVP	NNRTI	SJS/TEN	*CYP2B6*	**rs28399499** (C), rs3745274 (T)	105–489	2.5–4.2	Limited clinical relevance∗	41,42,101
		↑ Concentration ↓ Clearance	*CYP2B6*	∗6/∗6, ∗6/∗18, rs3745274 (GT/TT)	92–112	NA	No clinical relevance	102,103
		↑ plasma ALT	*CYP2B6*	rs58425034 (CC/CG), rs12721646 (CT/TT)	156	NA	No clinical relevance	104
		Liver toxicity	*HLA-B*	*HLA-B*∗35:01	225	5.65	No clinical relevance	105
		Skin rash	*HLA-B*	*HLA-B*∗35	80	3.38	No clinical relevance	106
		SJS/TEN	*HLA-C*	rs5010528	105	5.72	No clinical relevance	107
		Cutaneous adverse events	*HLA-C*	*HLA-C*∗04:01	762	2.51	Limited clinical relevance	105
		Liver toxicity	*HLA-DRB1*	*HLA-DRB1*∗01:01	334	3.02	Limited clinical relevance	105
		↓ Liver toxicity	*ABCB1*	rs1045642 (A)	156	0.42	No clinical relevance	104
		↑ Risk of exanthema	*CCHCR1*	rs746647 (G) rs1265112 (C)	328	4.36	No clinical relevance	108
ATV	Protease inhibitors	↓ Metabolism	*CYP3A5*	Homozygous variants (∗3, ∗6, or ∗7)	31	NA	No clinical relevance	109
		Hyperbilirubinemia	*ABCB1*	rs2032582 (A or T)	129	9.65	No clinical relevance	110
		Hyperbilirubinemia /bilirubin-related ATV discontinuation	*UGT1A1*	∗1/∗28, ∗1/∗37, ∗28/∗28, ∗28/∗37, rs887829 (TT)	39–646	NA	Guidelines supported	111, 112, 113
		↑ Exposure	*NR1I2*	rs2472677 (CC/CT)	Cohort 1 = 47 Cohort 2 = 62	5.1–18	Limited clinical relevance	114

Clinical relevance was defined as: “Guideline-Supported,” indicates the highest level of clinical relevance supported by prescribing guidance from regulatory agencies expert consortia. “Limited Clinical Relevance” denotes replicated associations with moderate-to-high evidence but without current guideline-based prescribing recommendations. “No Clinical Relevance” refers to weak, nonreplicated, or exploratory associations lacking sufficient evidence for clinical implementation.

ALT, alanine aminotransferase; HNF4A, hepatocyte nuclear factor 4*α*; NA, not available; PBMC, peripheral blood mononuclear cell; QTcF, Fridericia corrected QT.

∗ Only the bolded variant(s) in each row are associated with the stated clinical relevance.

##### a Reverse transcriptase inhibitors

###### i Nucleoside/nucleotide reverse transcriptase inhibitors

####### Abacavir

Abacavir is one of the NRTIs that was approved for use in 1998 and is administered in combination with ≥3 other ARV drugs to treat HIV infection.^115^ This typically includes NRTIs, non-NRTIs, PIs, and integrase strand inhibitors.^116^ Because of its flexible dosing options and combination with other ARV drugs in single tablets, abacavir is particularly suited for different cART regimens. Although often well tolerated, the HSRs associated with abacavir can be severe and potentially fatal. In the absence of genetic predisposition, approximately 5%–8% of patients experience HSRs during the first 6 weeks of anti-HIV abacavir treatment.^117^ Fever, shortness of breath, rash, gastrointestinal symptoms, and fatigue are among the symptoms of HSRs. Abacavir-related adverse events require immediate treatment discontinuation, after which symptoms typically subside within 24 hours. However, drug readministration may lead to rapid-onset, life-threatening allergic reactions.^118^ Earlier studies explored the potential risk factors that predispose only a proportion of abacavir recipients to HSRs. White race (odds ratio [OR] = 5.16) and a higher CD8 cell count at initiation of abacavir (OR = 3.74) were found to be significantly associated with the development of HSR.^119^ Another large study analyzed 5332 patients exposed to abacavir and reported that the risk of HSR among Black patients (3% HSR) was lower (OR = 0.59) compared with other ethnic groups.^120^ Further, T-cell infiltrates were reported in positive patch test biopsies.^121^ Together, these findings highlighted the possible role of population genetic variation, particularly in immune-related genes, in abacavir-induced HSR.

The field of PGx in HIV took a leap forward with the subsequent epidemiological and mechanistic research that identified and validated the association of an allele known as *HLA-B*∗57:01 with abacavir HSR. Initially, in 2002, 2 research groups linked *HLA-B*∗57:01 to abacavir HSR in North American and Western Australian patients with HIV.^85^^,^^122^ Hetherington et al^85^ found that *HLA-B*∗57 was present in 39 (46%) of 84 patients compared with 4 (4%) of 113 controls (OR = 23.6, *P* < .0001). A higher sensitivity of the *HLA-B*∗57:01 test in predicting abacavir HSR was reported by Mallal et al.^122^ Out of 18 patients with HSRs, 14 (78%) were *HLA-B*∗57:01 positive, compared with 4 (2%) of the 167 abacavir-tolerant patients (OR = 117, *P* < .0001). Further, the presence of other HLA alleles (*HLA-DR7* and *HLA-DQ3*) along with *HLA-B*∗57:01 had a positive predictive value for HSR of 100% and a negative predictive value of 97%.^122^ Additional fine mapping of the 57.1 ancestral haplotype on an expanded cohort of the Western Australian HIV study (*n* = 248) suggested another susceptibility locus within the 14-kb Hsp70 gene cluster.^87^ In this study, *HLA-B*∗57:01 was present in 94.4% of HSR cases compared with 1.7% of controls (OR = 960; *P* < .00001). In addition to *HLA-B*∗57:01, 94.4% of the patients also had the *Hsp70-Hom* M493T allele; however, only 0.4% of controls had both alleles (OR = 3893; *P* < .00001).^87^ Although these studies provided the first evidence linking HLA and abacavir HSR, their results were limited by the small sample size and the inclusion of predominantly White males, leaving the reproducibility in other ethnicities uncertain.

A subsequent study with an expanded gender and ethnic diversity found that *HLA-B*∗57:01 can predict HSR in White males (OR = 19.3), White females (OR = 36.8), and Hispanics (OR = 30.38) but not in Black individuals (OR = 3.5, positive predictive value = 12%–21%, sensitivity = 8%–16%), suggesting that *HLA-B*∗57:01 alone lacks sufficient predictive value across diverse ethnicities.^86^ Nonetheless, this could be attributed to the small number of Black participants in the study (*n* = 78), especially that the *HLA-B*∗57:01 allele frequency in Black individuals is much lower (0%–0.28%) compared with White (4%–7.9%) as reported in multiple previous studies.^123^, ^124^, ^125^, ^126^, ^127^ The association between *HLA-B*∗57:01 and abacavir HSR was validated in the first double-blind, statistically powered study, “PREDICT-1.”^88^ This large prospective study involved 1956 White patients and stratified the administration of abacavir based on the presence or absence of the *HLA-B*∗57:01 allele. It was found that identifying and excluding *HLA-B*∗57:01 positive patients from abacavir treatment prevented 100% of immunologically confirmed HSRs (0% vs 2.7% in the control group) and reduced clinically diagnosed HSRs (3.4% in the prospective-screening group compared with 7.8% in the control group).^88^ Again, the PREDICT-1 study was predominantly on ethnically White patients. Another group complemented the PREDICT-1 study and retrospectively investigated abacavir HSR in White (*n* = 130) and Black (*n* = 69) patients with HIV.^89^ The study, named “SHAPE,” reported that all patients with immunologically confirmed HSRs carry the *HLA-B*∗57:01 allele (test sensitivity = 100%, OR = 1945 White cohort, OR = 900 Black cohort), and this was consistent across both racial groups. Such high sensitivity of *HLA-B*∗57:01 testing validated its reliability in identifying patients predisposed to abacavir HSR and emphasized its clinical value despite ethnic differences. Nevertheless, *HLA-B*∗57:01 testing recorded 100% negative and 50% positive predictive values, indicating that approximately half of *HLA-B*∗57:01 allele carriers will not develop abacavir HSR. This pinpoints the possible involvement of other genetic and nongenetic factors in HSR development.^128^ Recent proteomic profiling and TCR V*β* chain repertoire analysis found V*β*6 and V*β*24 as potential public TCRs in abacavir-sensitive *HLA-B*∗57:01 carriers. Also, patient-specific factors related to dendritic cell activation and maturation, antigen presentation, IFN, and cytokine regulation contributed to abacavir sensitivity.^129^ Pavlos et al^130^ investigated immune and nonimmune related genes in abacavir HSS and reported multiple other factors. Peptide-trimming allotypes in endoplasmic reticulum aminopeptidase 1 (ERAP1) were more common among patients with abacavir HSS. Also, nonspecific immune activation through the soluble cluster of differentiation antigen 14 could be an independent factor contributing to abacavir HSS susceptibility.^130^

With accumulating evidence of the *HLA-B*∗57:01-abacavir HSR association, the FDA issued a postmarketing communication in 2008 on abacavir (Ziagen) prescribing information.^131^ This included a boxed warning describing the potential risk of serious and fatal HSRs in *HLA-B*∗57:01 positive patients treated with abacavir. The FDA label states that all patients should be screened for *HLA-B*∗57:01 before initiating or reinitiating abacavir therapy and that abacavir is contraindicated in individuals who are *HLA-B*∗57:01 positive. Similarly, the European Medicines Agency (EMA), Health Canada (HCSC), and Swissmedic mandate pretreatment testing. Professional guideline groups, including the CPIC and the Dutch Pharmacogenetics Working Group (DPWG) of the Royal Dutch Association for the Advancement of Pharmacy, strongly recommend *HLA-B*∗57:01 screening before abacavir initiation, with abacavir avoided in positive patients.^117^^,^^132^^,^^133^

The effectiveness of *HLA-B*∗57:01 screening in reducing HSRs continued to be validated across different ethnic groups. A large study in the United States analyzed data from 9619 patients with HIV taking an abacavir-containing regimen for the first time in the pre-*HLA-B*∗57:01 screening period (1999 to mid-2008) or the post-*HLA-B*∗57:01 screening period (mid-2008 to 2016).^134^ High compliance with *HLA-B*∗57:01 screening in the United States was reported, with rates steadily increasing from 43% in 2009 to 84% in 2015. This coincided with a notable decrease in the incidence of HSRs from 1.3% in the prescreening period to 0.8% in 2009 and further to 0.2% in 2015 in the postscreening period.^134^ *HLA-B*∗57:01 screening successfully eliminated HSRs (incidence of 0%) in another study that spanned 7 years (2006–2012) in Portugal.^135^ In the prescreening period, 4% of patients stopped abacavir for suspected HSR, whereas after the implementation of *HLA-B*∗57:01 screening (January 2008), no cases of abacavir HSR were recorded since then.^134^ The ASSURE study also evaluated the utility of *HLA-B*∗57:01 screening in abacavir-naive HIV patients in the United States. It was found that only 1 of 199 *HLA-B*∗57:01-negative subjects (an African American male) randomized to receive abacavir experienced symptoms consistent with suspected abacavir HSR, although not immunologically confirmed.^136^ Recent studies on Middle Eastern populations have also shown a prevalence of *HLA-B*∗57:01 fluctuating between 1.59% (Saudi Arabia), 2.72% (Qatar), 3% (Iran), and 3.3%–3.6% (Turkey).^137^, ^138^, ^139^, ^140^, ^141^ Furthermore, abacavir HSR was very rare in *HLA-B*∗57:01 negative patients, stressing the value of genetic screening in ethnically diverse groups.^137^^,^^139^

The story of *HLA-B*∗57:01 and abacavir-induced HSR has been a powerful model that demonstrated how implementing PGx screening can prevent ADRs associated with anti-infective drugs. It also provided a roadmap for exploring other drug-HLA associations and ADRs by integrating epidemiological, cellular, biochemical, and structural approaches.

###### ii Non-nucleoside/nucleotide reverse transcriptase inhibitors

####### Efavirenz

EFV is a nonnucleoside reverse transcriptase inhibitor (NNRTI) that has been widely used since its FDA approval in 1998. In 2016, the WHO also recommended using EFV with tenofovir disoproxil fumarate and lamivudine/emtricitabine as the preferred first-line ARV regimen for treating and preventing HIV.^142^ Currently, EFV is still a first-line regimen as an alternative to dolutegravir, which was recommended for use in combination with an NRTI backbone as per the recent WHO guidelines.^143^ EFV is known for its clinical efficacy and favorable pharmacological properties and is generally well tolerated. Nonetheless, serious adverse events have been associated with EFV use, including CNS symptoms, hepatotoxicity, and corrected QT (QTc) interval prolongation.^94^ Multiple studies have reported a high EFV discontinuation rate because of intolerable side effects, ranging from 15.6% to 35.8%.^144^

Among the EFV-containing HAART regimens-related side effects, neuropsychiatric events are particularly prevalent, as observed in approximately 50% of patients.^145^ Although these side effects are usually transient and mild to moderate in severity, severe late-onset EFV toxicity, causing ataxia and encephalopathy, has been reported in multiple studies since 2016.^146^, ^147^, ^148^, ^149^ EFV adverse events generally, and neurologic and neuropsychiatric events particularly, (such as neurocognitive impairment, depression, and suicidality), have been associated with elevated plasma EFV exposure that is accompanied by slow EFV metabolism.^150^ The ratio of patients experiencing CNS side effects was directly proportional to EFV plasma concentration. CNS side effects were detected in 24%, 9%, and 0% of patients with plasma EFV >4, 4–1, and <1 *μ*g/mL, respectively.^151^

Hepatotoxicity represents another serious, though less frequent, adverse effect of EFV. Serum aminotransferase elevations exceeding 5 times the upper limit of normal occur in approximately 1%–8% of patients on EFV.^152^ Moreover, grade 3 or 4 hepatotoxicity was observed in 4.4%–8% of patients on EFV in multiple clinical trials.^153^ Importantly, although EFV is a potent ARV drug against HIV-1, it has a narrow therapeutic index and exhibits a wide interindividual variability in its kinetics and dynamics, highlighting the importance of individualized dosing and therapeutic drug monitoring.^154^

The variability in response to EFV is partially attributed to genetic variations in the *CYP2B6* gene, which encodes the primary enzyme responsible for its metabolism.^155^ *CYP2B6* is a significant element of the P450 system and is the only gene in the human *CYP2B* subfamily that encodes a functional enzyme. CYP2B6 accounts for ∼2%–5% of the total hepatic P450 content in human liver tissues, despite large interindividual differences.^156^ Notably, although the relative contribution of CYP2B6 to total hepatic P450 content is minor, it is involved in the metabolism of approximately 8%–13% of all drugs in the market.^157^, ^158^, ^159^ EFV is metabolized to 8-hydroxy-EFV (8-OH EFV), a major primary oxygenated metabolite formed by CYP2B6. EFV is also metabolized to a lesser extent to the major primary 7-OH EFV and the minor secondary 8,14-OH EFV by CYP2A6 and CYP2B6, respectively.^160^, ^161^, ^162^, ^163^, ^164^ Importantly, these metabolites lack any significant pharmacological activity against HIV-1 and have been linked to clinical neurotoxicity, leading to dendritic spine damage in vitro.^150^^,^^165^ Of note, 8-OH EFV was particularly found to produce at least an order of magnitude more damage to neurons than the parent EFV and 7-OH EFV.^165^

Furthermore, EFV is glucuronidated by the UGT-2B7 to form EFV-N-glucuronide.^166^ Given that S-8-hydroxy-EFV is the major oxygenated metabolite formed by the CYP2B6 enzyme, a decrease in the levels of oxygenated metabolites along with the elevation in the levels of EFV-N-glucuronide was reported in *CYP2B6* loss-of-function alleles.^167^^,^^168^ EFV intake enhances CYP2B6 expression via activating the constitutive androstane receptor. This, consequently, enhances EFV metabolism in an autoinduction process. This autoinduction is influenced by *CYP2B6* genetic structure and, therefore, demonstrates interindividual variability.

*CYP2B6* is genetically highly polymorphic, with 49 known variant alleles and multiple suballeles (as of January 2026) listed in the Pharmacogene Variation Consortium (PharmVar) database.^169^ These alleles are named ∗1 (wild type) to ∗50 (*CYP2B6*∗16 was consolidated under the ∗18) according to the star nomenclature system that denotes the combinatorial effect on enzyme activity.^156^ The latest PharmVar GeneFocus review summarizes the core and historically validated *CYP2B6* alleles,^156^ whereas more recent studies identified additional novel star alleles that have been incorporated into the PharmVar database, including ∗39–∗49 and the recently assigned ∗50 allele.^170^^,^^171^ By definition, *CYP2B6* star alleles have amino acid sequence changes or exhibit a functional impact on the enzyme activity.

The combination of *CYP2B6* inherited alleles defines an individual’s diplotype, which, in turn, can contribute to 5 possible metabolizer phenotypes according to the CPIC: ultrarapid, rapid, normal, intermediate, and poor metabolizers.^154^ The first identified and most studied *CYP2B6* variant is rs3745274 (G>T), which is a loss-of-function variant in *CYP2B6*∗6 (in combination with rs2279343 [A>G]) or alone in *CYP2B6*∗9.^91^ Homozygous (TT) and heterozygous carriers (GT) of rs3745274 demonstrated a 50% and 25% reduced EFV clearance, respectively.^150^ In addition to *CYP2B6∗*6 and ∗9, ∗7, ∗8, ∗12, ∗13, ∗18–∗20, ∗24, ∗26, ∗28–∗30, and ∗34–∗38 haplotypes were also linked to a decreased or “no function” CYP2B6 enzyme activity. On the other hand, evidence based on a small number of patients showed modestly lower EFV plasma exposure in *CYP2B6∗*4 and ∗22 carriers, indicating that these alleles increase enzymatic activity.^154^ Although *CYP2B6∗*1 (wild-type), ∗2, ∗5, ∗17, ∗31, and ∗32 have a normal function, the remaining star alleles were designated unknown or uncertain function, requiring further studies. Importantly, the frequency and clinical impact of *CYP2B6* polymorphisms have been explored and reproduced across different ethnicities, providing the basis for current clinical recommendations.^154^

Multiple *CYP2B6* genotypes have been associated with EFV metabolism and toxicity (Table 2). For instance, *CYP2B6*∗9 (TT/GT genotypes) has been linked to reduced clearance and increased concentration.^91^^,^^92^ Moreover, *CYP2B6*∗6 has been reported as a putative genetic marker for vulnerability to EFV-induced liver injury in patients with HIV.^93^ Importantly, if EFV-related toxicities are not recognized and managed early, they can lead to serious complications or even death.^146^ EFV dose adjustment based on the patient’s genotype may help prevent drug-related toxicity, unresponsiveness, and virological rebound.

The current CPIC guidelines recommend EFV dose adjustment according to the *CYP2B6* genotype. For ultrarapid metabolizers, when an individual carries 2 alleles associated with increased function, such as ∗4/∗4, ∗22/∗22, or ∗4/∗22, it is strongly recommended to initiate EFV with standard dosing (600 mg/d). This is also recommended for rapid metabolizers who carry 1 normal allele (eg, ∗1) and 1 increased function allele (∗4 or ∗22). On the other hand, intermediate metabolizers (carrying 1 normal function allele and 1 decreased function allele or 1 normal function allele and 1 no-function allele, or 1 increased function allele and 1 decreased function allele, or 1 increased function allele and 1 no-function allele) are recommended to receive a decreased EFV dose (400 mg/d). This dose or even a lesser dose of 200 mg/d is recommended for poor metabolizers with 2 decreased function alleles (eg, ∗6/∗6), 2 no function alleles (eg, ∗18/∗18), or 1 decreased and 1 no function allele (eg, ∗6/∗18).^154^

Studies are now largely consistent in demonstrating an increased risk of dysrhythmia and sudden cardiac arrest among patients with HIV as compared with healthy individuals.^172^ In the United States, sudden cardiac death accounts for 5%–15% of cardiovascular deaths associated with HIV-AIDS, which is roughly 4.5-fold higher than expected.^173^ ART, including EFV, has been linked to an increased risk of several cardiovascular abnormalities such as QT-prolongation and Torsade de Pointes arrhythmia, which are strongly associated with sudden cardiac death.^174^^,^^175^ EFV has been associated with an increased risk of total dysrhythmia (OR = 2.90). Moreover, patients on EFV regimens had a significantly higher incidence of high premature ventricular contractions, low heart rate variability, and prolonged QTc than controls.^176^ In particular, *CYP2B6*∗6/∗6 carriers receiving EFV therapy demonstrated a gene-dose effect and exceeded the FDA criteria for the rate-Fridericia corrected QT interval prolongation.^94^ This suggests that homozygous carriers of the *CYP2B6*∗6 allele may be at a higher risk of EFV-induced Fridericia QTc interval prolongation. Notably, the FDA-approved EFV (SUSTIVA) drug label indicates that patients carrying the *CYP2B6*∗6/∗6 genotype (poor metabolizers) have higher EFV concentrations than those carrying the ∗1/∗1 genotype. It also states a positive correlation between high EFV concentration and QTc prolongation.^177^ The FDA and other regulatory agencies, including the EMA and HCSC, classify the *CYP2B6* genotype information as informative PGx, whereas Swissmedic and Pharmaceuticals and Medical Devices Agency (PMDA) consider it actionable PGx, reflecting regional differences in implementation and clinical guidance.

Despite the evident significance of adjusting the EFV dose based on patients’ *CYP2B6* haplotype, genetic testing is unlikely to be widely implemented, especially in resource-limited areas. This includes the African region, which remains most severely affected. Nearly 1 in every 30 adults (3.4%) is living with HIV, accounting for more than two-thirds of the people living with HIV in the world (as of July 2024).^178^ Importantly, this coincides with the high frequency of multiple poor metabolizing alleles in African individuals compared with other ethnic groups. For instance, the *CYP2B6*∗18 allele, which is associated with no enzymatic function, occurs almost exclusively in people of African heritage (frequency 7.1%, compared with <0.5% in other populations). A clinical study found that daily EFV 400 mg (EFV400) was virologically noninferior to the 600 mg dose (EFV600). HIV suppression was comparable between doses despite significantly lower EFV400 exposure.^44^ This indicates that reducing the EFV dose to 400 mg could be economically viable and advantageous in reducing adverse events, especially where genetic testing is not feasible.

Besides *CYP2B6* gene polymorphisms, genetic variants in other genes have also been associated with EFV outcomes in patients with HIV. This includes other *P450* genes (*CYP2A6* and *CYP3A5*), *HLA*-C, *ABCB1*, hepatocyte nuclear factor 4*α*, *IL-10*, nuclear receptor subfamily 1 group I (*NR1I*2 and *NR1I*3), and UDP glucuronosyltransferase family 2 member B7 (*UGT2B7*) genes.^96^, ^97^, ^98^, ^99^^,^^179^, ^180^, ^181^ Although these variants were significantly associated with EFV clinical phenotypes (*P* value ranges from <.05 to <.0001), none scored a high or moderate clinical annotation level as per the PharmGKB scoring system. Moreover, some of these results were inconsistent among different studies. Overall, only a low or preliminary level of evidence supports these associations, and no routine clinical implementation is recommended at present.

####### Nevirapine

NVP is another NNRTI that was approved by the FDA in 1996. This ARV is often used to prevent mother-to-child HIV transmission. Typically, ART should be started immediately for pregnant and breastfeeding mothers diagnosed with HIV to reduce viral load and prevent vertical transmission. Infants born to infected mothers should also be given daily prophylaxis containing AZT and NVP or NVP alone.^143^ Despite its efficacy, NVP has been associated with several side effects. NVP-associated hypersensitivity occurs in approximately 6% of patients, leading to therapy discontinuation.^182^ NVP toxicities can manifest in several forms and may be severe, as observed with SJS and its severe form, TEN. Notably, the incidence of SJS in children was recently estimated at 7.1%, indicating a significantly higher risk of NVP in children living with HIV than previously expected.^183^ Other manifestations of NVP toxicities include hepatotoxicity that can be severe in some cases (1.3%–12%) and, in rare cases, fatal (eg, fatal hepatic necrosis). Of note, in 2000, the FDA issued a boxed warning about the serious risk of NVP-induced hepatotoxicity.^184^ Compared with other ARV drugs such as EFV, NVP has been associated with a higher incidence of severe hepatotoxicity and more frequent treatment discontinuation.^153^ Females and patients with increased CD4^+^ cell counts are at higher risk of liver toxicity.^185^ The mechanism by which NVP induces liver injury has been comprehensively reviewed by Benedicto et al.^186^

NVP is metabolized by CYP2B6 into 3- and 8-hydroxynevirapine and by CYP3A4 into 2- and 12-hydroxynevirapine. CYP3A5, CYP2C9, and CYP2D6 are also involved in NVP metabolism, but to a lesser extent.^187^ NVP monooxygenated metabolites can be further glucuronidated in subsequent phase II metabolism by UGT enzymes, and this accounts for 80% of the elimination of NVP.^188^ Genetic polymorphisms in the *CYP2B6* gene, particularly the poor metabolizing ∗6/∗6 and ∗6/∗18 haplotypes, have been strongly associated with increased NVP plasma concentration. Carriers of these haplotypes exhibited a 24% increase in NVP exposure compared with the normal ∗1/∗1 haplotype (*P* value < .01).^102^ In a meta-analysis including the data from 634 patients, Yoon et al,^189^ reported that carriers of the rs3745274 *CYP2B6* 516TT genotype (which is found solely in *CYP2B6*∗9 and with rs2279343 in ∗6 alleles) had 2.18 *μ*g/mL higher NVP concentrations than those observed in individuals with the GG or GT genotypes. *CYP2B6* polymorphisms are not only associated with NVP plasma concentration but also with the clinical manifestation of NVP toxicity. Specifically, *CYP2B6*∗9 and ∗18 have been strongly associated with NVP-induced SJS/TEN, as reported by multiple groups.^41^^,^^42^

Several studies highlighted a possible role of P-gp (encoded by the ATP-binding cassette transporter gene) in NVP PD; however, this association remains incompletely characterized. In particular, the ATP binding cassette subfamily B member 1 (*ABCB1*, also called *MDR1*) c.3435C>T variant was significantly associated with NVP-induced hepatotoxicity risk, where the T allele demonstrated a protective effect.^190^ Carriers of the TT genotype exhibited a significant rise in the CD4-cell count compared with CT allele carriers (257 and 121 cells/*μ*L, respectively, *P* = .0048). Other groups replicated this finding and validated the protective role of the *ABCB1* T allele (OR = 0.30–0.42, *P* value < .05).^104^^,^^191^

Genome-wide association studies (GWASs) identified genomic variants associated with NVP-induced toxicity. Two single nucleotide polymorphisms (SNPs; rs1265112 and rs746647) within the coiled-coil *α*–helical rod protein 1 (*CCHCR1*) gene were found to be associated with NVP-induced rash in the Thai population.^108^ Of note, the *CCHCRI* gene is located 110 kb telomeric from the *HLA-C* locus and 210 kb from the *HLA-B* locus, and the linkage disequilibrium (LD) between *HLA-B*∗35:05 and *CCHCR1* could explain this finding as multiple HLA alleles have been linked to NVP toxicity and HSRs (reviewed in Yang et al^192^). Briefly, *HLA-DRB1*∗01:01 was initially reported to be associated with susceptibility to NVP hypersensitivity in Western Australian patients. It was later found that this specific allele is more associated with hepatotoxicity among Caucasians. *HLA-Cw*∗08 and *HLA-Cw*∗04 alleles were also linked to hepatotoxicity and HSRs in multiple Asian and Sardinian populations. Additionally, *HLA-B*∗35:05 was reported as a strong predictor for NVP-induced skin-associated ADRs in Thai patients, whereas *HLA-Cw*∗04 was associated with NVP cutaneous ADRs in multiple ethnic groups. In addition to that, the risk of SJS/TEN was associated with *HLA-C*∗04:01 among African patients.^192^ rs5010528 (an *HLA-C* gene variant) is a strong proxy for *HLA-C*∗04:01 carriage and was also linked to SJS/TEN susceptibility from Mozambique.^107^ Further bioinformatics analysis showed that rs5010528 is differentially expressed in skin and appears to differ across populations. African and American populations appear to have the highest allele frequency of rs5010528 (∼24%) compared with other ethnicities (6%–16%).^193^ Together, these findings highlight the potential impact of HLA variability on the susceptibility to NVP-induced adverse events and underscore the necessity for additional studies involving mixed and larger populations.

Among all the identified gene variations related to NVP toxicity, only the *CYP2B6*, *HLA-DRB1*, and *HLA-C*∗04:01 variants have reached moderate evidence in relation to NVP toxicity (evidence level 2A, 2B, and 2B, respectively) according to the PharmGKB scoring system. This indicates a “likely causation” between *CYP2B6* and NVP and “moderate evidence” supporting *HLA-C* and *HLA-DRB1* association with NVP toxicity. However, further validation is needed. Other gene–drug pairs have been assigned low to no evidence of association according to both PharmGKB and CPIC frameworks. At present, no pharmacogenetic guidelines mandate or recommend routine testing for these markers in NVP therapy.

##### b Protease inhibitors

###### i Atazanavir

ATV was FDA-approved in 2003, and it belongs to the PI class of HIV-1 ARTs. ATV is typically used as part of cART, commonly coadministered with a PK enhancer (boosting agent), particularly ritonavir or cobicistat, along with 2 NRTIs.^43^ ATV is used for treating HIV-1 infection in both treatment-naive and treatment-experienced patients. It is also occasionally used off-label for HIV postexposure prophylaxis when exposure is suspected, such as needlestick incidents.^194^ With fewer metabolic side effects compared with other PIs and a once-daily dose, ATV, especially when boosted, is considered a favorable anti-HIV treatment for certain clinical situations, including pregnant women, patients with cardiovascular diseases, and patients with diabetes.^195^ ATV causes fewer metabolic complications given its lack of interference with key pathways in lipid and glucose metabolism. Earlier studies found that compared with other PIs, ATV does not inhibit 20S proteasome activity, adipogenesis, or insulin-stimulated glucose uptake into cells.^196^ More recently, it was found that ATV has no detrimental effects on adipose tissue-derived stem cells, but may impair adipocyte differentiation by inducing endoplasmic reticulum stress and apoptosis, potentially contributing to ATV-induced lipodystrophy.^197^

Among the commonly observed adverse events during ATV therapy are hyperbilirubinemia (35%–49% in adults and 16% in children), hypercholesterolemia (6%–25%), hyperamylasemia (14%–33%), and jaundice (5%–9% in adults and 13%–15% in children). In addition, nephrolithiasis, cholelithiasis, cholecystitis, pancreatitis, hyperlipidemia, and SJS have been reported as potential side effects.^194^

ATV is mainly metabolized by CYP3A4 and CYP3A5.^195^ Ritonavir, also a PI, is often administered with ATV to block the CYP3A-mediated metabolism of ATV. This acts as a booster to enhance ATV effectiveness and improve its PK profile.^43^ Several studies demonstrated the effect of *CYP3A5* genotypes on the PK of ATV, particularly in the absence of ritonavir boosting. When ATV is administered unboosted, patients carrying at least 1 functional *CYP3A5*∗1 allele (CYP3A5 expressers) exhibit higher oral clearance (CL/F) compared to those carrying no functional *CYP3A5* alleles (CYP3A5 nonexpressers), with a reported increase of 28%.^109^^,^^198^ In contrast, individuals with any combination of 2 nonfunctional alleles (∗3, ∗6, or ∗7) tend to metabolize ATV more slowly.^109^ In clinical practice, *CYP3A5* genotyping is not currently used to guide routine ATV prescribing, and no formal genotype-based dosing recommendations exist. However, knowing the patient’s *CYP3A5* genotype may help contextualize interindividual differences in ATV exposure in selected situations, particularly when unboosted regimens are used.

In ritonavir-boosted regimens, the impact of the *CYP3A5* genotype is substantially attenuated but not completely abolished. In a population PK–pharmacogenetic study, CYP3A5 nonexpressers demonstrated a 7.1% lower oral clearance of ATV than expressers, and simulations suggested that reduced ATV/ritonavir doses could still achieve therapeutic trough concentrations in most patients.^199^ Although the clinical implications of these effects do not currently warrant genotype-guided dose adjustment, they highlight the role of pharmacogenetic factors in explaining interindividual variability in ATV exposure.

Given that ATV is considered a substrate of P-gp (*ABCB1* gene), the association between *ABCB1* gene variants and ATV metabolism has been explored in various studies. Multiple polymorphisms in *ABCB1* and the pregnane X receptor (*PXR*; *NR1I2*), which regulates the expression of *CYP3A4* and *ABCB1*, have been reported.^109^^,^^114^^,^^200^^,^^201^ Nevertheless, most of these associations have been limited by small sample sizes, and according to PharmGKB, these variants score low to unsupported levels of evidence.

ATV inhibits the hepatic UGT1A1 enzyme, which is essential for the glucuronidation and elimination of bilirubin. However, a common side effect of ATV is the accumulation of unconjugated bilirubin (hyperbilirubinemia) in tissues and blood, which can, in some instances, compromise treatment adherence.^46^ Reduced UGT1A1 activity through genetic variation increases the risk of bilirubin-related ATV discontinuation. To date, there are more than 191 unique variants and 113 reported star alleles in the *UGT1A1* gene.^202^^,^^203^ The most frequent genetic variant is the di-nucleotide (TA) repeats positioned in a TATAA consensus element in the gene promoter, which varies from 5 to 8 repeats. The wild-type *UGT1A1* (∗1 allele) contains 6 TA repeats [A(TA)_6_TAA], or 7 TA repeats if the additional TA listed in the flanking sequence as “TAA” was added to the count. The higher the number of TA repeats in the promoter region, the lower the UGT1A1 enzyme activity. For instance, *UGT1A1*∗28 (TA)_7_ and ∗37 (TA)_8_ alleles exhibit decreased enzyme activity. These alleles are included in the CPIC guideline of ATV prescribing, in addition to other decreased function alleles (*UGT1A1*∗6, ∗27, ∗37, ∗80+∗28, and ∗80+∗37), and normal function alleles (∗1 and ∗36).^46^ The current classification of phenotypes associated with *UGT1A1* alleles defines individuals as normal metabolizers (eg, ∗1/1∗, ∗1/∗36, ∗36/∗36, or homozygosity for rs887829 C/C), intermediate metabolizers [carrying 1 decreased function allele (eg, ∗1/∗6 or ∗1/∗28)], or poor metabolizers [carrying 2 decreased function alleles (eg, ∗6/∗6, ∗28/∗28, ∗37/∗37)]. The distribution of the *UGT1A1* alleles varies markedly between ancestral groups, with the wild-type (∗1) and the decreased function (∗28) alleles being the most frequent in almost all populations. *UGT1A1*∗80, which is in very high LD with ∗28 and ∗37, is also frequent, especially among African American, European, and Latino groups. On the other hand, ∗6, ∗36, and ∗37 are rare or absent regardless of the geographical region.^46^

The *UGT1A1*-ATV association represents one of the most extensively evaluated pharmacogenetic relationships in ART and has been assigned a CPIC level A/Final designation. CPIC and DPWG guidelines indicate that *UGT1A1* genotype information, when available before therapy, can help identify individuals at increased risk of ATV-associated hyperbilirubinemia and support consideration of alternative ART where clinically appropriate.^46^ Specifically, CPIC recommends considering alternative therapy in patients with *UGT1A1* poor metabolizer genotypes, particularly when jaundice would be clinically problematic, whereas routine avoidance in intermediate metabolizers is not required. Patients with high-risk genotypes should be counseled regarding the increased likelihood of bilirubin-related treatment discontinuation. Despite CPIC and DPWG guidance, pre-emptive *UGT1A1* testing is not currently mandated by major regulatory agencies.

#### 2 Hepatitis C drugs

Approximately 50 million people are living with HCV infection globally, with about one million new cases occurring every year.^204^ This infection claimed the lives of almost a quarter of a million people in 2022, mainly as a result of cirrhosis and hepatocellular carcinoma. Only 15%–45% of individuals infected with HCV can clear the virus spontaneously, whereas most patients (60%–80%) experience a chronic disease.^204^ HCV has a high genetic variability, with at least 8 major genotypes (GT1–GT8) with 93 subtypes and notable geographic variation in their distribution.^205^ HCV genotype 1 is the most prevalent worldwide followed by genotype 3, genotype 2 is found in West Africa; genotype 4 dominantly in Middle East and North Africa region, genotype 5 in South Africa, genotype 6 in Southeast Asia.^206^ The recently identified genotypes have a narrower distribution, with genotype 7 being reported the Democratic Republic of Congo and Uganda, and genotype 8 reported in patients from India.^207^^,^^208^ Importantly, HCV diversity has consequence with regard to treatment outcomes, especially when IFN-based therapies were used. Patients with genotype 2 or 3 achieve a SVR rate of 80%, whereas for those with genotype 1, the SVR rate decreases to approximately 40%.^209^ Nonetheless, the third-generation IFN-free direct-acting antivirals (DAAs) are considered pangenotypic and are substantially efficacious against a broad range of genotypes.

HCV treatment focuses on achieving SVR, which indicates the absence of HCV viremia for 24 weeks after cessation of antiviral therapy.^210^ With the use of combined therapy of PEG-IFN*α*/RBV, a marked increase in the SVR from <10% in the 1990s to >50% has been observed.^211^ This combined treatment represented the standard HCV therapy until 2011, when new DAAs with higher efficacy, safety, and tolerability were approved. Although PEG-IFN/RBV is no longer prescribed as a first-line HCV treatment, it is still used in certain conditions.^212^ Treatment with PEG-IFN*α*/RBV results in different SVR rates depending on virus factors (virus genotype) and host factors (genetic variation in genes involved in IFN pathways). Genetic variants around the IFN lambda 3/4 gene locus were repeatedly associated with spontaneous clearance of HCV and PEG-IFN/RBV treatment responsiveness (Table 3). In fact, this association represents one of the most robust and frequently validated GWAS findings in the field.

**Table 3:** Pharmacogenomic variants associated with different phenotypes in response to HCV drugs

Drug Category	Gene/Region	Associated Drugs	Key Variants	Effect	Clinical Relevance
Older (IFN-based) regimens	*IFNL3/IFNL4*	PEG-IFN + ribavirin ± simeprevir/telaprevir	**rs12979860** (CC), **rs11322783** (TT/TT), **rs8099917** (TT), rs12980275 (AA), rs28416813 (CC), rs4803219 (CC), rs7248668 (GG), rs8105790 (TT), **rs11881222** (AA), rs117648444 (AA, AG)	↑ SVR	Guideline-supported (historical) Limited current relevance∗
		PEG-IFN + ribavirin	rs4803217 (AA)	↓ Treatment response	No clinical relevance
	*ITPA*	PEG-IFN + ribavirin	rs1127354 (CC), rs7270101 (AA), rs6051702 (AA)	Anemia	Limited clinical relevance
	*HLA-B*	PEG-IFN + ribavirin	*HLA-B*∗38:01	No response to treatment	No clinical relevance
			*HLA-B*∗44:02	↑ SVR	No clinical relevance
	*HLA-C*	PEG-IFN + ribavirin	*HLA-C*∗01:02,03:02, 07:01, 08:01, 12:02,14:02, 16:01	↑ Treatment response	No clinical relevance
			*HLA-C*∗02:02, 04:01, 05:01, 06:02, 15:02, 17:01	↓ Treatment response	No clinical relevance
	*IL-18*	PEG-IFN + ribavirin	rs187238 (GG), rs1946518 (TT)	↑ Treatment response	No clinical relevance
	*IL-6*	PEG-IFN + ribavirin	rs1800795 (CC)	Depression risk	No clinical relevance
	*VDR*	PEG-IFN + ribavirin	rs1544410 (TT)	Anemia	No clinical relevance
			rs2228570 (AA, AG)	↑ SVR	No clinical relevance
	*CYP2R1/SCARB1/OASL/MICB*	PEG-IFN + ribavirin	rs10741657 (AA, AG), rs10846744 (CC, CG), rs12819210 (TT), rs3828913 (CC)	↑ SVR	No clinical relevance
	*EGFR*	PEG-IFN + ribavirin	rs11506105 (AA)	↑ SVR, RVR, cEVR	No clinical relevance
	*CYP24A1*	Telaprevir	rs2585428 (CC, CT)	↓ Drug levels	No clinical relevance
	*SLC28A2/SLC29A1*	PEG-IFN + ribavirin ± telaprevir/boceprevir	rs11854484 (TT), rs760370 (GG)	Anemia, ↑ RVR	No clinical relevance
Newer (IFN-free) DAA regimens	*IFNL3/IFNL4*	DAAs (eg, sofosbuvir, ledipasvir, and velpatasvir)	rs11322783 (TT/TT), rs8099917 (TT)	↑ SVR	No clinical relevance
			rs12979860 (TT, CT)	↑ Relapse	No clinical relevance

Clinical relevance was defined as: “Guideline-Supported,” indicates the highest level of clinical relevance supported by prescribing guidance from regulatory agencies expert consortia. “Limited Clinical Relevance” denotes replicated associations with moderate-to-high evidence but without current guideline-based prescribing recommendations. “No Clinical Relevance” refers to weak, nonreplicated, or exploratory associations lacking sufficient evidence for clinical implementation.

cEVR, complete early virologic response; RVR, rapid virologic response.

∗ Only the bolded variant(s) in each row are associated with the stated clinical relevance.

IFNs are cytokines that play a key role in primary innate immune defense against infections. Their broad classification includes 3 families: type I (IFN-*α/β*), type II (IFN-*γ*), and type III (IFN-*λ*). Type III IFNs (IFNL4 or IFN-*λ*) are the most recently discovered family with overlapping yet different functions compared with other IFNs.^213^ The human *IFN-λ* locus is mapped to chromosome 19q13, and it consists of 4 genes: *IFNL1* (IL-29), *IFNL2* (IL-28A), *IFNL3* (IL-28B), and *IFNL4*. The rs12979860, which is located within intron 1 of *IFNL4*, 3kb upstream of the *IFNL3* gene, is the most robustly linked to spontaneous clearance of HCV and PEG-IFN/RBV treatment outcomes in genotype 1 patients with HCV. This was initially reported in 2009 in 3 independent GWASs.^214^, ^215^, ^216^ Since then, this association has been replicated extensively across studies.^217^ Another variant, rs8099917, which is in LD with rs12979860, was also among the top hits associated with response to PEG-IFN/RBV antiviral therapy. Carriers of rs12979860 (CC) or rs8099917 (TT) genotypes showed a higher probability of therapeutic success.^216^

In 2013, rs368234815 (ΔG/TT, later merged into rs11322783), a frameshift variant upstream of *IFNL3* in exon 1 of *IFNL4*, was shown to produce the novel *IFNL4* gene.^218^ Only individuals carrying the ΔG allele of rs11322783 can produce a functional IFNL4 protein. IFNL4 expression correlates with impaired spontaneous clearance and poor therapeutic response to HCV. *IFNL3* and *4* are genetically linked, and they share approximately 29% amino acid identity.^218^ Also, the *IFNL4*-TT/ΔG variant (rs11322783) is in very high LD with the *IFNL3* variant (rs12979860). The observed LD between these 2 variants is the highest among Asians (*r*^2^ = 1) and Europeans (*r*^2^ > 0.9), compared with African Americans, with an *r*^2^ of approximately 0.7.^219^

Given the role of the *IFNL4*-TT/ΔG allele in IFNL4 production and its association with IFN-based therapy, it has been proposed as the causal variant underlying the association between HCV clearance and the *IFNL3/4* locus, whereas rs12979860 could be a marker. Studies have also shown a significant association between the *IFNL3/4* locus variants and the expression of hepatic ISGs. High ISG levels were found in patients carrying IL-28B variants, which are also associated with a diminished response to IFN-based hepatitis C treatment.^220^ High ISG levels could indicate a preactivation of the hepatic IFN system without significant treatment-associated induction, which results in nonresponsiveness to IFN-based therapy.^221^ Production of IFNL4 protein has also been linked to the induction of negative regulators of the IFN response, such as USP18 and SOCS1, in primary human hepatocytes.^222^ This could at least explain the negative effect of IFNL4 on HCV treatment. Nonetheless, the mechanism by which the *IFNL3/4* locus influences antiviral response to HCV has not been fully described.

Several other variants in the *IFNL3/4* locus were also strongly associated with PEG-IFN/RBV treatment outcomes. This includes rs8103142, rs1188122, rs117648444, rs12980275, rs28416813, rs4803219, rs7248668, rs4803217, and rs8105790 (PharmGKB). The 2 main variants, rs12979860 and rs11322783, are both classified among level 1 variants in PharmGKB, given the strong evidence of their association with PEG-IFN/RBV response. The *IL-28B* variant, rs12979860, is also among the FDA pharmacogenomic biomarkers in drug labels. The FDA-approved drug label for PEGINTRON (peginterferon alfa-2b) states that the *IL-28B* variant (rs12979860) is associated with variable SVR rates of 66%, 30%, 22% among CC, CT, and TT genotype carriers, respectively.^223^ Moreover, the CPIC guidelines for PEG-IFN*α*-based regimens indicate the 2 possible phenotypes based on the rs12979860 genotype, where the CC allele carriers have an “increased likelihood of response” compared with patients carrying at least one unfavorable response allele (CT and TT). It is therefore recommended to “consider implications before initiating PEG-IFN/RBV containing regimens,” particularly when these regimens are not combined with PIs.^224^

Current guidelines recommend pan-genotypic DAAs for simplified, highly effective treatment across all HCV genotypes.^225^ This includes glecaprevir/pibrentasvir, sofosbuvir/velpatasvir, ledipasvir/sofosbuvir, or elbasvir/grazoprevir as first-line for treatment-naive patients without cirrhosis or with compensated cirrhosis. These regimens are prescribed for a duration that often ranges from 8 to 12 weeks for most patients. Sofosbuvir/velpatasvir/voxilaprevir regimen is also recommended as an alternative therapy for this patient cohort. For treatment-naive patients with decompensated cirrhosis, ledipasvir/sofosbuvir or sofosbuvir/velpatasvir are recommended with or without weight-based ribavirin for a duration of 12 or 24 weeks, respectively. Although DAA therapy is curative for most patients, a small proportion of patients do not respond and need a retreatment plan, which typically involves a resistance-guided approach using alternative DAA combinations, often incorporating a PI and extending the treatment duration.^225^

Although the pharmacogenomic determinants influencing response to PEG-IFN/RBV regimens have been well studied, there remains a noticeable gap in the PGx of the newer DAAs. Importantly, these combinations are metabolized primarily by P450 enzymes, including CYP3A and CYP2C8, and are substrates of transporters such as P-gp, breast cancer resistance protein, and OATP1B1. Nonetheless, the potential role of genetic variation in genes in relation to drug metabolism and transport remains an area worthy of further investigation. The currently available evidence is limited, with only a few associations reported between *IFNL3/4* genotypes and SVR after treatment with ledipasvir/sofosbuvir or sofosbuvir/velpatasvir/voxilaprevir. Moreover, most existing PGx data pertain to older IFN-based regimens, DAAs administered in combination with ribavirin and PEG-IFN, or to DAAs that are no longer recommended for use. For instance, it was found that the plasma concentrations of daclatasvir—a DAA no longer recommended—are influenced by polymorphisms in vitamin D pathway genes, specifically rs11568820 in vitamin D receptor (*VDR*) and rs2248359 in *CYP24A1*.^226^ Similarly, decreased trough concentration of telaprevir, another discontinued DAA, has been linked to the rs2585428 variant in *CYP24A1*.^227^ The lack of PGx studies on the currently used DAAs could be partially attributed to the consistently high SVR achieved across diverse populations and genotypes. Nonetheless, identifying genetic determinants influencing drug metabolism, toxicity, or treatment failure remains crucial, especially in populations with comorbidities or at risk of adverse events. One of the main concerns in combination therapy is the occurrence of unfavorable adverse events, among which is hemolytic anemia, which is severe enough to require reducing the initial dose in up to 15% of patients.^228^ Genetic variants causing inosine triphosphatase (ITPA) deficiency have been associated with this severe adverse event.^228^ Two candidate variants in the *ITPA* gene, rs1127354 (CC) and rs7270101 (AA), have been repeatedly linked to an increased risk of anemia in patients treated with PEG-IFN/RBV, as reported from different studies on multiple ethnic groups.^229^, ^230^, ^231^ A third SNP in the *ITPA*, rs6051702 (AC/CC genotype), which is in LD with rs1127354 and rs7270101, has also been reported as the only factor that predicted anemia in patients with HCV at week 4 of PEG-IFN/RBV treatment.^232^ A large meta-analysis that included 29 studies concluded that patients carrying the rs1127354 (CC), rs7270101 (AA), or rs6051702 (AA) genotypes are at 12.84, 3.41, and 4.43 are at a higher risk of developing severe anemia, respectively.^233^ The rs1127354 variant has also been associated with hemolytic anemia in patients treated with the new DAAs-RBV regimens, in addition to the association with PEG-IFN/RBV-induced anemia.^234^ Both rs1127354 and rs7270101 have a moderate level of evidence (2B) according to PharmGKB, based on the significant associations reported across multiple cohorts. However, there are no regulations or recommendations concerning these variants, and additional supporting evidence is still needed.

#### 3 Severe acute respiratory syndrome coronavirus 2 drugs

The importance of PGx was further underscored during the COVID-19 pandemic, when the rapid development and deployment of new therapies highlighted the need to understand the genetic factors that influence drug response. Genetic variation affects not only how patients respond to antivirals but also the supportive therapy, including corticosteroids and anticoagulants.^235^^,^^236^ During the SARS-CoV-2 pandemic, many repurposed antiviral drugs or supportive treatments without strong evidence of efficacy or magnitude of toxicity were used to mitigate the devastating consequences of the infection,^237^ often in the absence of biological plausibility.

The currently used antivirals for COVID-19 are nirmatrelvir-ritonavir, molnupiravir, and remdesivir. Nirmatrelvir is an oral antiviral drug that acts by inhibiting SARS-CoV-2 main protease (M^pro^).^238^ Inhibiting M^pro^ prevents the processing of the polyprotein precursors needed for virus replication.^239^ Nirmatrelvir is coadministered with a low dose of ritonavir, an HIV PI, which inhibits CYP3A4 enzyme activity that plays an important role in nirmatrelvir metabolism.^240^ This combination was authorized for emergency use in December 2021, and it is currently one of the highly effective antiviral drugs against COVID-19.^239^ It is intended for the treatment of patients with mild-to-moderate COVID-19 who are at high risk of progression to severe disease and is typically prescribed for a 5-day course, initiated within 5 days of symptom onset.^241^ Molnupiravir is an inhibitor of RNA-dependent RNA polymerase developed to treat SARS-CoV-2. It is also an oral antiviral used for nonhospitalized adults with mild to moderate COVID-19 who are at high risk of progressing to severe disease. Molnupiravir is administered over 5 days and is authorized only when alternative FDA-approved COVID-19 treatments are not accessible or clinically appropriate.^242^ Remdesivir, on the other hand, is administered intravenously and is mainly used for hospitalized or nonhospitalized patients with mild to severe COVID-19. It is a monophosphoramidate nucleoside analog that was developed to treat Ebola and other viruses with pandemic potential and was subsequently FDA-approved for the treatment of COVID-19. The standard treatment time varies from 3 to 10 days, depending upon the severity of the disease and the patient’s clinical response.^243^

ADRs have also been observed with the use of antivirals and supporting treatment in COVID-19. A study evaluated ADRs in 155 patients with COVID-19 and reported a 72.3% incidence, most of which were hepatic, gastrointestinal, hematological, and endocrine events. Although most ADRs were associated with hydroxychloroquine (originally an antimalarial and anti-inflammatory drug), which has proven ineffective and has serious concerns about adverse events, other drugs also contributed to ADRs, including lopinavir-ritonavir (ARV for HIV), favipiravir (influenza antiviral), and ribavirin (originally developed for hepatitis C and viral hemorrhagic fevers).^244^ A larger study that analyzed the data from 7365 hospitalized patients, 2682 (36.4%) of whom had COVID-19, found that the incidence rate of serious ADRs in patients with COVID-19 was 760.63 per 10,000. These events were associated with the use of repurposed drugs for COVID-19, with tocilizumab (rheumatoid arthritis/cytokine release syndrome drug) being the most frequently related, followed by dexketoprofen (nonsteroidal anti-inflammatory drug), azithromycin (macrolide antibiotic), lopinavir-ritonavir (ARV for HIV), dexamethasone (corticosteroid), and chloroquine/hydroxychloroquine (antimalarial and anti-inflammatory).^245^ A retrospective observational study in patients with severe COVID-19 reported that baricitinib—another rheumatoid arthritis drug—was associated with significantly fewer adverse events compared with tocilizumab.^246^ Baricitinib is recommended in combination with corticosteroids for use in severe/critical COVID-19 when alternatives to IL-6 blockers are not available (eg, tocilizumab). These reports highlight the importance of personalized regimens, particularly in high-risk groups, including the elderly and those with preexisting hepatic, renal, or cardiovascular conditions, who are often more vulnerable to side effects. The high rate of ADRs seen in COVID-19 treatments makes it clear that careful pharmacovigilance, integrating pharmacogenetic insights to mitigate harm and improve outcomes, is needed.

Host genetics has gained significant attention during the SARS-CoV-2 pandemic. Many groups around the world explored the genetic determinants of infection susceptibility and severity, including the largest GWAS in history, which included up to 125,584 cases and over 2.5 million control individuals across 60 studies from 25 countries.^247^ These studies revealed compelling insights about SARS-CoV-2 susceptibility and severity. The most robust association is with the 3p21.31 gene cluster.^248^ This locus encompasses genes such as *LZTFL1*, *SLC6A20*, *CCR9*, and *CXCR6*, all of which play roles in immune responses and pulmonary function. Multiple other genetic loci, including those at the *ACE2*, *MUC5B*, *SFTPD*, *OAS1*, *FOXP4*, *ABO*, and *IFNAR2* genes, have been strongly associated with infection outcomes.^247^^,^^249^ Although these associations could reveal biological mechanisms of therapeutic relevance and represent potential biomarkers for drug response, further efforts are needed to accelerate downstream research in this field.

Several currently used anti-SARS-CoV-2 agents are metabolized by P450 enzymes and transported by membrane-bound proteins, both of which are subject to genetic variability that may influence drug response. For instance, nirmatrelvir is a substrate of the efflux pump P-gp (*ABCB1* gene) and is also metabolized by CYP3A4. Genetic variation in drug metabolism and transport pathways could theoretically influence nirmatrelvir-ritonavir exposure. This includes variants in *ABCB1* (rs1045642), *CYP3A5* (rs2740574), *SLCO1B1* (rs4149056), and *APOC3* (rs2854117) that have been previously associated with ritonavir PK, efficacy, and/or toxicity in patients with HIV.^237^^,^^250^ However, no validated pharmacogenetic associations have been established in COVID-19 cohorts, and no genotype-guided dosing recommendations currently exist.

Remdesivir also undergoes extensive metabolism by multiple P450 enzymes (CYP3A4, CYP2C8, and CYP2D6) as well as carboxylesterase 1 (CES1) and cathepsin A.^238^ In addition to enzymatic metabolism, remdesivir is a substrate for the uptake transporter OATP1B1 and the efflux transporter P-gp.^251^ In a retrospective study analyzing data from 4125 hospitalized patients, intermediate or poor CYP2C19 metabolizers taking remdesivir experienced higher alanine aminotransferase levels than normal, rapid, or ultrarapid metabolizers.^252^ This reported association has not been independently replicated in external cohorts, and clinical actionability remains unestablished. Patients with COVID-19 receiving remdesivir may have variable outcomes if they carry genetic mutations in *CES1* and/or other metabolism/transport-related genes, but this requires further research.

CES1 and CES2 are key enzymes in the hydrolytic activation of molnupiravir, converting it into the ribonucleoside analog *β*-D-N4-hydroxycytidine. *β*-D-N4-Hydroxycytidine is then taken up by host cells through nucleoside transporters, including CNT1, CNT2, CNT3 (encoded by *SLC28A1–3*), and ENT2 (*SLC29A2*).^238^ Human genetic variability in these genes may interfere with the conversion of molnupiravir to *β*-D-N4-hydroxycytidine, leading to interpatient variability in the therapeutic outcomes. Although such pharmacogenetic interactions are plausible, they remain theoretical, lack clinical validation, and currently have no implications for genetic testing or treatment selection.

The response to anti-inflammatory agents, such as corticosteroids, is also influenced by genetic polymorphisms. For instance, variants in *NR3C1* (rs6198, rs33388, and rs33389) and *CYP3A4* rs35599367 are associated with response to dexamethasone, a glucocorticosteroid used to treat COVID-19 and other conditions.^253^ Patients with SARS-CoV-2 carrying the rs6198-CC, rs33389-TT, or rs33388-AA of *NR3C1* exhibited an increased time to respond to dexamethasone. Importantly, patients with SARS-CoV-2 and hyperinflammation that results from the dysregulated release of inflammatory mediators often require anti-inflammatory treatment beyond glucocorticoids, such as IL-6 inhibitors, IL-1 inhibitors, and Janus kinase (JAK) inhibitors. Although this has still not been explored in COVID-19 cohorts, these anti-inflammatory biologics are influenced by genetic variation, and their efficacy and safety can be optimized through PGx-guided approaches. For instance, previous studies have shown that tocilizumab (an anti-IL-6 receptor monoclonal antibody) and anakinra (an IL-1 receptor antagonist) may be associated with drug reaction with eosinophilia and systemic symptoms in patients with arthritis, juvenile rheumatoid or Still disease carrying the *HLA-DRB1*∗15:01 allele.^254^ Tocilizumab efficacy has also been linked to variations in *CD69* (rs11052877), *FCGR3A* (rs396991), *GALNT18* (rs4910008), and *IL-6R* (rs12083537, rs11265618, and rs35717427) in multiple studies.^255^, ^256^, ^257^, ^258^ Although these associations have been primarily studied in autoimmune conditions, their relevance to COVID-19 remains unproven, and extrapolation across disease contexts should be approached with caution.

A promising strategy to suppress hyperinflammation involves targeting the JAK/signal transducer and activator of transcription signaling pathway. Baricitinib, a JAK1/2 inhibitor, was the first approved immunomodulatory stand-alone treatment for COVID-19.^259^ Currently, PGx studies on baricitinib are lacking. However, its PK profile involves a few potentially important pharmacogenes, including *CYP3A4* and *SLC22A8* encoding the OAT3 transporter.^260^ Ruxolitinib is another JAK1/2 inhibitor that is currently being investigated. Studies have shown that ruxolitinib proved to be safe and effective in patients with defined hyperinflammation.^261^ Ruxolitinib is mainly metabolized by CYP3A4 and CYP2C9, both of which are among the “Very Important Pharmacogenes (VIP)” in PharmGKB database.^262^^,^^263^ This suggests that genetic variations in baricitinib or ruxolitinib pathway-related genes may contribute to their efficacy and safety in patients with COVID-19.

Genetic variants may influence not only drug metabolism and transport but also treatment efficacy and the risk of long COVID, also known as postacute sequelae of SARS-CoV-2 infection. Long COVID consists of a heterogeneous constellation of more than 200 symptoms and a pathophysiology that is yet to be understood.^264^ With this complexity and variability, PGx studies may help understand differential drug responses in the future. A recent report investigated the presence of genetic variants in the *ABCB1*, *PXR*, *CYP2D6*, and *CYP3A4* genes in patients with prolonged COVID-19 treated with nirmatrelvir/ritonavir and remdesivir.^265^ The study highlighted the importance of genetics in improving the outcomes of long COVID-19. However, the sample size was too small to find significant correlations. One of the most concerning risks linked to long COVID-19 is venous thromboembolism, particularly pulmonary embolism, which affects up to 23.8% of patients with long COVID-19.^266^ Given that thromboinflammation is a major cause of morbidity and mortality in COVID-19, anticoagulation has become an integral part of the treatment. Nevertheless, direct oral anticoagulants use is limited by the drug–drug interactions with a variety of anti-COVID therapeutic drugs.^267^ Therefore, parenteral anticoagulants, low molecular weight heparin, or unfractionated heparin are recommended for hospitalized COVID-19 patients receiving antiviral therapy. Vitamin K antagonist (eg, warfarin) can also be used for patients who were already on warfarin before COVID-19.

Genetic variability in genes related to anticoagulants has been previously documented. Among therapies used in COVID-19–related care, warfarin anticoagulation represents the most well established example of clinically actionable pharmacogenetics, supported by regulatory labeling and guideline recommendations. The H131R polymorphism of the *FCGR2A* (CD32A) gene has been consistently associated with heparin-induced thrombocytopenia-associated thrombosis.^268^ The dose variability in warfarin also has strong interindividual genetic determinants. It was found that common genetic polymorphisms in *CYP2C9*, *VKORC1*, *CYP4F2*, and the *CYP2C* cluster, besides known nongenetic factors, account for 50% of warfarin dose variability. Therefore, genetic-guided dose optimization is recommended by the CPIC and FDA to improve the safety of warfarin.^269^ This highlights the critical role of PGx in guiding anticoagulation strategies and demonstrates its potential implications, particularly for patients with COVID-19 at increased risk of thromboembolic complications. Building on this, expanding the evaluation of further PGx biomarkers in SARS-CoV-2 antiviral and supporting drugs (new and repurposed), utilizing the availability of genomics data and using modern technologies, would accelerate the development and clinical implementation of personalized therapies not only in acute COVID-19, but also in the prevention and management of long COVID.

Importantly, despite the widespread use of antiviral, anti-inflammatory, and anticoagulant therapies in COVID-19, no pharmacogenetic biomarkers, except the established regulations for warfarin, are currently considered clinically actionable or recommended for routine genetic testing in patients with COVID-19. Most PGx associations discussed above are exploratory or extrapolated from other diseases and should be interpreted as hypothesis-generating rather than as guidance for clinical decision-making.

#### 4 Other antiviral drugs

The lack of sufficient PGx data on COVID-19 therapies may be attributed to the relatively recent emergence of the virus in the human population. However, there is also a large gap in the PGx of other viruses circulating for a long period. As genetic variability of the host is considerably low compared with viruses, most PGx studies focused on characterizing mutations in the viral genomes that may lead to drug resistance or complicate the use of antivirals and monoclonal antibodies. A limited number of host gene–drug associations have been identified so far (Table 4).^252^^,^^253^^,^^270^, ^271^, ^272^, ^273^ This includes variations in the *ABCB1* (rs1045642) and *CES1* (rs200707504) that have been linked to oseltamivir metabolism and toxicity.^270^^,^^271^ Oseltamivir is considered the cornerstone for influenza treatment, and it is 1 of 4 FDA-approved influenza antiviral drugs recommended by the Centers for Disease Control and Prevention in the 2022 season, along with baloxavir/marboxil, zanamivir, and peramivir.^274^ Another polymorphism in *ABCB1* (rs2229109) has been linked to neutropenia in renal transport patients receiving valganciclovir, a key antiviral for cytomegalovirus prevention and treatment.^272^ This drug is associated with challenging adverse hematological events in immunosuppressed patients, leading to resistance in some cases. The variant rs11568658 in the *ABCC4* gene was also significantly associated with neutropenia in the same study. Letermovir is an antiviral drug used to prevent cytomegalovirus reactivation in immunocompromised patients. Significant associations were found between variants in *SLCO1B1* (rs4149032 and rs4149056) and *UGT1A* (rs4148323) and letermovir PK.^273^ The study suggests that variants of enzymes and transporters may explain some variability in letermovir PK but do not affect exposure to a clinically relevant level.^273^ Of note, none of these PGx findings were replicated in other studies or subjected to clinical guideline recommendations.

**Table 4:** Pharmacogenomic variants associated with different phenotypes in response to antiviral drugs

Antiviral Drug	Class	Virus	Effect	Gene	Key Variants	Sample Size	Clinical Relevance	Reference
Remdesivir	Antiviral	SARS-CoV-2	Liver enzyme elevation (ALT)	*CYP2C19*	Intermediate/poor metabolizers	4125	No clinical relevance	252
Dexamethasone	Corticosteroid	SARS-CoV-2	Efficacy – longer hospitalization	*NR3C1*	rs6198-CC rs33389-TT rs33388-AA	103	No clinical relevance	253
				*CYP3A4*	rs35599367-TC	103	No clinical relevance	253
Oseltamivir	Antiviral	Influenza	Depression, gastritis, or hypersensitivity	*ABCB1*	rs1045642-AG and GG	310	No clinical relevance	270
			Increased concentration	*CES1*	rs200707504-CT	20	No clinical relevance	271
Valganciclovir	Antiviral	CMV	Neutropenia	*ABCC4*	rs11568658-AC	174	No clinical relevance	272
			Neutropenia	*ABCB1*	rs2229109-CT	174	No clinical relevance	272
Letermovir	Antiviral	CMV	Decreased exposure	*UGT1A1*	rs4148323	296	No clinical relevance	273
			Decreased exposure (AUC)	*SLCO1B1*	rs4149032-TT	296	No clinical relevance	273
			Increased exposure (AUC)	*SLCO1B1*	rs4149056-CC + CT	296	No clinical relevance	273

ALT, alanine aminotransferase; AUC, area under the concentration–time curve; CMV, cytomegalovirus.

Despite its promising role in understanding interindividual variability in drug response and improving patient care, PGx influencing the efficacy and toxicity of antiviral drugs (excluding HIV and HCV therapies) remains largely understudied.

### B Pharmacogenomics of antibacterial drugs

The discovery of antibiotics remains one of the greatest discoveries in medicine. Although natural antibiotics have been used for millennia to treat bacterial infections, it was not until the late 19th century that scientists began to detect antibacterial substances in action. With the discovery of arsphenamine by Paul Ehrlich in 1909, penicillin by Alexander Fleming in 1928, and Prontosil (sulfonamides) by Gerhard Domagk in 1935, and the golden era of antibiotic discovery (1940s–1960s), the history of ID has been completely changed.^275^ In that era, a large number of antibiotics were discovered and used for preventing and treating bacterial infections, many of which are still in clinical use today. These discoveries have saved millions of lives, significantly reducing mortality from bacterial infections.

Despite saving lives, the effectiveness of antibiotics is overshadowed by the alarming upsurge in antibiotic resistance. This resistance has emerged as a major global health threat, undermining the efficacy of antibiotics in treating infections and reversing the advancements achieved over the past century. Additionally, antibiotic use has been associated with the development of short- and long-term ADRs that are another serious public health problem with respect to mortality, morbidity, and healthcare costs.

Antibiotics have contributed to hospital admissions because of ADRs (as comprehensively reviewed by Silva et al^22^). It is estimated that over 50% and 70% of inpatients and intensive care unit patients, respectively, receive antibiotics.^276^ Although antibiotics appear to result in a relatively low proportion of ADRs, their widespread use contributes to approximately 23% of all documented ADRs.^22^ Among patients receiving antibiotics, the incidence of ADRs ranges from 19% in Uganda, 20% in the United States, 25% in Australia, and 28.3% in Morocco, as reported in previous studies.^277^, ^278^, ^279^, ^280^ Among all ADRs reported across different drug types, antimicrobial drugs contributed to 26.9% of 2349 ADRs reported in Saudi Arabia.^281^ Anti-infective agents were also responsible for 40.92% of hospital admissions because of ADRs in India.^282^

The regional variability in the rate of antimicrobial-associated ADRs could be attributed to differences in the antibiotics prescribing practices, healthcare infrastructure including medical reporting systems, regional differences in access to healthcare, and antibiotic stewardship programs. In addition, host-related factors such as genetic diversity may influence the susceptibility to ADRs and contribute to interpopulation and interindividual differences in antibiotic-associated ADRs. It is important to highlight that these ADR statistics are not merely numbers, as they reflect a significant clinical and public health burden. High rates of antibiotic-associated ADRs can lead to treatment discontinuation, suboptimal therapy, increased risk of resistance development, and higher morbidity and mortality, particularly in regions with limited healthcare resources or pharmacovigilance systems. Addressing these challenges requires a proactive understanding of ADR risk factors and implementation of effective prevention strategies.

In recent years, PGx has gained increasing recognition as an important component in understanding why individuals respond differently to antibiotics. This variability often reflects the complexity of host-pathogen-drug interactions. From the host side, variability in PK and PD profiles, which are influenced by the patients’ PGx, can influence the predisposition to antibiotic-related ADRs, toxicity, and drug effectiveness (Table 5).^47^^,^^48^^,^^56^^,^^283^, ^284^, ^285^, ^286^, ^287^, ^288^, ^289^, ^290^, ^291^, ^292^, ^293^, ^294^, ^295^, ^296^, ^297^, ^298^, ^299^, ^300^, ^301^ Interindividual variation in genes encoding phase I and phase II hepatic enzymes, membrane transporters, and HLA class I and class II were linked to antibiotic-associated ADRs and are of a particular interest.^302^ Importantly, the growing recognition of PGx in antibiotic therapy offers valuable insights and could pave the way for optimizing its safe use.

**Table 5:** Pharmacogenomic variants associated with broad-spectrum and anti-TB antibiotics

	Associated Drugs	Class	Effect	Gene/Region	Key Variants	OR	Clinical Relevance	Reference
Broad spectrum	AGs	AGs	AIHL	*MT-RNR1*	m.1494C>T (rs267606619), m.1095T>C (rs267606618), m.1555A>G (rs267606617)	NA	Guideline-supported	283
	AC	B-lactam	DILI	*HLA*	*HLA-DRB1*∗15:01, *HLA-A*∗30:02; *HLA-B*∗18:01; *HLA-DRB1*∗15:01-*HLA-DQB1*∗06:02; A∗02:01; B∗15:18	1.7–6.7	No clinical relevance	284, 285, 286
	Flucloxacillin	B-lactam	DILI	*PTPN22*	rs2476601 (G)	1.4	No clinical relevance	287
				*ERAP2*	rs1363907 (G)	1.68	No clinical relevance	288
				*HLA*	*HLA-B*∗57:01	Up to 80	Limited clinical relevance	289
				*PXR*	rs3814055 (C-25385T)	3.37	No clinical relevance	290
	Cefotaxime	B-lactam	↓ Concentration	*ABCC2*	rs2273697 (GG)	NA	No clinical relevance	291
			↓ CSF/plasma ratio	*ABCG2*	rs13120400 (C)	NA	No clinical relevance	291
	Sulfamethoxazole (SMX) + trimethoprim (TMP)	Sulfonamides	SCAR in patients with AIDS	*NAT2*	∗16, ∗5, ∗6, ∗7, ∗14 (slow acetylators)	2.5	No clinical relevance	47
			SCAR	*HLA*	***HLA-B*∗13:01, *HLA-B*∗15:02, *HLA-B*∗38:02, *HLA-C*∗06:02, *HLA-C*∗07:27, and *HLA-C*∗08:01,**	Up to 40	Limited clinical relevance∗	292, 293, 294
			SMX-TMP-induced liver injury (SILI)	*NAT2*	rs1495741 (AA), rs1041983 (TT)	2.49	No clinical relevance	295
			Respiratory failure	*HLA*	*HLA-B*∗07:02/*HLA-C*∗07:02	NA	No clinical relevance	296
			DILI (liver injury)	*HLA*	*HLA-B*∗14:01, *HLA-B*∗35:01	2.8–5.5	No clinical relevance	297
	Azithromycin	Macrolide	↑ Plasma concentration	*ABCB1*	rs2032582 (2677GG), rs1045642 (3435CC)	NA	No clinical relevance	298
	Erythromycin	Macrolide	↑ Metabolism, ↓ transport	*ABCC2, SLCO1B1*	rs717620 (TT); OATP1B1∗5 (V174A), rs4149056 (c.521T>C)	NA	No clinical relevance	56,299
Anti-TB	Isoniazid (INH)	Anti-TB (first-line)	Hepatotoxicity	*NAT2*	*NAT2*∗5, ∗6, ∗7, ∗14, or ∗16	Up to 9 (∗6 and ∗6/∗7)	Limited clinical relevance	300,301
			ATDH	*CYP2E1, GSTM1*	*CYP2E1*∗1A/∗1A; *GSTM1* null/null	Up to 1.7	No clinical relevance	48

Clinical relevance was defined as: “Guideline-Supported,” indicates the highest level of clinical relevance supported by prescribing guidance from regulatory agencies expert consortia. “Limited Clinical Relevance” denotes replicated associations with moderate-to-high evidence but without current guideline-based prescribing recommendations. “No Clinical Relevance” refers to weak, nonreplicated, or exploratory associations lacking sufficient evidence for clinical implementation.

CSF, cerebrospinal fluid; NA, not available.

∗ Only the bolded variant(s) in each row are associated with the stated clinical relevance.

#### 1 Broad-spectrum antibiotics

##### a Aminoglycosides

Aminoglycosides (AGs) are a potent broad-spectrum class of antibiotics and were among the first to be used in treating clinically important gram-positive bacteria.^303^ AGs remain a cornerstone of antibacterial chemotherapy and include those frequently prescribed: neomycin, streptomycin, gentamycin, netilmicin, tobramycin, and amikacin.^304^ AGs inhibit bacterial protein synthesis through high-affinity binding to the aminoacyl site of 16S ribosomal RNA (a component of the ribosomal 30S subunit), leading to error-prone protein synthesis.^305^ Eventually, this leads to incorrect assembly of amino acids, accumulation of truncated and malfunctioning proteins, and bacterial cell damage.^303^ Because of their retained activity against multidrug-resistant pathogens, synergistic activity with different antibacterial classes, and low cost, AGs have regained clinical interest and are remain frequently prescribed in clinical practice.^304^ AGs (alone or combined with other antibiotics) are used to prevent and treat life-threatening sepsis in newborns or immunocompromised patients, treat recurrent and resistant TB, and eliminate *Pseudomonas aeruginosa* in patients with cystic fibrosis.^306^ Despite their effectiveness, AGs have been linked to toxic adverse events, among which nephrotoxicity and ototoxicity are the most significant. Nephrotoxicity and ototoxicity affect 2%–45% and 10%–25% of patients, respectively.^307^ Although nephrotoxicity due to AGs is generally reversible, the ototoxicity that leads to AG-induced hearing loss (AIHL) is irreversible.^308^^,^^309^ Permanent ear damage caused by ototoxicity affects either the cochlea, the vestibule, or both. Gentamicin, streptomycin, and tobramycin are known to cause vestibular ototoxicity, whereas amikacin, neomycin, and kanamycin are mainly cochleotoxic.^310^ Although hearing loss is a serious side effect of AGs at all ages, the complications are more deleterious for young children as they extend beyond deafness to impairment of language acquisition, psychosocial development, and education.^306^ It is estimated that up to 57% of children receiving AGs are at risk of developing AGs-related hearing loss, with those treated with gentamicin being at the highest risk.^311^ Several management strategies are suggested to mitigate AGs-related nephrotoxicity and ototoxicity risks, particularly in vulnerable populations. For nephrotoxicity, it was found that patients without a previous history of chronic kidney disease were at a higher risk of acute kidney injury, especially those with relatively low albumin (< 3.0 g/dL) or low hemoglobin (< 11.6 g/dL).^312^ Notably, individual biological differences between patients can influence the severity of the outcomes. This highlights the importance of risk prediction, patient selection, and dose adjustment before AGs prescription. Additionally, close monitoring and detection of markers for kidney injury, including serum creatinine (traditional marker) and urinary NGAL (new marker) will be useful, especially in critically ill elderly patients.^313^ Currently, supportive care and drug discontinuation are the only available strategies to prevent further damage.^309^ On the other hand, the management of ototoxicity includes dosing modifications (lowest dose for shortest period), utilizing preventive agents (eg, liproxstatin-1, heat shock protein inducers, sodium selenite, and antioxidants), and monitoring changes in hearing using audiometry and biomarkers such as caspase-3 (Reviewed in Le et al^309^).

AIHL occurs when AGs enter the inner ear tissue and cause sensory hair cell degeneration. These drugs cross the blood-labyrinth barrier in the inner ear and enter the endolymph. Once in the endolymph, AGs enter the hair cells through mechanoelectrical transduction and apical endocytosis.^314^ The accumulation of AGs in lysosomes of hair cells leads to lysosome enlargement, risk of rupture, and hair cell death. In addition, AGs cause ototoxicity via overproduction of free radicals in the mitochondria, which also results in cell death.^315^ Interestingly, mammalian mitochondrial ribosomes are similar to prokaryotic 70S ribosomes. Hence, AGs can bind to the 12S ribosomal RNA subunit of the mitochondria and impact ribosomes in a manner similar to that in bacteria.^316^ Several risk factors contribute to ototoxicity, including genetic predisposition, age, renal insufficiency, depletion of endogenous antioxidants, and ischemia/hypoxia, among multiple others.^317^^,^^318^ Ototoxicity has been linked to the use of high AGs doses. However, genetically predisposed individuals experience severe AIHL even with the administration of the standard recommended AG dose.^304^

**Fig. 4:**
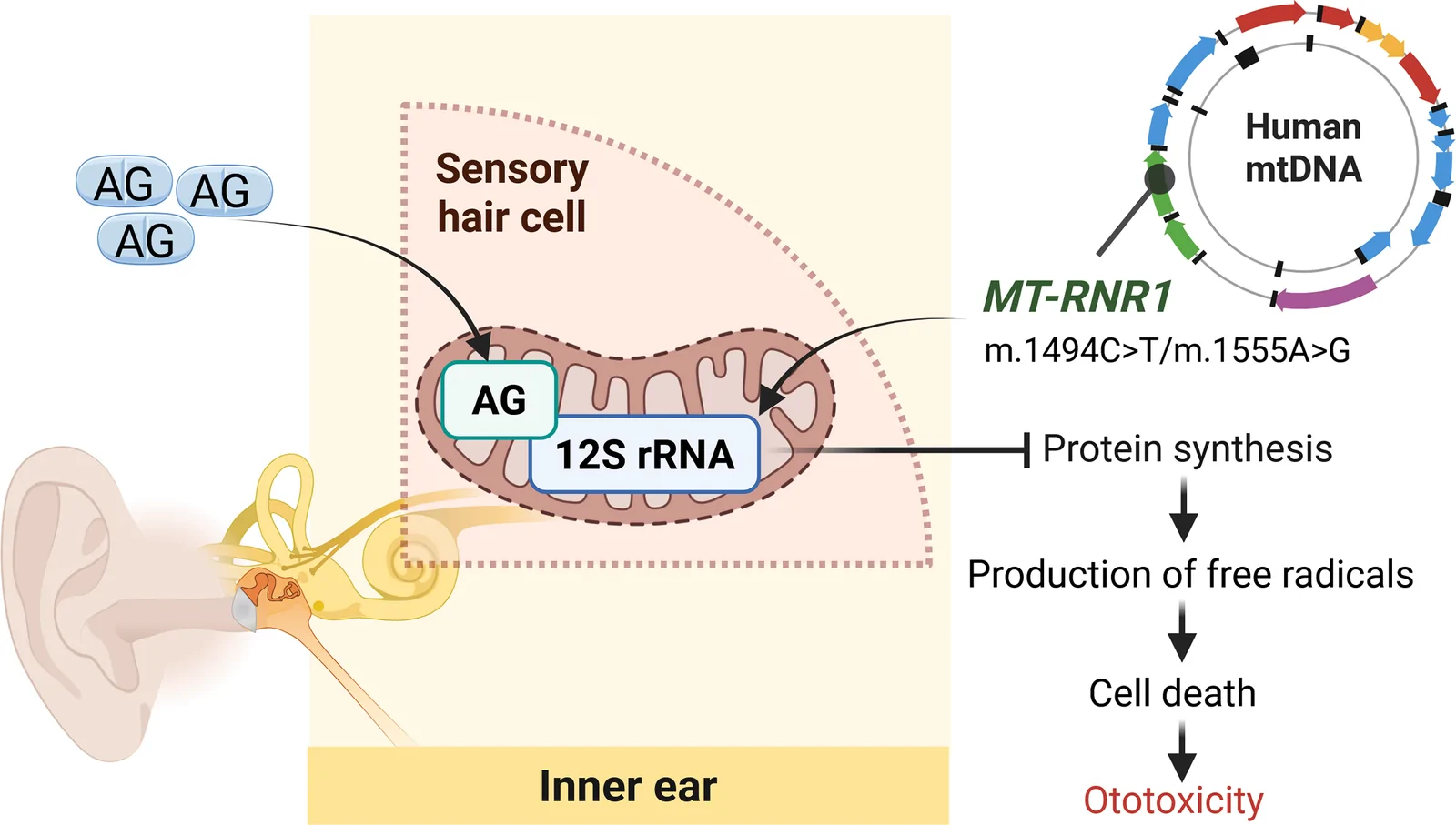
The mechanism of AG-induced ototoxicity in genetically susceptible individuals. Mutations within mt-RNR1 increase the similarity of the 12S ribosomal subunit to the 16S bacterial subunit by affecting its structure. This enhances 12S ribosomal RNA (rRNA) binding to AGs, inhibits protein synthesis, induces the production of free radicals, and eventually leads to cell death and ototoxicity. The most robust examples are the *MT-RNR1* m.1494C>T and m.1555A>G. mtDNA, mitochondrial DNA. Created in BioRender. Smatti, M. (2026) https://BioRender.com/dbn630i.

Human mitochondria have a genome that consists of 37 genes, of which 24 encode mature RNA products (22 transfer RNA molecules, 1 16S ribosomal RNA (rRNA) unit, and 1 12S rRNA unit).^319^ Studies have found that mutated mitochondrial DNA (particularly 12S rRNA) enhances AGs binding. This, in turn, inhibits protein synthesis, induces the production of free radicals, and eventually leads to ear damage.^316^ The 12S mitochondrial ribosomal subunit is encoded by the mitochondrial ribosomal RNA 1 gene (*MT-RNR1*). Mutations within this gene increase the similarity of the 12S ribosomal subunit to the 16S bacterial subunit by affecting its structure. Among all the identified *MT-RNR1* mutations, m.1494C>T (rs267606619) and m.1555A>G (rs267606617) demonstrated the strongest level of association with AIHL (Fig. 4). Both mutations appear to cause conformational change to the mitochondrial 12S rRNA, thus allowing AGs to bind more readily.^320^^,^^321^ Another variant, m.1095T>C (rs267606618), has also been linked to AIHL, although with a moderate level of evidence according to the CPIC.^283^ Multiple other variants within *MT-RNR1* have also been associated with AIHL; however, most have been reported in only 1 or 2 studies (eg, m.669T>C, m.747A>G, m.786G>A, m.807A>C, m.807A>G, m.839A>G, m.896A>G, m.930A>G, m.951G>A, m.960C>del, m.988G>A, m.1189T>C, m.1243T>C, m.1520T>C, m.1537C>T, and m.1556C>T). Few other associations had conflicting reports, as they were reported in patients with AIHL as well as patients without AIHL (eg, m.663A>G and m.961T>del+Cn). A complete list of all identified *MT-RNR*1 alleles and their functions is accessible with the CPIC guideline for *MT-RNR1* and AGs.^283^

In summary, in patients carrying an *MT-RNR1* increased risk variant for AIHL (m.1095T>C, m.1494C>T, or m.1555A>G), the CPIC strongly recommended to “avoid aminoglycoside antibiotics unless the high risk of permanent hearing loss is outweighed by the severity of the infection and lack of safe or effective alternative therapies.” For patients with a normal risk allele (m.827A>G), as well as uncertain risk variants, AGs can be used, although the risk of AIHL is not eliminated, especially with prolonged use of AGs or high doses.^283^

Routine genetic testing is not currently mandated. The FDA drug labels for AGs drugs include warnings and/or precautions regarding *MT-RNR1* pharmacogenetic variants. According to the PharmGKB, these labels are under “actionable PGx” because they contain information on variants/genotypes/phenotypes but do not require or recommend genetic testing. However, a recent report showed that point-of-care (POC) genotyping of the *MT-RNR1* m.1555A>G variant can be feasibly implemented for neonates in clinical settings. The test is rapid (in 26 minutes) and can be integrated into routine clinical practice without disruption.^322^ Three patients carrying the m.1555A>G variant were identified, none of whom were subjected to AG therapy. The utilization of such POC genotyping approaches before antibiotic prescribing could optimize the therapy outcomes in both children and adults. In an ongoing clinical trial, POC testing of *NAT2* genotypes is being evaluated for guiding isoniazid dosing in tuberculosis (NCT05413551). This growing body of translational PGx research—including POC testing—is critical for improving the safety and effectiveness of antibiotic therapy. Importantly, the effectiveness of genotyping implementation is context-dependent and varies according to multiple factors, including healthcare infrastructure, clinical utility, and cost-effectiveness. Hence, healthcare institutes are encouraged to assess the local feasibility of incorporating such testing into antibiotic stewardship programs and determine whether the benefits observed in previous trials can be replicated or tailored to local clinics.

##### b β-Lactams

Since their discovery in the 1920s, *β*-lactam antibiotics have become among the most commonly used antibiotics. *β*-Lactams account for 65% of all prescriptions for injectable antibiotics in the United States.^323^ This drug class is characterized by the presence of a 3-carbon and 1-nitrogen ring (*β*-lactam ring), which is highly reactive. *β*-Lactams work by interrupting bacterial cell wall formation through binding to penicillin-binding proteins, which are involved in the biosynthesis of peptidoglycan, the main component of the bacterial cell wall.^324^ There are 4 *β*-lactam subclasses: penicillins, cephalosporins (including the cephamycins), monobactams, and carbapenems. Because of their high effectiveness, broad spectrum activity, and generally low toxicity, they have been used to treat a wide range of mild to severe bacterial infections. *β*-Lactams are considered safe and well tolerated compared with other antibiotics; however, HSRs may occur. This includes immediate HSR, clinically manifested as urticaria, angioedema, bronchospasm, hypotension, or anaphylaxis in severe cases. On the other hand, *β*-lactam-induced delayed HSR can occur as SJS/TEN.^325^ HSRs affect 14%–20% of patients, with penicillin alone accounting for 33% and 39% of immediate and delayed HSRs, respectively.^326^

Among the *β-*lactams, amoxicillin (from the aminopenicillins subclass) is widely used to treat gram-positive and some gram-negative bacteria, specifically *β*-lactamase–negative strains.^327^ This antibiotic is often combined with the *β*-lactamase inhibitor clavulanic acid, a suicide inhibitor that binds *β*-lactamases produced by gram-positive and gram-negative bacteria, preventing the inactivation of the penicillin.^284^ This combination extends the antibacterial activity of amoxicillin and broadens its spectrum to include *β*-lactamase-producing strains. Amoxicillin clavulanate (AC) is generally safe as it causes temporary mild to moderate side effects, but a severe form, idiosyncratic DILI, has also been described.^328^ A recent study analyzed DILI cases according to the Roussel Uclaf Causality Assessment Method (RUCAM) reported that AC was the top drug implicated in causing DILI with a verified RUCAM diagnosis (*n* = 333 DILI cases). Flucloxacillin (*β*-lactam) and erythromycin (macrolide) were also among the top 10 drugs with RUCAM-verified DILI cases.^329^ A genetic basis has been suggested for AC-induced hepatotoxicity, in which drug hapten presentation via HLA class II is proposed as an underlying mechanism.^284^ Genetic studies demonstrated that HLA class II allele *DRB1*∗15:01 carriers are 2.6–10 times more susceptible to AC-induced DILI.^284^ The role of HLA class II in DILI has been evident in another study that found a strong association with rs9274407, which correlated with rs3135388, a tag SNP of *HLA-DRB1*∗15:01-*DQB1*∗06:02*.*^286^ Further, *HLA-A*∗02:01 was independently associated with DILI. Despite the strong association indicating an immunological mechanism, HLA genotypes have low positive predictive value and therefore limited utility as predictive or diagnostic biomarkers.^286^ A subsequent study found that class I *HLA* alleles A∗30:02 and B∗18:01 were associated with an increased risk of DILI with AC (OR = 6.7 and 2.9, respectively).^285^ The haplotype *HLA-DRB1*∗15:01-*DQB1*∗06:02 was also more frequent in DILI cases with cholestatic/mixed damage (OR = 5.2). Notably, most of these associations were reported from relatively small cohorts of European descent (*n* = 20–201 cases). More recently, a larger multiethnic study (*n* = 2048 DILI cases) discovered and replicated the association of liver injury induced by multiple drugs with the presence of the rs2476601 SNP, which is located in the protein tyrosine phosphatase, nonreceptor type 22 gene (*PTPN22*) (OR = 1.4, *P* = 1.2 × 10^−9^). Importantly, the strongest association with DILI was in patients of European ancestry (OR = 1.62, *P* = 4.0 × 10^−6^). Moreover, this variant doubles the risk of DILI among *HLA-A*∗02:01 and *DRB1*∗15:01 carriers, further supporting the possible role of immune dysregulation in idiosyncratic DILI.^287^ It was found that amoxicillin-modified peptides bind selectively to *HLA-DRB1*∗15:01 and/or *DQB1*∗06:02 risk haplotypes and stimulate T cells in patients with DILI. This provided a proof-of-concept that the binding of drug haptenated peptides to HLA class II risk alleles may explain the idiosyncratic DILI.^330^

Utilizing transcriptome-wide association and GWAS approaches, novel genetic risk factors of AC-DILI were recently discovered. Lower liver expression of the *ERAP2* gene correlated significantly with AC-DILI risk (*P* = 3.7 × 10^−7^).^288^ The lead expression quantitative trait locus SNP in the *ERAP2* region, rs1363907 (G), was identified as the single variant with the strongest association with AC-DILI in discovery and validation analyses (OR = 1.68 and 1.2, respectively). A novel association between *HLA-B*∗15:18 and AC-DILI was also found in both discovery and validation cohorts (OR = 4.19 and 7.78, respectively). Moreover, this study reported a gene-dose effect of *HLA-A*∗02:01, *DRB1∗15:01*, and the *PTPN22* variant for AC-DILI risk. A more than additive effect was validated for the co-occurrence of both *HLA-A*∗02:01 and *DRB1*∗15:01 risk alleles. Most importantly, this study calculated the genetic risk score and highlighted its utility in AC-DILI causality clinical assessment. By incorporating 5 (novel and previously known) markers, rs1363907, rs2476601*, HLA-*B∗15:18, *HLA-A*∗02:01, *HLA-DRB1*∗15:01, the genetic risk score was highly predictive of AC-DILI risk when cases were analyzed against both the general population and non-AC-DILI control cohorts. Although the risk score accounts for ∼13% of the AC-DILI susceptibility, which is one-third of the total (40%) of DILI risk that has been previously attributed to common genetic variants, it outperformed clinical risk factors. However, the genetic risk score was predictive in individuals of European descent only and was unlikely to be useful in African American and Hispanic populations. This could be attributed to the interpopulation variation in HLA alleles involved in AC-DILI pathogenesis. For instance, besides the known *HLA-DRB1*∗15:01 risk allele, *HLA-DRB1*∗15:03 and *HLA-DRB1*∗15:02 demonstrate strong enrichment in African American patients with AC-DILI.^288^ These data, together, pinpoint the usefulness of polygenic risk score in AC-DILI and underscore the need for additional large multiethnic studies.

In contrast to AC-DILI susceptibility, which is strongly associated with class II HLA alleles, DILI induced by flucloxacillin (an isoxazolyl penicillin *β*-lactam) is primarily HLA class I related. Flucloxacillin belongs to the penicillinase-resistant penicillins that are not hydrolyzed by staphylococcal enzymes; hence, it is mainly used to treat infections caused by penicillin-resistant staphylococci. Currently, with the emergence of methicillin-resistant staphylococcus strains, the use of flucloxacillin and other isoxazolyl penicillins has been largely impacted. Flucloxacillin is known to be a common cause of DILI, with an incidence of 8.5 per 100,000 (up to 110.5 per 100,000 in the elderly receiving ≥2 prescriptions).^27^ *HLA-B*∗57:01 genotype was found to be a key determinant of flucloxacillin-induced DILI, as it was present in 85% of patients (*P* = 8.7 × 10^−33^).^331^ *HLA-B*∗57:01-positive patients are 80 times more at risk of developing DILI after flucloxacillin treatment. Drug-specific, HLA-restricted T cells were also isolated from patients with a past flucloxacillin-DILI.^289^ Additionally, it was found that naive CD45RA^+^CD8^+^ T cells from donors expressing *HLA-B*∗57:01 were activated by flucloxacillin following binding to the drug presented by dendritic cells. Activation of CD8^+^ clones with flucloxacillin was restricted by *HLA-B*∗57:01 and the closely related *HLA-B*∗58:01.^289^ A recent study (*n* = 197 cases) further validated the *HLA-B∗57:01* allele as the main risk allele for flucloxacillin-DILI susceptibility (OR = 36.62; *P* = 2.67 × 10^−97^). The same study also reported a novel association with *HLA-B*∗57:03 (OR = 79.21; *P* = 1.2 × 10^−6^).^332^ Notably, no significant associations were found between *HLA-B*∗57 alleles and other isoxazolyl penicillins- or amoxicillin-induced DILI, and no significant signals were detected outside the HLA region for penicillin-related liver injury. The study also confirmed that approximately 20% of flucloxacillin-DILI cannot be predicted based on the carriage of this allele or any other known HLA allele.^332^ Collectively, despite the robust association of *HLA-B*∗57:01 with flucloxacillin-DILI, the clinical diagnostic or preventive utility of this allele remains impractical. This is primarily due to the low positive predictive value of *HLA-B∗57:01* testing (0.12%), as only ∼1–2 in every 1000 patients are likely to experience flucloxacillin-DILI in case of *HLA*-*B*∗57:01 positivity.^284^ Nonetheless, with the advancement in genotyping tools and the growing interest in genomics, patients’ genetic data is becoming more available in clinics. Prior knowledge of patients’ *HLA B*∗57:01 genotype may guide prescription options and improve antibiotic course outcomes.

The Swissmedic Label for flucloxacillin and HLA-B includes a precaution about the elevated risk of flucloxacillin-induced liver damage in patients carrying the *HLA-B*∗57:01; however, routine testing for this allele is not recommended because of its low predictive value.^333^ In addition, the DPWG guidelines regarding flucloxacillin and *HLA-B*∗57:01 positivity recommend monitoring the patient’s liver function regularly and choosing an alternative therapy if liver enzymes and/or bilirubin levels are elevated.^334^ In contrast, no CPIC guideline currently exists for flucloxacillin, and the FDA label does not mandate or recommend routine *HLA-B*∗57:01 testing before therapy. Supporting the limited clinical utility of pre-emptive screening, a nationwide cost-effectiveness analysis in the Netherlands (January–December 2019) estimated that *HLA-B*∗57:01 genotyping before flucloxacillin initiation is not cost-effective (positive predictive value ≈ 0.1%), whereas pretreatment testing for abacavir remains cost-saving because of its substantially higher positive predictive value (∼48%).^335^

Earlier in vitro studies have revealed that the formation of a toxic flucloxacillin metabolite (5′ hydroxyl methyl) is mediated by the CYP3A enzyme.^336^ Subsequent studies on primary human hepatocytes found that flucloxacillin is an agonist of the PXR and can promote the expression of CYP3A4, highlighting the role of PXR in flucloxacillin-induced DILI.^290^ Moreover, the frequency of *PXR* polymorphism rs3814055 (C-25385T) was higher among DILI cases compared with controls, both of which were under flucloxacillin therapy (OR = 3.37, *P* = .0023).^290^ Patients carrying the CC genotype of the *PXR*-25385 promoter region are at increased risk of flucloxacillin-DILI as this variant is associated with lower expression of PXR and CYP3A4, which may slow down flucloxacillin metabolism, leading to liver toxicity.^337^ Despite a plausible mechanistic link and supportive association data, the clinical utility of *PXR* rs3814055 testing for predicting flucloxacillin-induced liver injury remains unestablished.

Cephalosporins are another subgroup of *β*-lactams. Among the third-generation cephalosporins are cefotaxime and ceftriaxone, which have broad-spectrum bactericidal effectiveness against gram-positive and -negative bacteria. When given intravenously, ceftriaxone and cefotaxime can penetrate the blood–brain barrier and inhibit bacteria in the cerebrospinal fluid and, therefore, can be used to treat meningitis.^338^ OAT3 (*SLC22A8*) is a transporter expressed on the basolateral membrane of the proximal tubule and involved in the renal excretion of organic anions, including cephalosporins.^54^ A nonsynonymous Ile305Phe variant in the OAT3-encoding gene (*SLC22A8*) has been linked to cefotaxime renal clearance. This variant is rare globally, except in East Asia, where it is present in approximately 5.8% of the population (Genome Aggregation Database). In vitro kinetic studies on HEK293-Flp-In cells showed that the *OAT3*-Ile305Phe variant had reduced maximal cefotaxime transport activity. Cefotaxime renal clearance was significantly lower in healthy Asian volunteers heterozygous for the Ile305Phe variant compared with those homozygous for the wild-type allele.^54^ Nevertheless, only 5 cases were involved in this study, and therefore, these findings are exploratory and need further validation in larger clinical studies.

Genetic polymorphisms in the ABC subfamily C member 2 (*ABCC2*) and the ABC subfamily G member 2 (*ABCG2*) genes encoding multidrug resistance–associated protein 2 (MRP2) and breast cancer resistance protein, respectively, were linked to ceftriaxone concentration. Both transporters are involved in ceftriaxone efflux and are expressed in many tissues, including the blood–brain barrier, where they contribute to CNS protection from drugs and xenobiotics.^284^^,^^339^ In patients with signs and symptoms of CNS infections and receiving IV ceftriaxone, the *ABCC2* rs2273697 and the *ABCG2* rs13120400 variants were significantly associated with ceftriaxone exposure (*P* = .027 and .015, respectively). Patients carrying the GG genotype of rs2273697 had a lower ceftriaxone concentration in the cerebrospinal fluid than those with GA/AA genotypes. Moreover, a reduced ceftriaxone cerebrospinal fluid-to-plasma ratio was observed in rs13120400 C allele carriers when compared with TT carriers.^291^ These findings remain insufficient for clinical implementation. Additional validation and assessment of clinical impact are needed to determine whether these variants can meaningfully inform toxicity risk stratification and dosing strategies.

Drug-induced neutropenia is one of the idiosyncratic drug reaction complications that have been associated with the use of antibiotics. Many antibiotics can induce this adverse event; however, specific drugs are more frequently implicated, among which are the *β*-lactams.^340^ A literature analysis from 1968 to 2020 on *β*-lactam–induced neutropenia reported an incidence of approximately 10% following at least 2 weeks of intravenous therapy.^341^ Another report spanning the same period (1968–2020) analyzed all reported neutropenia cases (*n* = 83) and found that drug-induced neutropenia was most commonly associated with *β*-lactam (ceftaroline from cephalosporins) and glycopeptides (vancomycin).^342^ Similarly, a recent retrospective cohort study of patients who experienced antibiotic-induced neutropenia (absolute neutrophil count of ≤1.5  ×  10^9^/L) from 2016 to 2022, found that the most common intravenous antibiotic culprits are vancomycin (glycopeptide, 3.9%), followed by 2 *β*-lactams, ceftriaxone (2.0%) and cloxacillin (1.9%).^343^ Although this complication is reversible and resolves with therapy discontinuation, potentially severe complications can occur and can even lead to death.^342^

The exact mechanism underlying *β*-lactam-induced neutropenia is still not well understood, although host genetics has been proposed as a contributing factor. *β*-Lactam antibiotics are recognized substrates of both MRP4 and OAT3 transporters, and OAT3/*SlC22A8* knockout mice exhibit altered clearance and distribution of *β*-lactam antibiotics.^344^ Hahn et al^345^ investigated whether patients who experience neutropenia during prolonged *β*-lactam antibiotic course have genetic polymorphisms that impair the function of MRP4 or OAT1/3 in the kidney. Although not statistically significant, it was reported that patients homozygous for *MRP4* 3348 A to G variant exhibit a 5.3 times enhanced risk of developing neutropenia (*P* = .171). Conversely, a statistically significant association was observed between neutropenia and homozygosity for the wild-type allele at *MRP4* 3348 under standard or high *β*-lactam dosing (*P* = .03). These findings require validation in larger cohorts to confirm if there is a confident association.^345^ Importantly, PGx of *β*-lactam-induced neutropenia necessitates investigating genetic variants for each *β*-lactam class individually rather than studying host genetics for *β*-lactams as a group. It was found that using another *β*-lactam with a different side chain as an alternative therapy led to the successful recovery of the neutrophil count and reversed the neutropenia.^343^ This pinpoints the possible involvement of different genes/pathways that need further exploration. Most importantly, understanding the genetics behind *β*-lactam-induced neutropenia would allow for screening and identifying patients at risk before initiating a prolonged *β*-lactam antibiotic course. This would prevent further complications, including the patient’s susceptibility to opportunistic infections, prolonged hospitalization, or even death. Moreover, in severe cases, neutropenia may necessitate discontinuation of the antibiotic therapy, compromising the treatment efficacy.^346^ Overall, the PGx of *β*-lactam–induced neutropenia remains exploratory, and routine genetic screening cannot currently be recommended. In clinical practice, regular hematologic monitoring—typically around week 3 for non-vancomycin therapies and weekly for vancomycin—remains essential to enable early detection and appropriate modification or discontinuation of antibiotic therapy.^343^

##### c Sulfonamides

Sulfonamides (SN), or sulfanilamides, were the first synthetic antimicrobial drugs introduced to clinical use. They are broad-spectrum antibiotics used to treat gram-positive and gram-negative bacteria.^347^ Sulfonamides are competitive antagonists and structural analogs of p-aminobenzoic acid. They inhibit folic acid synthesis, which is important for bacterial DNA synthesis, consequently preventing the growth and proliferation of microorganisms.^348^ There are antibiotic and nonantibiotic SNs used for a wide range of conditions. Sulfamethoxazole (SMX), sulfamerazine, sulfamethizole, sulfamoxole, sulfamethazine, sulfisoxazole, and sulfapyridine are antibiotic sulfonamides.^347^ Although antibiotic SNs still play a role in treating certain infections, their use has significantly declined given the availability of more effective antibiotics. SMX, often combined with trimethoprim (TMP), is the only SN drug that is still largely prescribed for several conditions. It is cost-effective as treatment or prophylaxis in traveler’s diarrhea, chronic bronchitis, otitis media in pediatrics, urinary tract infections, shigellosis, *Pneumocystis jirovecii*, pneumonia/pneumocystis carinii pneumonia, and toxoplasmosis.^349^^,^^350^ Approximately 3%–8% of patients receiving SMX develop HSRs, ranging from mild/moderate eosinophilia to severe SJS/TEN.^304^ Several host- and drug-related factors seem to contribute to HSR to sulfonamides. SNs are metabolized by NAT1 and NAT2, producing N-acetylated metabolites, and genetic mutations in the *NAT2* gene particularly affect the acetylation rate. Slow acetylator genotypes might affect drug efficacy and toxicity. The involvement of the CYP2C9 monooxygenase system and the formation of hydroxylamine and nitroso compounds via spontaneous oxidation is also associated with enhanced toxicity.^347^ The interaction between hydroxylamine and nitroso with cellular DNA or proteins triggers HSR and tissue damage.^347^

It has been reported that the *NAT2* slow acetylator alleles ∗16, ∗5, ∗6, ∗7, and ∗14 are associated with a higher risk of SCAR in patients with AIDS treated with SMX-TMP (OR = 2.5, *P* = .02).^47^ A significant association between *NAT2* and SCAR was observed in patients with a null/null genotype of *GSTM1*, an enzyme of the GST family that contributes to the detoxification of sulfanilamide by the conjugation of its metabolites with glutathione.^47^ To date, approximately 50 *NAT2* alleles have been identified in the PharmVar database (as of July 2024), reflecting substantial functional diversity across ethnically distinct populations.^351^^,^^352^

The rapid acetylator genotype *NAT2*∗4 has been linked to a decreased risk of adverse reactions in children with pneumonia receiving SMX-TMP compared with those carrying the *NAT2*∗16 + ∗5 + ∗6 + ∗7 (*P* = .0024).^353^ Similarly, the *NAT2* variants, rs1799930 and rs1799931, have been associated with SMX–TMP adverse reactions (*P* = .02 and .01, respectively). More recently, a study in a Han Chinese cohort reported higher frequencies of slow acetylators among patients with SMX–TMP–induced liver injury, with rs1495741 (AA) and rs1041983 (TT) independently associated with increased risk (adjusted OR = 2.49, *P* = .001).^295^

Nonetheless, the overall evidence linking *NAT2* genotypes to SMX–TMP toxicity remains inconsistent across studies.^354^ This could be attributed to several factors, including the genotyping method, population ethnicity, sample size, and the presence of other underlying immunity-related disorders. *NAT2* is referenced in the FDA label for SMX–TMP as a potential contributor to variability in adverse reactions; however, the labeling is “informative” only and does not recommend genotype-guided prescribing.^355^ Consistently, the *NAT2*–SMX–TMP pair is classified as CPIC provisional, indicating that prescribing actionability based on genetics is not clear without further evidence review.

On the contrary, stronger evidence has accumulated regarding the association of HLA alleles and SMX-TMP-induced adverse events. *HLA-B*∗13:01, *HLA-B*∗15:02, and *HLA-B*∗38:02 have been associated with an increased risk of SJS and TEN in East Asian patients treated with SMX-TMP (OR = 15.2, 3.92–5.16, and 4.05; *P* = 7.2e-5, > .01, and .0259, respectively).^292^^,^^293^ These associations are classified under the level 2A clinical annotation of PharmGKB. These variants have a moderate level of evidence, but without formal prescribing guidelines. Additional HLA alleles show weaker association with elevated risk of TEN and SJS (level 2B), including *HLA-C*∗06:02, *HLA-C*∗07:27, and *HLA-C*∗08:01 (OR = 11.84, 27.73, and 3.53–5.71; *P* = .0131, .0259, and > .05, respectively).^292^^,^^293^

A multicountry case-control association study on 151 patients with cotrimoxazole (SMX and TMP)-induced SCAR and 4631 population controls from Taiwan, Thailand, and Malaysia, as well as 138 tolerant controls from Taiwan, replicated the strong association of *HLA-B*∗13:01 SMX-TMP-induced SCAR in Asians, with a high OR (OR = 40.1, *P* = 4.2 × 10^−23^).^294^ Moreover, in a recent meta-analysis involving 6 studies and 322 patients with SCARs, it was found that multiple *HLA-B* (∗13:01, ∗15:02, and ∗38:02), *HLA-C*∗08:01, and *HLA-A*∗11:01 alleles were significantly associated with sulfamethoxazole/cotrimoxazole (SMX/CTX)-induced SCARs.^356^

Beyond cutaneous reactions, pulmonary toxicity is one of the underrecognized and poorly understood adverse events associated with SMX-TMP.^357^ SMX–TMP–associated pulmonary toxicity has been linked to *HLA-B*∗07:02 and *HLA-C*∗07:02 (*P* = .000001 and .000018, respectively).^296^ SMX–TMP–induced liver injury has also been associated with HLA alleles. Particularly, *HLA-B*∗14:01 was found in 10% of European American patients with SMX-TMP DILI, which is 5.5-fold higher than in controls (*P* = .002).^297^ *HLA-B*∗35:01, on the other hand, has been identified as a potential genetic risk factor for SMX-TMP DILI among African Americans. *HLA-B*∗35:01 had a 2.8-fold higher frequency in cases than controls, with 50% of patients carrying this allele.^297^

Despite the strength and replication of several HLA associations, no major regulatory agency or expert consortium (eg, CPIC and DPWG) currently recommends routine pre-emptive HLA testing for SMX–TMP therapy. Accordingly, these variants are best considered "moderate evidence" pharmacogenetic signals with limited current clinical actionability. Further prospective and cost-effectiveness studies are required before genotype-guided implementation can be recommended.

##### d The macrolides

Macrolides are a widely prescribed class of broad-spectrum antibiotics used for treating local and systemic infections, including skin, respiratory, gastrointestinal, and genital tract infections. Their mechanism of action involves binding to the bacterial 50S ribosomal subunit and preventing the translation of mRNA, selectively inhibiting the translation of a subset of cellular proteins.^358^ Macrolides are comprised of a lactone ring with deoxy sugars attached, and they are subdivided into categories with different chemical structures. Erythromycin is the first macrolide to be approved by the FDA and is one of the most commonly used macrolides, in addition to clarithromycin and azithromycin.^359^

Macrolides are substrates for apically polarized efflux transporters, such as P-gp (*ABCB1* gene), which can potentially restrict intestinal absorption and mediate their excretion into bile.^360^ Because of differences in their chemical properties, erythromycin and clarithromycin exhibit more extensive interactions with drug transporters and metabolizing enzymes compared with azithromycin. Macrolides are widely distributed in the blood and tissues, and they preferentially bind to *α*-1-acid glycoprotein, encoded by the *ORM1* gene.^359^ Erythromycin and clarithromycin are also substrates for OATPs, particularly OATP1B1 and OATP1B3 (encoded by *SLCO1B1* and *SLCO1B3* genes), which mediate their uptake and increase absorption.^360^ Besides being substrates for the active carriers, OATPs and P-gp, erythromycin and clarithromycin are both extensively metabolized by CYP3A4 in the liver.^361^ On the other hand, azithromycin does not interact with OATP1B1 and OATP1B3 and has been identified as a weak substrate for CYP3A4.^359^ Both P-gp and MRP2 transporters (encoded by *ABCC2*) are involved in the excretion of azithromycin.^362^

Genetic variants in the *ABCB1* and *ABCC2* genes have been associated with macrolides’ transport and clearance. In a small study of healthy Chinese Han individuals, carriers of the 2677GG (rs2032582) and 3435CC (rs1045642) diplotypes in *ABCB1* had elevated maximum concentrations of azithromycin compared to individuals with the *2677TT/3435TT* diplotypes.^298^ Another small-scale study (*n* = 16) on healthy volunteers from Pakistan reported that *ABCB1* genotypes and gender significantly influence the PKs of azithromycin.^363^ However, these findings are deriven from limited sample sizes and have not been consistently replicated.

For erythromycin, homozygosity for the reduced-function *ABCC2* rs717620 TT genotype has been associated with increased erythromycin metabolism compared with CC and CT carriers.^299^ In addition to *ABCC2*, variations in the *SLCO1B1* gene have been implicated in the modulation of erythromycin metabolism. A 50% reduction in the transport of erythromycin was observed in cells expressing OATP1B1∗5 (V174A), in comparison to OATP1B1∗1A (wild-type).^56^ Moreover, deficiency of the ortholog transporter Oatp1b2 in mice resulted in a 52% reduction in the metabolic rate of erythromycin (*P* = .000043). In the same way, individuals carrying the rs4149056 (c.521T>C) variant in the *SLCO1B1* gene, which encodes OATP1B1∗5, exhibited a decrease in erythromycin metabolism (*P* = .0072).^56^ In this study, the overall impact of the OATP1B1∗5 mutation on explaining variation in erythromycin metabolism was only ∼10.6%, suggesting the contribution of additional genetic and nongenetic factors.

Although not well explored, the interethnic variation in macrolide metabolism and concentration could reflect population genetic differences in *CYP3A4* and *ORM1* genes. It has been reported that Asians have lower CYP3A4 activity compared with Caucasians.^364^ At the same time, after a single oral dose of erythromycin, Koreans had a 65% higher area under the concentration–time curve (AUC∞) compared with Caucasians. Caucasians, on the other hand, exhibited a lower area under the concentration–time curve and lower half-life for azithromycin compared with Mexican, Thai, Chinese, Japanese, and Jordanian patients.^365^ Additionally, Caucasians have been shown to have 10%–20% less *α*-1-acid glycoprotein concentration compared with individuals of Asian, Iranian, and African descent, potentially suggesting the effect of distinct population genetic structure.^359^^,^^365^

Collectively, although variants in *ABCB1*, *ABCC2*, and *SLCO1B1* demonstrate biologically plausible effects on macrolide PKs, the available evidence is derived largely from small PK studies with limited replication and no supporting CPIC, DPWG, or FDA pharmacogenetic prescribing recommendations. Accordingly, these associations are currently considered to have insufficient evidence for clinical implementation and require validation in larger studies incorporating clinically meaningful outcomes before genotype-guided macrolide therapy can be recommended.

#### 2 Antituberculosis drugs

The first-line chemotherapy course for drug-susceptible TB includes multidrug combination therapy as recommended by the WHO.^366^ The 6-month regimen (2HRZE/4HR) is the standard for treating TB, and it comprises 2 months of isoniazid (isonicotinic acid hydrazide [INH]), rifampicin (RIF), ethambutol, and pyrazinamide (PZA), and 4 months of INH and RIF. The shorter course includes a 2-month regimen of INH, RIF, moxifloxacin, and PZA, followed by 2 months of INH, RIF, and moxifloxacin (2HPMZ/2HPM). Although these regimens have remarkably reduced the TB burden and recorded a treatment success rate of 85%, drug resistance and drug-induced adverse events represent a serious concern.^366^

Adverse events occur in 25% to 75% of patients receiving anti-TB drugs.^367^ These include gastrointestinal symptoms, skin rashes, hepatitis, optic neuropathies, and other manifestations.^368^ Anti-TB drug-induced hepatotoxicity (ATDH) is the most serious and potentially fatal adverse event requiring drug discontinuation.^369^ The reported incidence of ATDH ranges from 2% to 28% and varies widely.^370^ Many risk factors are associated with ATDH including old age (above 60 years), female sex, slow acetylator status, poor nutrition, HIV status, drug regimens, and pre-existing hepatitis.^370^^,^^371^ In most ATDH cases, liver injury is manifested as a mild asymptomatic elevation in liver enzymes that resolves with time. However, approximately 1% of treated patients experience severe liver injury, characterized by prolonged transaminase increase and hepatocellular damage that can lead to fulminant liver failure and death in rare cases.^48^^,^^370^ Among the first-line anti-TB drugs INH, RIF, and PZA, which can all cause hepatotoxicity, most cases of critical ATDH appear to be because of INH, whether administered as monotherapy or part of multidrug regimens. The withdrawal of anti-TB drugs remains the cornerstone for managing ATDH. Close monitoring of alanine aminotransferase levels is required until normalization. Subsequently, rechallenge can be considered. Reintroduction approaches can be sequential with incremental dosing, sequential with full dosages, or simultaneous with full dosages. The sequential approach is useful for identifying the causative agent and adjusting the anti-TB treatment plan if necessary.^372^

Genetic variation in genes involved in INH metabolism has been associated with *INH-induced DILI*, particularly the slow acetylator variants of the *NAT2* gene. INH metabolism primarily involves NAT2-mediated acetylation and cytochrome P450-dependent oxidation pathways, which together generate several metabolites implicated in drug efficacy and toxicity.^373^ The role of the acetylation status associated with the *NAT2* gene variation and the impact on ATDH has been extensively studied. In the PharmGKB database alone, approximately 50 studies reported statistically significant associations between *NAT2* and anti-TB drug metabolism or induced toxicity, most of which were related to INH. Patients with *NAT2*∗1/∗1, ∗4/∗4, or ∗1/∗4 haplotypes were reported to have increased metabolism of INH compared to those carrying ∗5, ∗6, ∗7, ∗14, ∗16, or ∗39 allele combinations with or without ∗1 and ∗4. In line with that, patients with TB carrying any 2 alleles of the *NAT2*∗5, ∗6, ∗7, ∗14, or ∗16 were found to have a higher risk of developing hepatotoxicity when treated with isoniazid-containing regimens compared with patients carrying at least one copy of the *NAT2*∗1 or ∗4 alleles.^374^ Notably, the risk of hepatotoxicity associated with certain alleles reached up to 9-fold increase, as reported with *NAT2∗6* and ∗6/∗7 alleles in 2 studies.^300^^,^^301^ In a meta-analysis that analyzed data from 41 articles (39 distinct cohorts, receiving at least one of the following drugs: INH, RIF, PZA, or ethambutol), it was concluded that slow/intermediate *NAT2* acetylators were significantly more likely to develop hepatotoxicity compared with rapid acetylators (OR = 1.59).^375^ However, despite the considerable evidence of this association, there are few other reports that did not find any correlation between *NAT2* slow acetylators and anti-TB-induced DILI.^376^^,^^377^ This could be because of ethnic differences in *NAT2* allele frequencies, especially that most of the current findings were obtained from Eastern Asian populations, except for a few studies from other ethnicities.

Together with *NAT2*, genetic variations in other genes have also been associated with anti-TB drug hepatotoxicity. This includes the *CYP2E1* gene, which encodes an enzyme involved in the metabolism of INH, acetylhydrazine, and hydrazine to potentially hepatotoxic intermediates.^378^ As an essential phase II detoxification enzyme, the gene encoding GSTM1 also seems to harbor variants that correlate with liver toxicity. Although the current data in the field are complex and discordant, meta-analyses have helped re-evaluate the contradicting findings. A significant association, albeit with a lower OR than that of *NAT2*, has been found between *CYP2E1*∗1A/∗1A and *GSTM1* null/null genotypes and ATDH.^48^ It is worth noting that these associations were robust among East Asians but could not be replicated in Caucasians, despite some studies indicating positive correlations.^48^

Additional candidate loci, including *XPO1* (rs4430924, AA), *RIPOR2* (rs10946737, AA+AG), *HLA-B*∗52:01, *HLA-DPB1*∗05:01, *NOS2A* (rs11080344, CC), *BACH1* (rs2070401, CC), and *MAFK* (rs4720833, GA or AA)^379^, ^380^, ^381^, ^382^, ^383^ have been linked to ATDH risk. Variants in *NR1I2* have been associated with rifampicin exposure, including the rs3732357 (GA/AA) genotype of the *NR1I2* gene, which was recently reported to be associated with significantly lower RIF plasma exposure in comparison to the GG genotype.^384^ However, most of these findings require replication and/or validation in larger and more diverse cohorts. It also remains mechanistically unclear how these variants may be associated with ATDH.

Rather than a single gene, multiple genetic variations likely affect the response to INH and correlate with drug-induced hepatotoxicity. These genetic factors may lead to varying levels of toxic metabolites produced from numerous pathways. The poor concordance between different studies, especially for *NAT2*, *CYP2E1*, and *GSTT1* genes, suggests the involvement of multiple genetic and nongenetic factors in the ATDH process. Identifying these factors and integrating more genotype/phenotype associations could lead to further progress in therapeutic efficacy and cost reduction during TB treatment.

Importantly, no major pharmacogenetic consortium currently recommends routine *NAT2* genotyping before INH therapy. Although *NAT2* haplotypes had strong (1B) and moderate (2A) levels of association with anti-TB toxicity and metabolism as determined by the PharmGKB scoring system, respectively, the current FDA labeling for INH-containing products is classified as informative, noting variability in acetylation and potential toxicity risk without recommending genotype-guided dosing.^385^ Similarly, the Japanese PMDA label acknowledges interindividual variability in acetylation speed but does not mandate testing.^386^ This regulatory recognition of *NAT2* variability reinforces the potential of *NAT2* genotype-guided dosing, which has been shown in clinical trials to reduce unfavorable adverse events without compromising therapeutic effectiveness.^387^^,^^388^ Although promising, these findings require further confirmation across diverse populations and real-world settings before widespread clinical adoption can be recommended.

## V Pharmacogenomics of antibacterial and antiviral drugs in clinical practice

Moving PGx from basic research to clinical guidelines and recommendations is a significant step that requires robust, high-level evidence. In the last 5 years, advancements in the field of PGx have boosted its clinical utility. Single drug–gene testing, introduced into the clinical practice approximately 20 years ago, continues to be the most commonly applied example of PGx testing. This covers gene–drug pairs with a high level of evidence, such as *HLA-B*∗57:01 testing for abacavir hypersensitivity and dihydropyrimidine dehydrogenase testing for fluoropyrimidine toxicity.^389^

More recently, PGx testing strategies have evolved from the reactive/diagnostic single-gene testing to investigate the cause of ADRs to proactive/pretherapeutic testing.^390^ A better-informed approach is the use of preemptive, panel-based PGx testing, which covers a set of clinically actionable variants for commonly prescribed drugs. Two main types of panel-based PGx testing are currently available: disease-oriented panels, which include genes associated with drugs relevant to a particular disease, and broad panels covering all PGx variants from clinically actionable guidelines.^390^

Recent implementation studies and programs have provided evidence supporting the benefit of PGx testing in clinical settings. For instance, in a large multicenter study (NCT03093818) involving almost 7000 patients in 7 European countries, the implementation of a 12-gene (50 variants) PGx panel reduced ADRs by approximately 30%.^391^ Another study (NCT02297126) evaluated the impact of prospective 13-gene (43 variants) panel testing in 2612 patients on the occurrence of ADRs.^392^ Although statistically nonsignificant, this study reported a reduction in serious ADRs in patients who underwent pharmacogenotyping. Whether using a broad PGx panel or designing a disease-oriented panel tailored for IDs, emerging studies and clinical trials highlight the promise of pretherapeutic PGx testing in reducing ADRs. The design of such panels can be population-specific and guided by recommendation from the working groups and regulatory agencies such as CPIC, DPWG, FDA, and others.

The CPIC has led extraordinary efforts to provide peer-reviewed, evidence-based, updatable, and detailed gene-drug clinical practice guidelines. As of early 2026, CPIC has issued ∼35 clinical guidelines and catalogued 316 Final and 257 Provisional gene–drug pairs across more than 100 unique genes. CPIC publishes guidelines to help clinicians understand how genetic test results can be used to optimize drug therapy. However, these guidelines should be readily accessible at the point of care for clinicians, practitioners, and pharmacists. The method by which CPIC assigns levels to gene–drug pairs is based on PharmGKB annotation levels of evidence (1A, 1B, 2A, and 2B), FDA-approved drug labels, or nomination to CPIC for consideration. Gene–drug pairs initially receive provisional status and are upgraded to final status following full guideline development. Only level A and B pairs currently support genotype-guided prescribing.

Out of 573 gene–drug pairs (as of February 1, 2026) assessed by the CPIC, 52 genes were related to anti-infective drugs. Of these, 38 were specifically related to antiviral and antibacterial drugs, out of which 20 ranked among the top pairs in CPIC (A or B) and/or PharmGKB (1A, 1B, 2A, or 2B) and/or have an FDA-approved drug label (testing required or actionable PGx). Table 6 summarizes all 20 gene–drug pairs with the levels of evidence and the drug label annotations of the FDA and other drug regulatory agencies: the EMA, HCSC, Swiss Agency for Therapeutic Products (Swissmedic), and the PMDA.

**Table 6:** Top gene–drug associations and pharmacogenetic information from drug regulatory agencies

Drug Class	Class	Drug	Gene	CPIC Level	Available Guidelines	PharmGKB, LoE	Available Drug Labels	FDA Label Summary
Antibiotics	AG	Amikacin Gentamicin Streptomycin Tobramycin Plazomicin Neomycin Kanamycin Netilmicin Paromomycin Ribostamycin	*MT-RNR1* *MT-RNR1* *MT-RNR1* *MT-RNR1* *MT-RNR1* *MT-RNR1* *MT-RNR1* *MT-RNR1* *MT-RNR1* *MT-RNR1*	A A A A A A A A A A	CPIC CPIC CPIC CPIC CPIC CPIC CPIC CPIC CPIC CPIC	1A 1A 1A 1A — 3 1A 1A 1A	Actionable PGx (FDA) Actionable PGx (FDA) Actionable PGx (FDA) Actionable PGx (FDA, HCSC) Actionable PGx (FDA) Actionable PGx (FDA) — — — —	Amikacin, gentamicin, streptomycin, tobramycin, plazomicin, and neomycin Ototoxicity was observed with AGs in MT-RNR1 m.1555A>G variant carriers. In case of maternal history of ototoxicity-related AG or mitochondrial DNA variant in the patient, consider alternatives unless the increased risk of permanent hearing loss is outweighed by the severity of the infection and lack of safe and effective alternative therapies. — — — —
	Nitrofuran	Nitrofurantoin	*G6PD*	A	CPIC	3	Actionable PGx (FDA, HCSC, Swissmedic)	Risk of hemolytic anemia with nitrofurantoin in patients with G6PD-deficient
	Quinolone	Nalidixic acid	*G6PD*	C	CPIC	—	Actionable PGx (FDA, PMDA)	Risk of hemolytic anemia with nalidixic acid in patients with G6PD-deficient
	Sulfone	Dapsone	*G6PD*	A	CPIC	4	Actionable PGx (FDA, HCSC, PMDA)	Oral dapsone treatment can result in hemolysis and hemolytic anemia, particularly in patients with G6PD-deficient
			*HLA-B*	C	—	2A	—	—
	Anti-TB	Isoniazid	*NAT2*	C	—	1B	Informative PGx (FDA, PMDA)	The rate of acetylation does not significantly alter the effectiveness of isoniazid. However, slow acetylation may lead to higher blood levels of the drug and, thus, to an increase in toxic reactions. No specific genes mentioned.
Antiviral	Anti-HIV	Abacavir	*HLA-B*	A	CPIC	1A	Testing required (FDA, EMA, HCSC, Swissmedic) Informative PGx (PMDA)	Risk of HSRs. Genetic testing for the *HLA-B*∗5701 allele is required before initiating or reinitiating abacavir.
		EFV	*CYP2B6*	A	CPIC and DPWG	1A	Informative PGx (FDA, EMA, HCSC)Actionable PGx. (Swissmedic, PMDA)	*CYP2B6*∗6/∗6 genotype carriers have higher EFV concentrations compared with the ∗1/∗1 genotype. Increased EFV concentrations correlate with QTc prolongation and late-onset neurotoxicity.
		Dolutegravir	*UGT1A1*	B/C	—	—	Informative PGx (FDA, EMA, HCSC, Swissmedic)	Subjects with poor metabolizer *UGT1A1* genotypes have decreased dolutegravir clearance and increased AUC. No specific *UGT1A1* variants or genetic testing.
		NVP	*CYP2B6*	B/C	—	2A	—	—
			*HLA-DRB1*	C	—	2B	—	—
		ATV	*UGT1A1*	A	CPIC and DPWG	1A	—	—
	Anti-HCV	PEG-IFN*α*2b	*IFNL3*	A	CPIC	1A	Informative PGx (FDA)	*IL-28B* rs12979860-CC was associated with variable SVR rates: CC 66% vs CT 30% vs TT 22%.

AUC, area under the concentration–time curve; LoE, level of evidence.

Among anti-infective examples, *HLA-B*∗57:01 testing before abacavir therapy remains the most clearly established case of mandatory pharmacogenetic screening, as recommended by the FDA, EMA, HCSC, and Swissmedic. However, abacavir-*HLA-B* is considered an “Informative PGx” according to the PMDA package insert for abacavir. Despite the strong association between *HLA-B*∗57:01 and HSRs to abacavir in multiple populations, this association is unknown in Japanese patients, and the ∗57:01 allele has a prevalence of only 0.1% in the Japanese population.^393^

Most other gene–drug associations are categorized as clinically actionable or informative, although a few inconsistencies between drug regulatory agencies have been noted. The “Actionable PGx” label indicates information on changes in efficacy, metabolism or dosage adjustment, or toxicity. For example, *MT-RNR1* variants associated with AG-induced ototoxicity (actionable PGx) are recognized in multiple regulatory labels and supported by high-level evidence; however, routine pretreatment screening is not consistently performed in many clinical settings. Similarly, *CYP2B6*-guided EFV dosing is supported by CPIC and/or DPWG recommendations, however, regulatory agencies recommendations and real-world uptake remains heterogeneous. Other associations, such as *NAT2* with SMX-TMP, *NAT2* with isoniazid, and several transporter polymorphisms affecting antiviral and antibiotic PK, currently remain primarily informative or investigational from a regulatory standpoint. Many of these associations are biologically plausible and supported by observational studies, but still lack consistent prospective validation demonstrating clear clinical utility or cost-effectiveness. Consequently, routine genotype-guided prescribing is not presently recommended.

Overall, the clinical integration of PGx in IDs remains selective and drug-dependent. This could be because of the complexity of gene–drug–pathogen interactions, and for many anti-infective agents, currently available data do not support a clinically meaningful impact of host genetic variation. Continued well designed studies across diverse populations will help distinguish true biological neutrality from areas where additional evidence may still refine clinical utility. Systematic evaluation of the clinical benefit of PGx testing may enable broader and more consistent incorporation into clinical practice recommendations, ultimately improving patient care.^394^

The association between abacavir hypersensitivity and the *HLA-B*∗57:01 allele represents one of the most successful examples of PGx implementation in the management of IDs. This model offers a blueprint that can be studied to extract lessons for broader PGx clinical implementation. Several factors contributed to the clinical success of the abacavir-*HLA-B*∗57:01 case. These include the reproducible association between *HLA-B*∗57:01 and abacavir HSR, the availability of large-scale randomized clinical trials, the well characterized mechanistic insight (immune-mediated), the wide accessibility, affordability, and reliability of *HLA-B*∗57:01 genotyping, and the regulatory support from different organizations such as the FDA, HCSC, and EMA. In addition, this allele particularly has an exceptionally high negative predictive value (100%), thereby making it highly reliable and valuable for clinical decision-making, a key reason why the *HLA-B*∗57:01 test stands as one of the most successful examples of PGx implementation in the management of IDs.

Building upon this model, future PGx efforts in IDs should focus on the replicability of genetic associations across diverse populations, infrastructure development to support cost-effective and scalable genotyping, and seamless integration of genetic testing into routine clinical workflows supported by clear evidence-based guidelines. Although the collective scientific efforts have greatly benefited the treatment of specific IDs, for the majority of IDs, our knowledge of PGx and its contribution to drug response remains mosaic and incomplete, limiting its translational potential.

## VI Population diversity in pharmacogenomics of infectious diseases

### A Interpopulation differences in infectious diseases-related pharmacogenes

Representing global populations in exploring the PGx of IDs is crucial for developing effective treatments. Global research has identified numerous genetic variants linked to anti-infective drug response and potential ADRs. However, many of these studies are inconsistent or involve small, population-specific samples, limiting their statistical significance and generalizability. To help address these data gaps, specialized resources such as the PharmFreq database have been developed to provide curated, high-quality pharmacogene allele frequencies for numerous global populations, complementing the data available in broader databases such as PharmGKB.^395^

A large diversity was observed in PGx studies on important pharmacogenes that interact with anti-infective agents. For instance, the allelic frequency of *HLA-B*∗57:01, which has been robustly associated with abacavir HSR in patients with HIV, varies widely among different populations. Generally, approximately 94% of patients are at low risk of abacavir-HSR (*HLA-B*∗57:01 negative), whereas approximately 6% are at high risk due to carrying at least one copy of the *HLA-B*∗57:01 allele (*HLA-B*∗57:01 positive). The prevalence of the *HLA-B*∗57:01 allele is very rare or virtually absent in some African and Japanese populations, whereas it is more common in Europeans and South Asians.^115^ Moreover, this allele shows low to moderate frequency among Latinos (1.39%), African Americans (2%–3%), and Afro-Caribbeans (∼0.61%).^396^

Variability in *HLA-B*∗57:01 frequency is observed not only between global populations but also within populations. For instance, among Africans specifically, *HLA-B*∗57:01 frequency varies from 0% in the Nigerian Yoruba to 3.3% in the Kenyan Luhya and up to 13.6% in the Kenyan Maasai.^397^ Similarly, in Latin America, the frequency of the *HLA-B*∗57:01 allele demonstrates marked regional and ethnic variability. In Brazil, allele frequency ranges from 0.005 to 0.026 in the Southeast region to approximately 0.03 among the Puyanawa people.^398^ In Chile, the frequency of the *HLA-B*∗57:01 allele was found to be 1.1% among HIV-positive individuals and 1.8% in the general population.^399^ In Argentina, the *HLA-B*∗57:01 allele is found in 4.9%, as reported from a large cohort involving 1646 HIV-positive individuals. A comparable frequency has also been reported among healthy individuals in Costa Rica (5%).^396^^,^^400^ In contrast, lower frequency was reported from Mexico, where Mexican mestizos exhibited an overall allele frequency of 1%.^401^ Colombia shows regional variability, with an overall prevalence of 2.7% in Colombian patients with HIV, compared with 11.4% in the Caldas region, where Caucasians and mestizos predominate, and remaining approximately 6% in central regions.^125^

Given that HLA genes are among the most polymorphic in the human genome, other HLA alleles linked to different anti-infective drugs are also distributed variably across different populations. This includes *HLA-A*∗30:02, which is associated with liver injury induced by AC, and has a frequency that varies from 0% in Thai to approximately 5% in African Americans.^402^ *HLA-C*∗08:01, which is linked to cotrimoxazole (SMX and TMP)-induced SJS/TEN, exhibits allelic frequencies varying from 0% in Caucasians, to 2.41% in North Americans, and up to 10.3% in the Thai population.^402^ Importantly, understanding ethnic differences in HLA is crucial for the clinical implementation of PGx in anti-infective therapy. This will not only help prevent severe ADRs, but also inform vaccine development, disease risk assessment, and ultimately improve population health outcomes.

Another important pharmacogenes that also exhibits significant genetic variability is *CYP2B6*. The *CYP2B6*∗6 allele shows distinct intraethnic and interethnic differences with minor allele frequency ranging from 0.33 to 0.5 in African and African American populations, 0.10 to 0.21 in Asians, and 0.14 to 0.27 in Caucasians.^403^, ^404^, ^405^ Substantial differences were observed within African populations, as *CYP2B6*∗6 ranges from 42% in the Nigerian Yoruba population to 34% in the Kenyan Kikuyu and 22% in the Tswana population of Botswana.^405^ Similarly, the *CYP2B6*∗9 variant frequency varies significantly across Africans, ranging from 20% in the South African Xhosa to 22% in Botswana, 25.9% in Ugandan Bantus, 34% in Kenya, 36% in South Africa, 37% in Cameroonians, 40% in Zimbabweans of San descent, and up to 55% in the Congolese.^404^^,^^406^^,^^407^ Notably, African patients, who exhibit the highest prevalence of *CYP2B6*∗6 variant can actually benefit from a 35% reduction in EFV dosage, highlighting its clinical significance. In European populations, the *CYP2B6*∗6 allele is relatively common, with a prevalence of 32.1% in Germans, 24.8% in the Swiss, and 28.15% in the British.^404^ In contrast, *CYP2B6*∗6 is relatively lower among East Asians, ranging from 16.4% in Japanese, 16.4% in Koreans, and 18.4% in Han Chinese. South Asian populations also exhibit variability, with frequencies of 18.5% in Indians and 41.7% in Indonesians.^404^

Previous studies have reported associations between *NAT2* gene variation and the PK of isoniazid. Important SNPs such as *NAT2*∗4 are more prevalent in East Asians, whereas alleles associated with slow acetylation are more common in other populations. This includes *NAT2*∗5, which is found in 50%–60% of Europeans, and *NAT2*∗6 that ranges between 40% and 50% in South Asians. On the other hand, African populations have higher frequencies of *NAT2*∗7, with a frequency of 10%–20%. The frequency of *NAT2*∗7 in other frequencies ranges from 0.22% in Amish, 2.05% in Ashkenazi Jewish individuals, 2.57% in non-Finnish Europeans, 3.31% in African Americans, 3.4% in Finnish individuals, 7.39% in South Asians, 9.58% in Latino or admixed Americans, and reaching the highest frequency of 15.09% in East Asians. The frequency of the *NAT2*∗6 allele shows notable variation across populations. It is highest in South Asians (36.59%), followed by Ashkenazi Jews (36.15%), and the Amish (35.31%). Among non-Finnish Europeans, *NAT2*∗6 is found in 29.21%, whereas it is slightly lower in African Americans (25.87%), East Asians (25.61%), and Finnish individuals (23.41%). The lowest frequency is observed among Latino or admixed American populations (20.71%). Similarly, the distribution of the *NAT2*∗5 allele also varies across populations. It is most frequent in Finnish and non-Finnish Europeans (46.91% and 44.69%, respectively). The frequency is slightly lower in Amish (36.07%), Latino or admixed Americans (35.24%), and African Americans (30.49%). South Asians exhibit a frequency of 33.83%, whereas in East Asians, the frequency is notably lower at 3.65% (PharmGKB).

Another important variant, rs12979860-CC (*IL-28B*), which is strongly associated with antihepatitis response (PEG-IFN/RBV), exhibits variable frequencies between East Asia (92.91%) and South Asia (77.36%). Moreover, the frequency of the rs12979860 (*IL-28B*) C allele is approximately 70% in northern Europeans and approximately 30% in African populations.^214^

The gene encoding UGT1A1, which is essential for the glucuronidation and elimination of bilirubin and is associated with ATV, also showed considerable ethnic variability. *UGT1A1*∗28 allele is the most prevalent variant of the *UGT1A1* gene and is particularly prevalent among individuals of African descent. It occurs at frequency of 0.42–0.56 in African Americans, 0.26–0.31 in Caucasians, and as low as 0.09–0.16 in Asian populations.^408^

Together, these findings highlight the clinical relevance of population-specific genetic variation in key pharmacogenes, including *HLA-B, HLA-C*, *CYP2B6*, *NAT2*, *IL-28B*, and *UGT1A1*, in shaping drug response and toxicity in IDs. However, despite this growing body of evidence, PGx data remain unevenly distributed across global populations, underscoring the need to address underrepresentation in current research.

### B Pharmacogenomics of infectious diseases in underrepresented populations

PGx research in underrepresented populations has become increasingly significant because of the unique genetic diversity shaped by historical population movements and cultural interactions. This distinct genetic composition affects how individuals respond to medications, influencing both effectiveness and the risk of adverse reactions. Researchers focus on understanding genetic factors that impact drug response, aiming to enhance healthcare outcomes in these populations. However, such PGx-based practices are underdeveloped in many regions, such as the Middle East, primarily because of the lack of sufficient data on pharmacogenomic variations in local populations.

Over the last decade, multiple previously underrepresented countries have taken significant steps toward building genome-sequencing capacity, enabling the initiation of population genomics and genetics investigations, including PGx research. For instance, a major advancement in PGx in Qatar is the Qatar Genome Program, which was launched in 2015. This large-scale initiative is creating a comprehensive database that integrates whole genome sequencing with omics and phenotypic data, sourced through the Qatar Biobank. The Qatar Biobank collects and preserves biological samples and health information from both Qatari citizens and residents.^409^ By focusing on genetic data specific to the Qatari population, the Qatar Genome Program enables researchers to identify genetic markers associated with disease risk, leading to earlier diagnoses and personalized disease management. Additionally, these data support the development of drug response predictors tailored to the genetic profiles of Qataris.^410^ Qatar’s population is characterized by significant genetic diversity, encompassing key Arab ancestral lineages: Qahtanite (Peninsular Arabs) and Adnanite (General Arabs and West Eurasian Arabs).^411^ Principal component analysis of genome-wide genotype data from Qatar has identified 3 distinct genotype clusters, reflecting an Arabian origin, an Eastern or Persian origin, and an African admixture.^412^

Another initiative is the Catalogue of Transmission Genetics in Arabs, based in the United Arab Emirates. This project aims to create an extensive database documenting genetic diseases prevalent among Arab populations, including those in the United Arab Emirates. The Catalogue of Transmission Genetics in Arabs is an important component of broader Arab genomic research efforts.^413^ Furthermore, the Saudi Human Genome Program, based in the Kingdom of Saudi Arabia, focuses on studying genetic diseases in patients from the Gulf region.^414^ Additionally, the Centre of Excellence in Genomic Medicine Research, located at King Abdulaziz University in Jeddah, Kingdom of Saudi Arabia, is a recognized center specializing in personalized medicine.^415^

Several pharmacogenomic studies have been conducted on the Qatari population for the identification of genetic variants associated with response to various drugs. For example, studies identified that the *HLA-B*∗57:01 allele has a prevalence of 2.72% in Qatar.^138^ In another study, the analysis of 6045 whole genomes from Qatar identified that the presence of *HLA-B*∗57:01 genotypes predicted a high risk of abacavir hypersensitivity in 2.6% of the population.^416^

Similar studies from Iran, Morocco, Jordan, and Saudi Arabia also reported variable prevalences of important PGx-related factors affecting anti-infective drug response. In healthy individuals, the reported rates of *HLA-B*∗57:01 were 4.1% in Morocco and 1% in Jordan.^417^ Moreover, a 3.0% prevalence of the *HLA-B*∗57:01 allele was reported in Iranian patients with HIV-positive.^139^ Additionally, a study on Saudi population reported that the observed prevalence of *HLA-B*∗57:01 among Saudi patients with HIV was relatively low (1.59%), underscoring the importance of genetic screening in identifying individuals at risk of HSRs to abacavir.^137^ Moreover, another study reported that 2.1% of Kuwaiti individuals carry the *HLA-B*∗57:01 allele.^418^

Other studies investigated the frequency of the *CYP2B6* variants. Mardi et al, reported that the frequency of the *CYP2B6*∗5 variant is notably higher among Kurds compared with other Iranian ethnic groups. In fact, the elevated prevalence of both *CYP2B6*∗6 and ∗5 variants in Kurds places this ethnic group at greater risk of suboptimal drug exposure and ADRs. Consequently, prioritizing *CYP2B6* variant testing in the Kurdish population is recommended. Another study assessed the frequencies of *CYP2B6∗*6, *CYP2B6*∗4, and *CYP2B6*∗5 polymorphisms across 3 Iranian ethnicities, finding significantly higher allele frequencies for *CYP2B6*∗6 and *CYP2B6*∗5 in Kurds compared with other Iranians. These findings suggest that Kurds may face increased risks of ADRs and suboptimal anti-HIV responses.^419^ Generally, the frequency of *CYP2B6*∗6 allele is notably higher in certain Middle Eastern populations, such as Iranian Mazani, where it reaches 41.50%, and Iranian Baloch at 48%. The *CYP2B6*∗4 allele shows similar patterns with high frequencies in Iranian Baloch at 43% and Iranian Mazani at 37.41%. On the other hand, the *CYP2B6*∗5 allele exhibits lower frequencies, reaching 24.49% in the Iranian Mazani population and only 0.08% in Iranian Baloch individuals.^420^, ^421^, ^422^ These relatively low frequencies of *CYP2B6*∗5 across Middle Eastern countries indicate that rapid metabolism of drugs such as EFV may be less common in these populations compared with others with higher frequencies of the allele.

Underrepresentation in PGx studies extends beyond the Middle East to populations in Africa and Asia, Hispanic or Latin Americans and others, where high genetic diversity remains insufficiently captured in current datasets. Genetic variation across populations can substantially influence drug efficacy and toxicity, as illustrated in previous reviews.^423^, ^424^, ^425^ For instance, variants in genes such as *CYP2C9*, *VKORC1*, and *CYP4F2* explain a significant proportion of warfarin dose variability in European populations but account for considerably less variability in individuals of African ancestry, limiting the transferability of dosing algorithms across populations. A similar population-specific risk is observed with G6PD deficiency, which is highly prevalent in malaria-endemic regions (reaching up to 25%). Individuals with this deficiency are particularly susceptible to hemolytic toxicity when exposed to certain anti-infective agents. Failure to account for population-specific *G6PD* variants led to the withdrawal of the chlorproguanil-dapsone combination in 2008, despite its efficacy, because of severe hemolytic toxicity in deficient individuals.^426^

PGx research in underrepresented populations will broaden our understanding of genetic variants that influence drug responses and facilitate the clinical adoption of tailored dosing strategies to optimize therapeutic outcomes in diverse populations.

## VII Challenges, emerging approaches, and future directions

### A Challenges and solutions in pharmacogenomics of infectious diseases

The FDA reports that approximately 15% of drugs currently in the market include PGx information in their labeling. Moreover, between 2000 and 2020, the proportion of newly approved drugs that feature PGx-related details in their labeling nearly tripled, rising from 10.3% to 28.2%.^427^ The FDA has expanded its guidance on pharmacogenetic testing by adding dosing recommendations and warnings about drug response variability to the labels of over 400 drugs currently on the market.^428^ However, despite the growing inclusion of PGx data, its use in clinical decision-making remains limited.^429^^,^^430^ Several challenges still exist, including the limited diversity in genomic research and inadequate infrastructure for implementation. To ensure that PGx advancements benefit everyone, it is crucial to address these challenges and promote inclusivity in research and clinical settings.

A major challenge to implementing PGx is its cost-effectiveness. Despite significant reductions in genome sequencing costs in recent years, it remains relatively expensive. Because these tests are not typically included in standard care, insurance coverage is often unavailable, limiting patient access to genetic testing. Implementing PGx is costly and complex, even in high-income countries, making it even more challenging for healthcare systems in lower-income and less developed nations.^431^ The cost of PGx testing can vary widely depending on the country, healthcare system, and the specific test being conducted. In high-income regions such as the United States, preemptive genotyping for *HLA-B*∗57:01 is cost-effective, as long as the additional cost of alternative HAART treatment does not exceed $2419.80 per patient, based on a willingness-to-pay threshold of $40,000. For instance, in the United States, the cost of switching from abacavir-based regimens to nonabacavir regimens is $1485, which falls well below the threshold, thus supporting the use of *HLA-B*∗57:01 testing before initiating abacavir therapy. However, in low-income regions such as East Asia, parts of Africa, and West Asia (including countries such as Mali, Ghana, Saudi Arabia, China, Japan, and South Korea), the cost-effectiveness of preemptive genotyping is negative. This suggests that, for these regions, the cost of alternative HAART regimens would need to be lower than that of abacavir-based therapy for the genotyping strategy to be cost-effective.^432^ The low frequency of *HLA-B*∗57:01 in these populations leads to a higher number of patients needing testing to prevent a single ADR, which increases the cost burden. As such, reducing the cost of genotyping in these regions would likely improve the cost-effectiveness of pharmacogenomic testing. Moreover, the National Institutes of Health has recently allocated $42 million over 5 years to support several clinical trials under the Implementing Genomics in Practice initiative.^433^

According to the FDA, between 2015 and 2019, approximately 76% of clinical trial participants were of primarily European descent.^434^ The remaining participants were from other ancestries, with Asians making up 11% and Africans or African Americans comprising 7%. As a result, much of the data used in drug development is predominantly based on European populations, which may not fully represent individuals from other ancestral backgrounds. It is also important to note that these statistics did not differentiate between African American and Sub-Saharan African populations, which limits the ability to assess the extent of underrepresentation across different biogeographical regions in clinical trials.^435^ The generalizability of PGx findings is limited by the predominance of studies conducted on populations of European descent, underscoring the need for more diverse research cohorts.^436^ Over 63% of individuals represented in PharmGKB belong to European populations.^435^ According to global census estimates, Indigenous Americans, with approximately 62 million, represent a smaller population compared with the 2 billion people in Central and South Asia. However, South Asians are more underrepresented in PGx datasets relative to their population size than Indigenous Americans or any other biogeographical group.^435^ Studies have consistently demonstrated that nearly 96%–99% of healthy individuals possess at least one actionable PGx variant when assessed through multigene testing.^437^^,^^438^

In the field of PGx, populations that are less studied and have more diverse haplotype frequencies are more likely to experience inaccuracies in evidence-based drug dosage recommendations. For example, the indeterminate metabolizer status of variants in the *CYP2D6* gene, which affects the metabolism of approximately 25% of prescribed drugs, is more prevalent in Sub-Saharan Africans.^439^

The lack of standardized PGx tests and varying laboratory methods contributes to inconsistent PGx results. Public concerns about medical ethics, confidentiality, and potential misuse of genetic information further complicate patient participation.^440^ Moreover, limited knowledge and awareness of PGx among clinicians and patients-often because of insufficient training or familiarity- pose significant challenges. Addressing these social, ethical, and educational barriers is crucial for the effective integration of PGx into clinical practice.^441^

PGx of IDs offers significant potential for precision medicine but faces challenges, including limited research diversity and infrastructure, necessitating inclusive research and clinical practices to ensure widespread benefits. Moving forward, it is important to:

o Address the underrepresentation of non-European populations in genomic research, through initiatives such as the "Qatar Genome Program" and the US “All of Us.” Replicating such inclusive efforts globally will enhance our understanding of genetic variations and their impact on drug responses across diverse populations. It is important to promote inclusive trial design by establishing diversity enrollment targets and ensuring proportional representation of underrepresented populations.
o Support capacity-building efforts in low- and middle-income countries by establishing trial sites, training local investigators, and ensuring equitable access to trial participation.
o Invest in PGx infrastructure to realize the full potential of PGx and ensure its benefits extend to diverse populations. For instance, the “RAFAgene” project is exploring the role of genetic variations in influencing PK differences and toxicity in patients being treated for multidrug resistant tuberculosis in sub-Saharan Africa.^442^
o Foster global data-sharing collaborations and harmonization of trial protocols to maximize the inclusion of diverse populations while respecting ethical and legal standards across regions.
o Invest in efficient PGx testing processes and integrate genetic data with electronic health records.
o Invest in advanced genetic testing technologies, such as next-generation sequencing and long-read sequencing, which offer faster, more accurate, and cost-effective detection of PGx variants, including rare and structural variations. Additionally, developing custom PGx chips tailored to specific populations can efficiently genotype clinically actionable, prevalent variants in particular ethnic groups, enabling cost-effective, targeted testing that maximizes clinical relevance.
o Explore the use of AI and ML to analyze vast amounts of electronic health record data linked to DNA biobanks, enabling accurate identification of PGx markers of immune-mediated diseases and ADRs.
o Design predictive models that identify people at risk of particular immune-mediated diseases by utilizing deep phenotyping from electronic health record data. This enables earlier interventions and individualized treatment approaches.

### B Integration of pharmacogenomics with other omics data

Association and observational cohort studies have played a crucial role in identifying genetic markers linked to variability in drug efficacy and toxicity. However, moving from association to mechanistic investigation that can inform causal inference requires integrating multiomics to identify biological pathways. Additionally, experimental validation using cellular and animal models is instrumental in confirming causal inferences by elucidating the direct biological effects of candidate variants and pathways. A holistic approach that integrates pharmaco-transcriptomics, pharmaco-proteomics, and pharmaco-metabolomics with pharmaco-genomics offers significant advantages in understanding and optimizing drug responses.^443^

Several transcriptomic markers have been linked to the effectiveness of TB treatment. For example, the expression levels of C2, BF, and Complement C1q, along with serpin G1, have been associated with treatment efficacy.^444^ Other genes, such as Pragmin, have been implicated in predicting the risk of relapse after treatment, whereas UCP2 is associated with treatment outcomes, influencing the overall therapeutic approach.^445^^,^^446^ Inflammation biomarkers, including GBP4, IL-15RA, and UBE2L6, have been identified as significant indicators for TB therapy response.^447^ Moreover, the transcription factors KLF2, GBP5, and DUSP3 are important for understanding individual variation in response to treatment.^448^

Proteomic profiling has also revealed crucial biomarkers related to treatment outcomes. Angiotensinogen and Complement component C7 are associated with treatment effectiveness, whereas phosphoryl-transfer RNA kinase has been linked to treatment efficacy as well.^449^^,^^450^ A broader panel of proteins, including Coagulation factor V, serum amyloid A, IGFBP1, CATZ, ECM1, and YES, has been identified as a biomarker for treatment response.^451^ Eotaxin, a protein associated with immune cell trafficking, has also been shown to correlate with the therapeutic response in patients with TB.^452^ Proteomic profiling is not only beneficial for predicting treatment response, but also for predicting HSRs, as reported in a study on abacavir HSS in *HLA-B*∗57:01 carriers.^129^ By analyzing differences in protein expression related to immune response pathways, researchers can develop more precise assays to differentiate drug-sensitive from drug-tolerant individuals.

Metabolomic profiling has revealed metabolic signatures associated with treatment response. For example, L-cysteine-glutathione disulfide, arachidonic acid, L-histidine, and biliverdin are metabolites that correlate with treatment outcomes in TB.^453^ Pyridoxate, a metabolite related to vitamin B6 metabolism, has also been associated with therapeutic success in TB.^454^ Additionally, bradykinin and desArg9-bradykinin levels have been shown to affect treatment responses, suggesting a role for these metabolites in immune regulation during TB therapy.^455^ These findings underscore the importance of integrating multiomics data in PGx to develop more effective, personalized treatments for IDs such as TB. Researchers can gain a comprehensive understanding of how genetic variants impact drug metabolism across multiple levels, including gene expression, protein function, and metabolite profiles, by integrating different omics disciplines.

Pharmaco-proteomics offers insights into protein interactions and changes that impact drug efficacy and toxicity, whereas pharmaco-transcriptomics demonstrates how genetic differences impact gene expression linked to drug responses.^456^ This is further enhanced by pharmaco-metabolomics, which profiles metabolic alterations related to medication delivery.^457^ When combined, these integrated data provide a more comprehensive picture of how drugs behave in the body, which can help with individualized treatment regimens, more accurate drug response forecasts, and better therapeutic outcomes. This multimodal method advances our understanding of individual differences in medication responses and opens the door to more personalized and effective PGx approaches. Although studies have shown the potential of integrating PGx with multiomics to identify novel biomarkers, real-world clinical case studies are needed to translate this data into treatment adjustments, dosing strategies, or ADR prevention.

Mathematical models are increasingly recognized for their valuable contributions to the field of IDs. These predictive models offer a framework for comprehending the initiation, progression, and outcomes of diseases. By leveraging integrated datasets from various “omics” technologies, comprehensive host-pathogen interaction networks can be constructed. Analyzing these models helps elucidate the complex molecular processes within microbial organisms and their interactions with the host.^458^

### C Utilization of artificial intelligence and machine learning

**Fig. 5:**
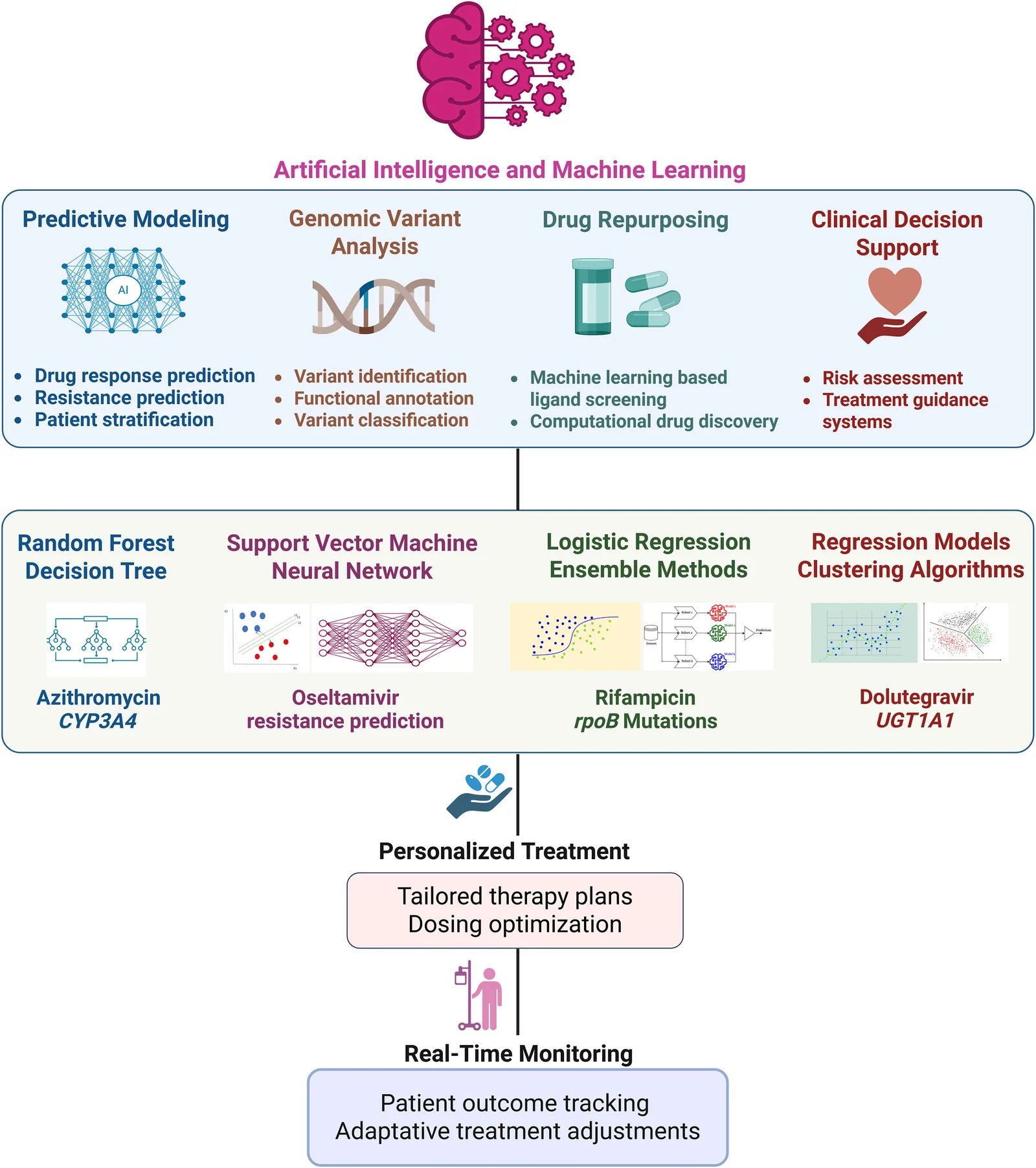
Application of machine learning in PGx of ID. Applications of AI and ML in PGx include predictive modeling, genomic variant analysis, drug repurposing, and clinical decision support. This enables personalized therapy plans, ultimately improving clinical outcomes. Created in BioRender. Smatti, M. (2026) https://BioRender.com/pg1sifw.

Artificial intelligence (AI) and ML have proven for highly effective in PGx for forecasting drug responses across a range of therapeutic domains, including depression, anticoagulant therapy, and cancer. Random forest (RF) is the most widely used supervised learning technique because it builds numerous decision trees on various data subsets and combines their outputs, which successfully reduces variance and overfitting. Additionally, RF can handle continuous and categorical variables and is typically robust to outliers. On the other hand, unsupervised learning techniques are not yet widely applied in this domain. Their prospective advantages are encouraging, though. When studying pharmacological responses, starting with unsupervised learning analysis can help find subgroups that are more balanced and less arbitrary than those found in traditional response classifications. For example, a study on the pharmacogenetics of antidepressant response compared various supervised techniques, including neural networks (NNs), recursive partitioning, learning vector quantization, gradient-boosted machines, and random forests. The study involved 671 adult patients from 3 European studies on major depressive disorder, with NN achieving the highest accuracy among the tested models.^459^ Moreover, other studies on warfarin dosing have used various supervised machine learning techniques, including NNs, ridge regression, RFs, support vector regression, and the least absolute shrinkage and selection operator regression, demonstrating significant improvements in prediction accuracy over standard methods.^460^, ^461^, ^462^, ^463^ Another study, which examined warfarin stable dosage prediction using 7 different supervised machine learning models (multiple linear regression, NN, regression trees, support vector regression, and RF), found that multiple linear regression remained the most effective model for the study population.^464^ Furthermore, a study involving 186 patients with major depressive disorder aimed to predict the response to antidepressants and compared the performance of random forests and support vector machines (SVM). SVM demonstrated superior performance in predicting antidepressant response. Additionally, the researchers applied the least absolute shrinkage and selection operator regression for feature selection, identifying 19 of the most robust SNPs. Supervised machine learning techniques also helped differentiate between remitters and nonremitters in response to antidepressants.^465^ Hence, in the future, pharmacogenomic research should focus on leveraging AI and ML to analyze complex genetic data (Fig. 5). These technologies can identify intricate patterns and genetic markers, leading to more precise predictions of drug responses and personalized treatment strategies.

Although AI and ML hold great promise for advancing PGx, their clinical implementation comes with its own challenges. These include data privacy and confidentiality concerns related to secure storage, access rights, and the potential misuse of sensitive information. Accordingly, it is important to have robust plans for data protection and consent processes to ensure the ethical and effective use of AI tools. Another concern is the interpretability of AI models, especially complex ones, which need to be explained clearly to clinicians. Building explainable AI approaches that enable end-users to comprehend and interpret the outputs and predictions made by the models will be critical for the successful implementation of AI-derived PGx data.^466^

## VIII Conclusion

PGx of IDs has evolved considerably over the last decade. The accumulation of robust scientific evidence for selected gene–drug pairs has enhanced its clinical utility. Nevertheless, there remains a substantial knowledge gap in the PGx of currently used antibiotics and antivirals. Given the existing knowledge from well studied PGx markers of drug metabolism and adverse events, it is of a great interest to explore their relevance to important anti-infective agents, through genomics and mechanistic studies.

Leveraging advances in large-scale data generation and integrative analytics represents a key strategy to maximize the impact of PGx research in IDs. High quality evidence on host genomic markers, when combined with pathogen factors and funneled into clinically relevant guidelines, has the potential to shift ID management toward a patient-tailored care. Preemptive PGx screening for selected clinically actionable variants has demonstrated clinical value and cost-effectiveness in several therapeutic areas and may offer similar benefits in appropriately defined ID contexts.

Additionally, patients and their families should be informed about the role of PGx variability in drug response and toxicity and the potential advantages of incorporating novel antiviral agents to support shared decision-making, particularly in cases of severe or resistant infections. Ultimately, continued rigorous validation and thoughtful clinical integration will determine where PGx can deliver the greatest impact in precision IDs therapeutics.

## Conflict of interest

The authors declare no conflicts of interest.
